# Identification and analysis of brown planthopper-responsive microRNAs in resistant and susceptible rice plants

**DOI:** 10.1038/s41598-017-09143-y

**Published:** 2017-08-18

**Authors:** Yan Wu, Wentang Lv, Liang Hu, Weiwei Rao, Ya Zeng, Lili Zhu, Yuqing He, Guangcun He

**Affiliations:** 1 National Key Laboratory of Hybrid Rice, College of Life Sciences, Wuhan University, Wuhan, 430072 China; 2 Shandong Rice Research Institute, Shandong Academy of Agricultural Sciences, Jinan, 250100 China; 3 National Key Laboratory of Crop Genetic Improvement, Huazhong Agricultural University, Wuhan, 430070 China

## Abstract

The brown planthopper (BPH) is the most devastating insect pest of rice. The rice gene *BPH15* confers resistance to BPH. MicroRNAs (miRNAs) regulate a spectrum of development and defense response processes in plants. In this study, we analyzed six miRNA profiles of a *BPH15* introgression line (P15) and a susceptible recipient line (PC) at three time points (0 h, 6 h and 48 h) after BPH attack, and identified 464 known miRNAs and 183 potential novel miRNAs. Before the BPH feeding, we identified 23 miRNAs differentially expressed in P15 and PC. We speculated that the resistant plant is in a priming state by the regulation of miRNAs. After the BPH feeding, 104 miRNAs were found to be expressed differentially in P15 (68 in P15-6/P15-0, 36 in P15-48/P15-0), and 80 miRNAs were found expressed differentially in PC (32 in PC-6/PC-0, 48 in PC-48/PC-0), which illustrated that miRNA expression is activated upon attack. These miRNAs regulate different pathways that contribute to the basal defense and specific resistance of rice to the BPH. Our study provides additional data for scientists to further explore the mechanism of plant defense against insect attack and to find a way for efficient insect control.

## Introduction

Plants and herbivorous insects have been engaged in a perpetual evolutionary battle for at least 100 million years. Plants have evolved multiple mechanisms to detect insects and activate defense. Some evidence indicates that plants’ defense to insects shares many similarities with their defense to pathogens^1, 2^. However, since insects’ behavior is more complicated than that of pathogens, the interaction between plants and insects is more intricate^3^. Insects cause direct and indirect damage when feeding on plants. Plants perceive chemical elicitors, mostly from insect saliva, and initiate signaling events such as calcium release, oxidative burst, the activation of several protein kinases, and JA synthesis and then induce the production of toxic secondary metabolites, volatile organic compounds, and other compounds to provide effective protection against insects^4^.

MicroRNAs (miRNAs) are endogenous non-protein coding small RNAs present in both plants and animals that generally comprise 21–24 nucleotides (nt) and are known to play important roles in regulating the expression of protein-coding genes at the post-transcriptional level^5^. Plant miRNAs play a significant role in many plant development processes such as leaf development^6^, floral development and vegetative phase change^7, 8^, shoot and root development^9, 10^, vascular development^11^, and hormone signaling pathways^9, 10, 12^. The role of miRNA in stress responses should not be underestimated. Over 200 recently published studies of more than 40 plant species have reported a role for miRNAs in regulating the plant responses to 35 abiotic stresses such as drought, cold and high salt concentrations^13^. miRNAs have also been reported to regulate plant responses to biotic stresses. They participate in the regulation of the defense response to the bacterial biotroph *Pseudomonas syringae* ^14, 15^, the pathogen-associated molecular pattern (PAMP) flg22^16^, fungal infections^17^, and *Turnip mosaic virus* infection^18^. The miRNA response to insect herbivory in plants is not as well understood. Silencing *RdR1* (RNA-directed RNA polymerase 1) and the DICER-like genes *DCL3* and *DCL4* increased susceptibility to insect herbivory in *Nicotiana attenuata*, showing that a group of miRNAs are involved in *N. attenuata*’s responses to herbivore attack^19, 20^.

The brown planthopper (BPH), *Nilaparvata lugens* (Stål) (Hemiptera: Delphacidae), is the most devastating insect pest of rice in the modern rice cultivation system and causes the most serious damage to the rice yield as well as large economic losses. It is a monophagous piercing-sucking herbivore insect and sucks the sap from the rice phloem using its stylet. BPH causes direct damage to rice plants and transmits viral diseases^3^. To date, 30 BPH resistance genes have been detected in cultivated *O. sativa* and wild species of rice^21^. The BPH resistance gene *BPH15* was identified from wild rice (*Oryza officinalis* Wall. ex Watt.)^22, 23^. Recently, we have cloned a lectin receptor-like kinase that contributes to *BPH15* resistance^24^. Among the three dominant BPH-resistance genes (*BPH14, BPH15, BPH18*) that have been introgressed into the elite indica rice 9311 and hybrid rice, *BPH15* had the greatest effect on conferring resistance to BPH^25, 26^. This indicates *BPH15’*s potentially important contribution to the agricultural industry with its unique mechanism of resistance to BPH.

In this study, we focused on the miRNA responses in the rice plants to BPH attack. We identified a number of miRNAs that are expressed differentially in the resistant (a *BPH15* introgression line) and susceptible plants (recurrent parent 9311) before and after insect feeding^25^, and these miRNAs regulated different pathways that contribute to the basal defense and specific resistance of rice to BPH insects. The results indicate that miRNA might play important roles in rice defense against BPH.

## Results

### An overview of the small RNA sequencing results

**Figure 1:**
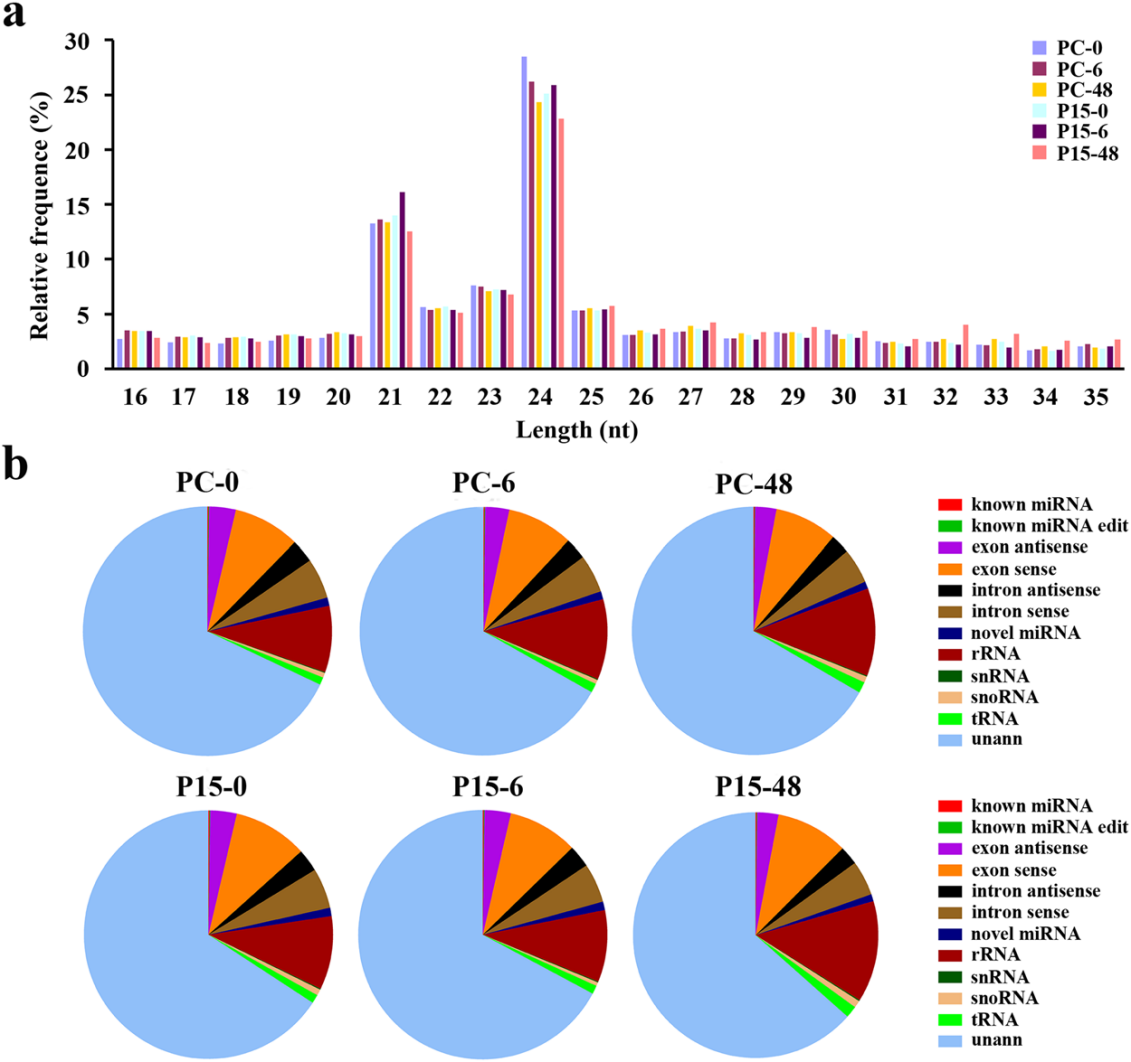
Size distribution and annotation of small RNAs from the libraries of the resistant *BPH15* introgression line (P15) and susceptible recipient line (PC) at 0 h, 6 h, and 48 h after BPH infestation. (**a**) Length distribution of sequenced reads. The most abundant sRNAs in both libraries were 21 nt and 24 nt in length. (**b**) Proportions of different classes of small RNAs detected in the six libraries.

To clarify the role of miRNAs in the rice defensive reaction to BPH, we performed the deep sequencing and characterization of the small RNAs (sRNA) in the *BPH15* introgression line (P15) and susceptible recipient line (PC) infested by BPH insects for 6 h (P15-6, PC-6), 48 h (P15-48, PC-48) and non-infested (P15-0, PC-0). Total reads of 18,713,895 (PC-0), 18,298,038 (PC-6), 21,032,597 (PC-48), 20,223,463 (P15-0), 18,599,929 (P15-6) and 18,215,166 (P15-48) were generated in the six libraries (Table 1). After removing the sequences of low quality, poly A, incorrect adaptors and those shorter than 18 nt, 13,862,467 (PC-0), 13,108,056 (PC-6), 14,327,775 (PC-48), 13,972,099 (P15-0), 13,081,158 (P15-6) and 13,742,651 (P15-48) clean reads were obtained. The length distribution of the sRNA, as shown in Fig. 1a, was mostly concentrated at 21 nt and 24 nt as previously reported for rice sRNAs. Then, all of the sequences were aligned with small RNAs in GenBank database (release 209.0) and Rfam database (release 11.0) to identify and remove rRNA, scRNA, snoRNA, snRNA and tRNA. The unique sequences obtained were then mapped to the rice genome. Those mapped to exons or introns and repeat sequences were also removed (Fig. 1b). The remaining sequences were assigned to the miRNA database in miRBase (release 21). Rice miRNA is the most thoroughly studied monocot miRNA, and there are 592 precursors and 713 mature miRNAs in miRBase. We identified 464 known miRNAs. Most of the known miRNAs are 21 nt-long and terminated in a 5′-U residue, consistent with the characteristics of Ago2 protein (Fig. S1).

**Table 1:** Summary of small RNA sequences data.

Type	PC-0	PC-6	PC-48	P15-0	P15-6	P15-48	Total
Total_reads	18713895	18298038	21032597	20223463	18599929	18215166	115083088
High quality	16046366	15456853	17532726	16974946	15595125	15773774	97379790
3′ adapter null	307480	78649	732850	270039	49618	309152	1747788
Insert null	39987	38526	44154	45743	88276	62704	319390
5′ adapter contaminants	66406	72180	78407	102359	77793	50050	447195
Smaller than 18 nt	1769541	2159036	2349106	2584275	2297919	1608800	12768677
Poly A	485	406	434	431	361	417	2534
Clean reads	13862467	13108056	14327775	13972099	13081158	13742651	82094206

**Figure 2:**
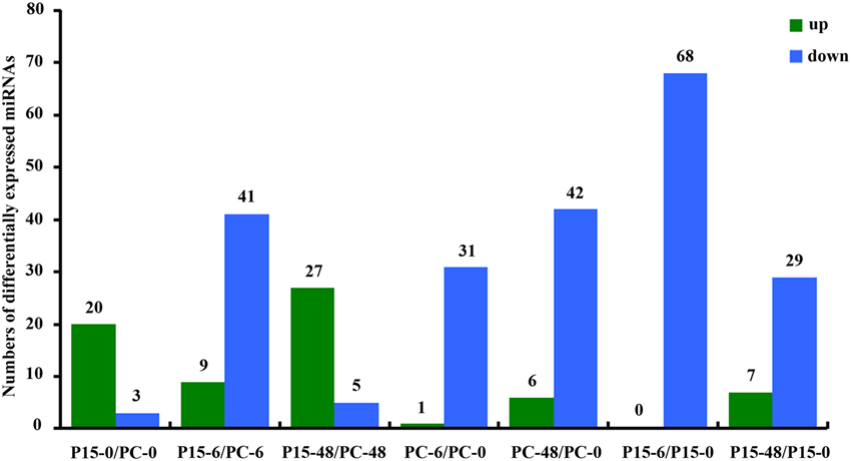
Contrast between up-regulated and down-regulated differentially expressed miRNAs in all comparisons. Differential expression analyses were run with the Bioconductor edgeR package with the condition that the ratio was greater than 2 and P < 0.05. Up represents the number of miRNAs that were up-regulated in the compared group, and down represents the number of miRNAs that were down-regulated in the compared group.

**Figure 3:**
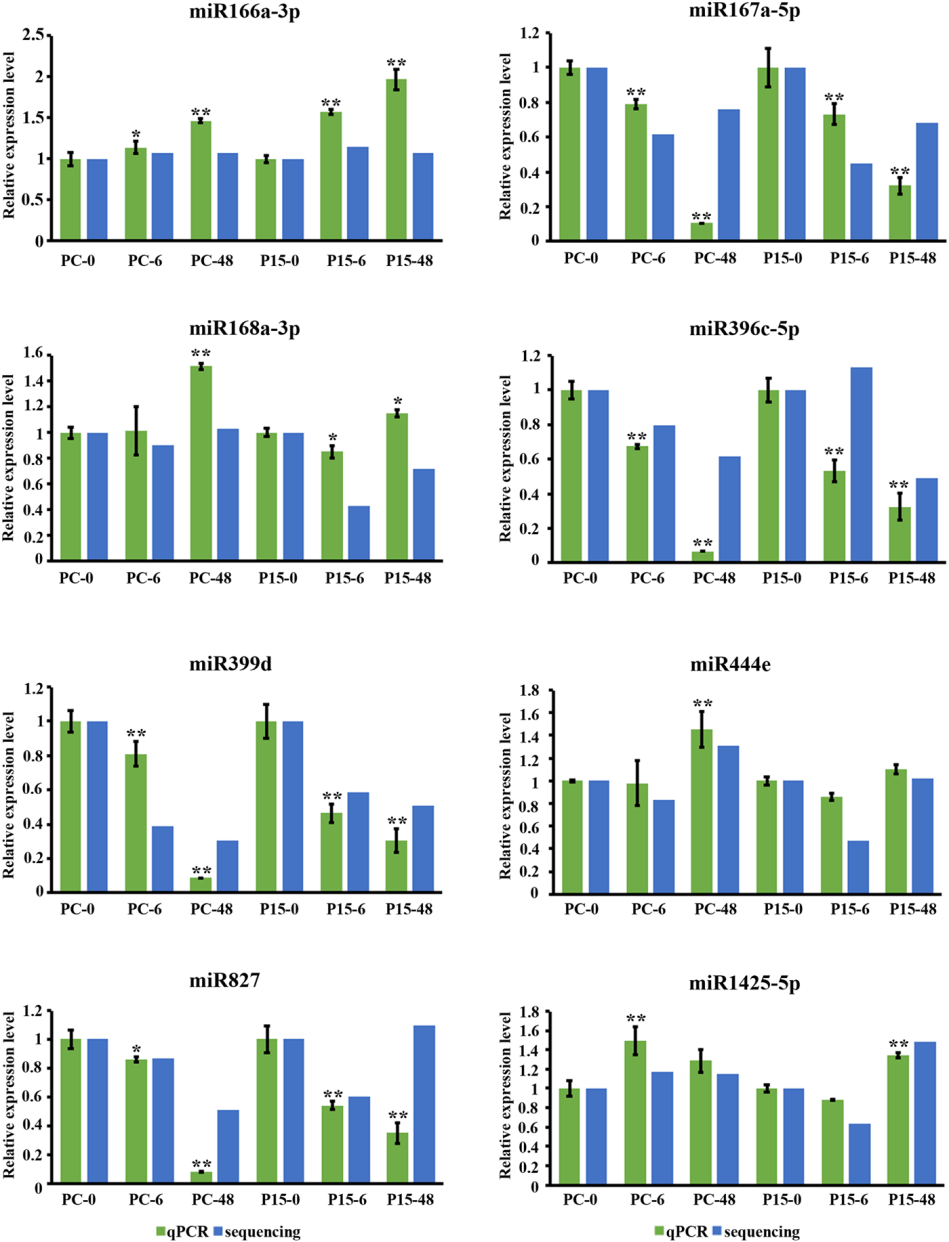
Stem-loop RT-PCR analysis of known miRNA levels in P15 and PC compared to sequencing results. The expression of miRNAs was normalized by small RNA U6. The data represent the mean ± SD from three biologically independent experiments. Statistical significance was analyzed using one-way ANOVA. The asterisks represent significance, where one asterisk indicates P ≤ 0.05, and two asterisks indicates P ≤ 0.01.

The expression level of a miRNA in one treatment is indicated as the miRNA sequencing reads in the total reads^27^. The expression levels of 464 miRNAs were compared between the treatments. The numbers of miRNAs with a difference ratio greater than 2 and a P < 0.05 are shown in Fig. 2. There were 104 miRNAs differentially expressed in P15 (68 in P15-6/P15-0, 36 in P15-48/P15-0) and 80 miRNAs differentially expressed in PC (32 in PC-6/PC-0, 48 in PC-48/PC-0), which illustrates that the miRNAs did respond to BPH feeding, and more miRNAs responded in P15 than in PC. In addition, 23, 50 and 32 miRNAs were differentially expressed in P15-0/PC-0, P15-6/PC-6 and P15-48/PC-48, respectively. Forty-one of the 50 were down-regulated in P15-6/PC-6, while 27 of the 32 were up-regulated in P15-48/PC-48. All this suggests that there are different regulation modes in P15 and PC, and the early (6 h) and late (48 h) responses to BPH resistance were different, particularly in the resistant rice. We validated eight of the differentially expressed miRNAs by quantitative stem-loop RT-PCR^28^, and the results of the three biological replicates were consistent with the log_2_ ratios of mature miRNA counts (Fig. 3).

### Identification of novel candidate miRNAs in BPH-infested rice

**Figure 4:**
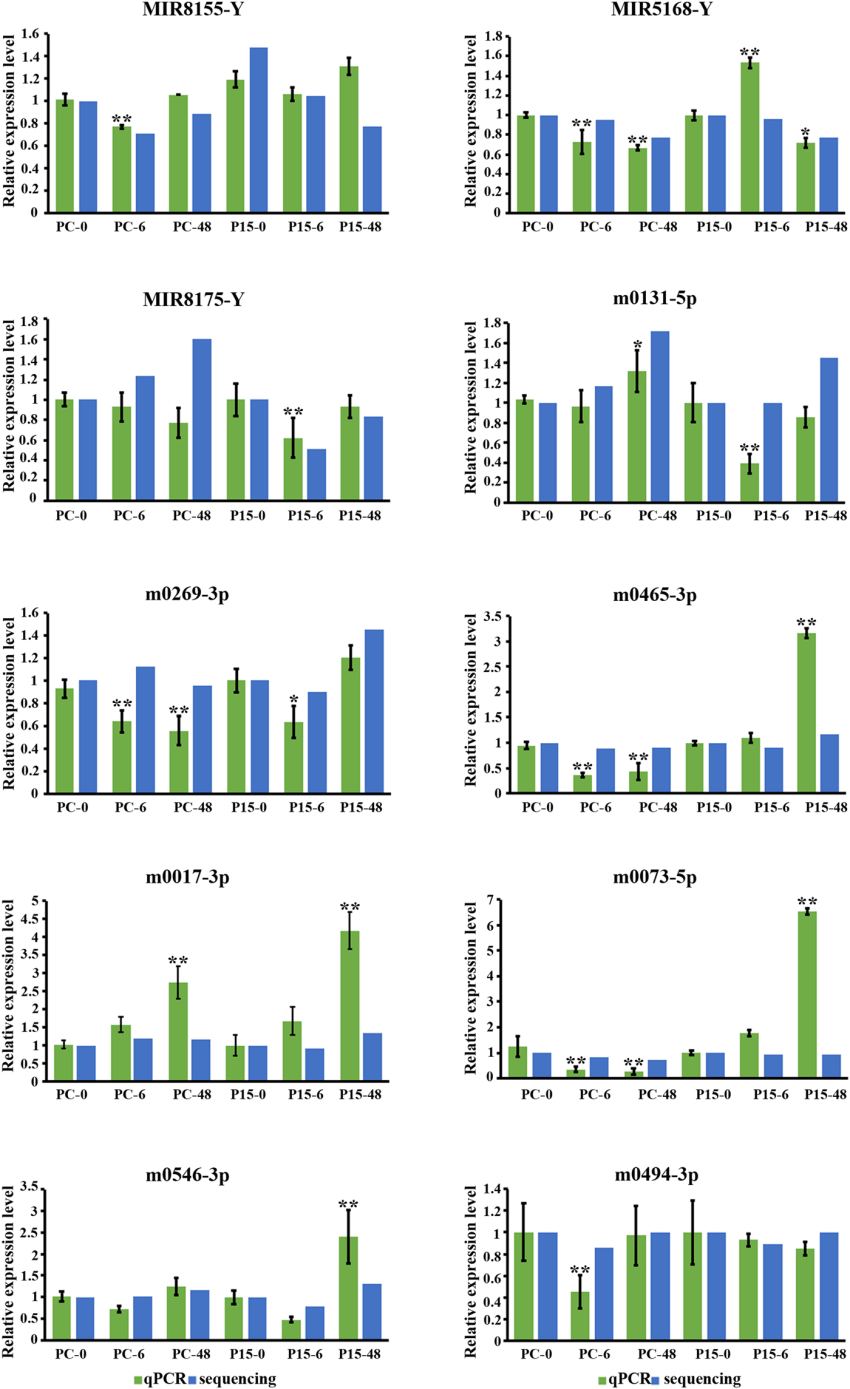
Stem-loop RT-PCR analysis of novel miRNA levels in P15 and PC compared to sequencing results. The expressions of miRNAs was normalized by small RNA U6. The data represent the mean ± SD from three biologically independent experiments. Statistical significance was analyzed using one-way ANOVA. The asterisks represent significance, where one asterisk indicates P ≤ 0.05, and two asterisks indicates P ≤ 0.01.

In addition to the known miRNAs, novel miRNAs were also found in the high-throughput sequencing data. First, we compared our sequencing data to the miRNAs of selected plants in miRBase and obtained 739 miRNAs, of which 85 miRNAs were represented by total reads ≥ 100 in all libraries (Table S1). An analysis of the nucleotide sequences of these miRNAs revealed that the first base of the 5′-terminus did not have a preference for uridine (U) (Fig. S2). We selected three highly expressed miRNAs (MIR8155-Y, MIR5168-Y and MIR8175-Y) for a qPCR experiment, two of which (MIR5168-Y and MIR8175-Y) were similar to the sequencing data (Fig. 4).

miRNA has a special secondary structure, so by referring to the genome sequence of rice, we can locate the remaining sequences on the genome and find possible novel miRNAs secondary structure prediction. Then, by aligning the sequences to the rice genome with no nucleotide mismatch and the predicted characteristic stem-loop precursor, we found 695 novel miRNAs, of which 98 were represented by total reads ≥ 100 in all libraries (Table S2). The first base of the 5′ end had a preference for U (Fig. S3). However, when we validated the predicted novel miRNAs by quantitative stem-loop RT-PCR, only one of seven miRNAs, novel-m0494-3p, had a similar expression to the sequencing data (Fig. 4). The secondary structures of the seven novel miRNA precursors are shown in Fig. S4. The low expression level may explain why accurate detection was not possible with qPCR. The Cq values of these genes were almost 35 cycles. In addition, as a model plant, rice has been found to possess many miRNAs. The remaining novel miRNAs expressed at a low level may not have been detected accurately by qPCR, so we need other means to confirm their existence.

### miRNAs differentially expressed in P15 and PC before BPH feeding

To determine whether the expression of miRNAs in resistant rice was different from that in susceptible rice before BPH attack, we compared the miRNAs of P15-0 and PC-0, and found that for almost 90% of the differently expressed miRNAs, the expression in P15-0 was higher than that in PC-0. As shown in Table 2, there were 20 up-regulated and three down-regulated miRNAs in P15-0/PC-0. Among those miRNAs, many members were reported to be involved in abiotic stress responses to drought, salt, or other harmful stimuli. Osa-miR531 was reported to target the members of the MAPK cascade gene family, which plays an important role in plants’ innate immunity^29^. The expression of miR531 was enhanced in P15. Therefore, this targeting could affect plant responses to BPH through the MAPK cascade. Osa-miR3979-3p is a H_2_O_2_-response miRNA, and one of its targets is a putative NBS-LRR disease resistance gene^30^. We speculate that this may play an important role in the signal transduction of BPH resistance.

**Table 2:**
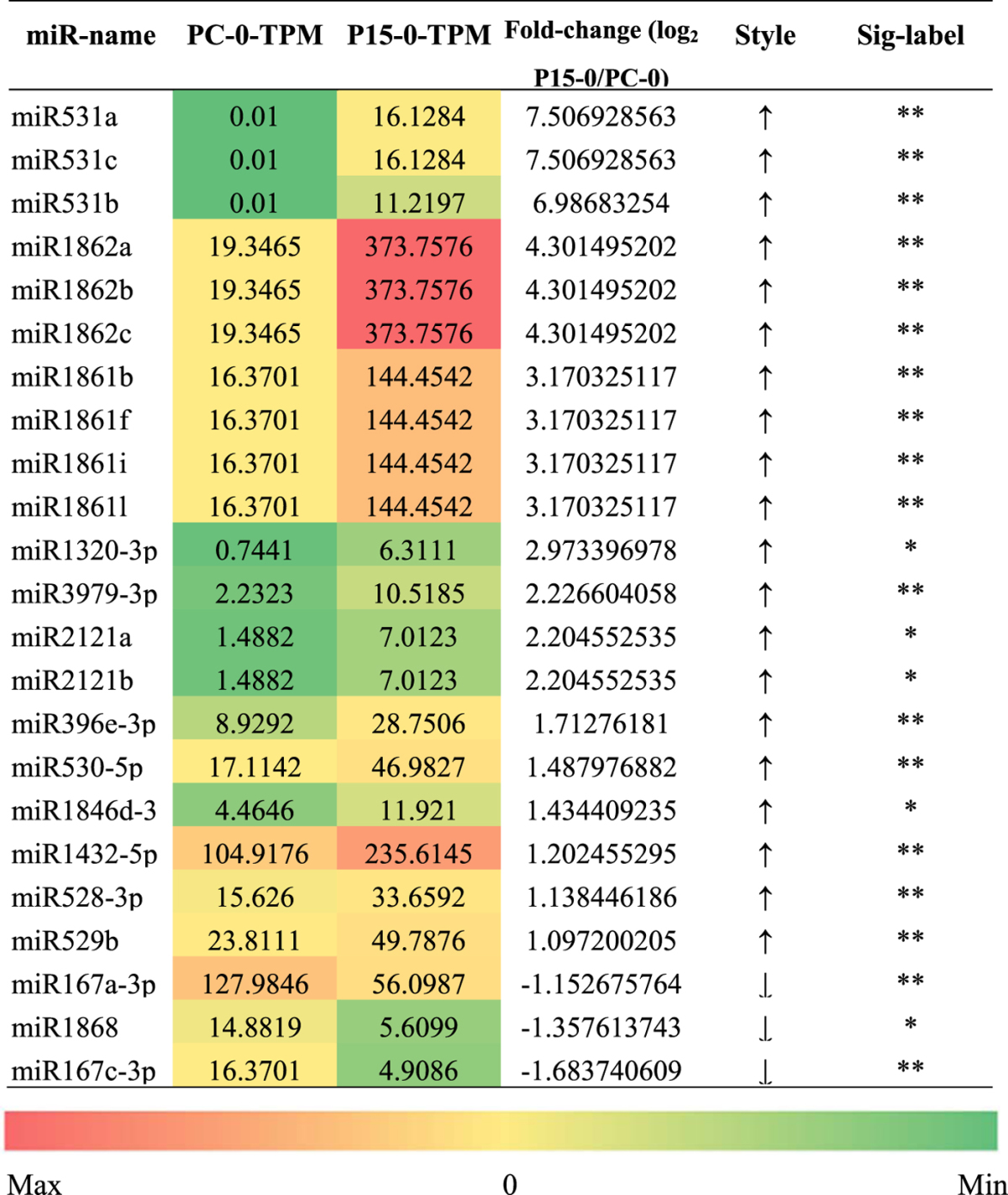
Differentially expressed miRNAs in P15-0/PC-0. The TPM was normalized miRNA sequence reads. Style “↑” indicates that the miRNA was up-regulated in P15-0/PC-0, and style “↓” indicates that the miRNA was down-regulated in P15-0/PC-0. Statistical significance was analyzed by Bioconductor edgeR package. Significant differences are indicated by *P < 0.05, **P < 0.01.

**Figure 5:**
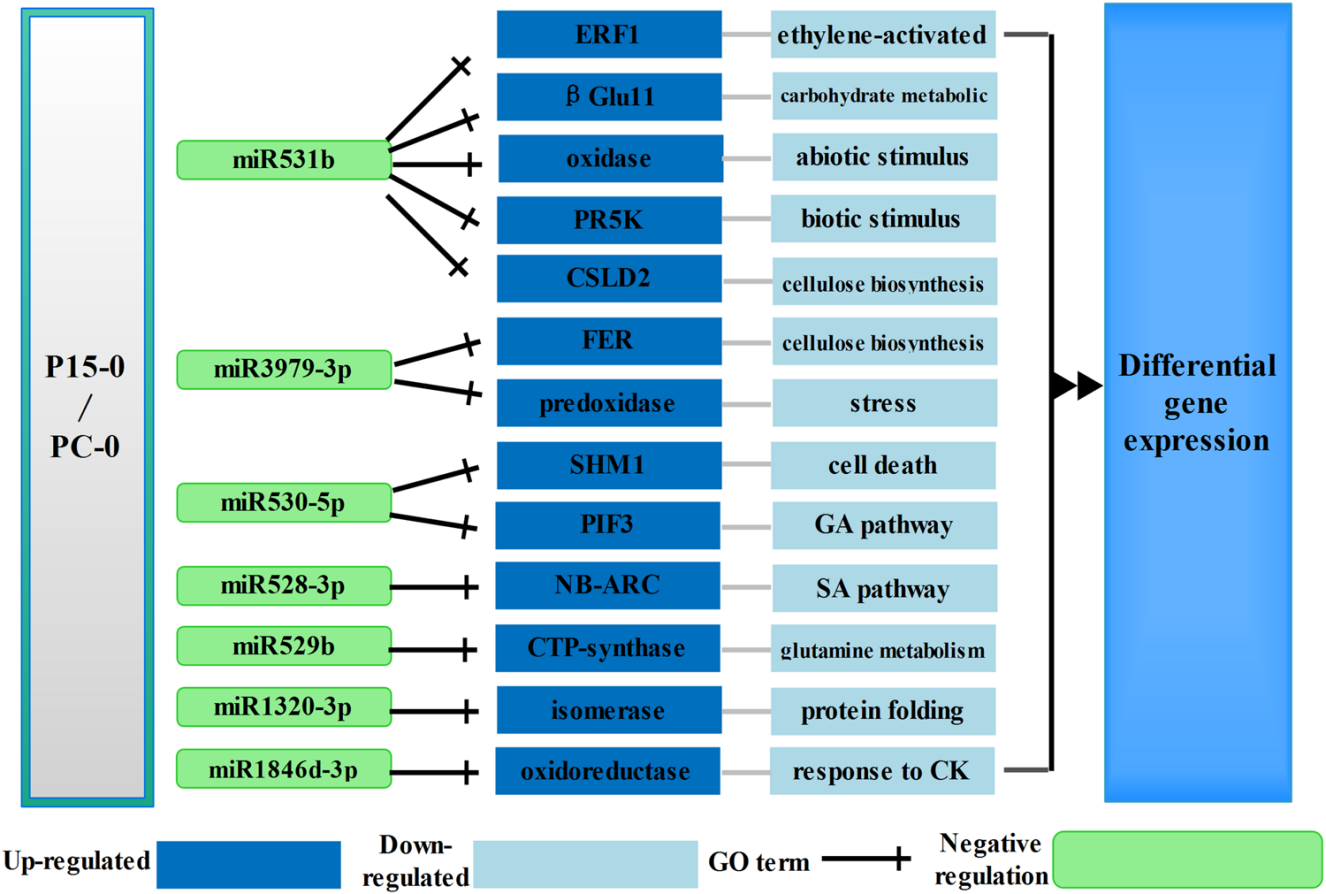
Regulatory network of two rice genotypes before BPH attack. ERF1: ethylene response factor 1; B-Glu11: beta glucosidase 11; oxidase: NADPH/respiratory burst oxidase protein D; PR5K: PR5-like receptor kinase; CSLD2: cellulose-synthase like D2; FER: malectin/receptor-like protein kinase family protein; Peroxidase: peroxidase superfamily protein; SHM1: serine transhydroxymethyltransferase 1; PIF3: phytochrome interacting factor 3; NB-ARC: NB-ARC domain-containing disease resistance protein; CTP synthase: CTP synthase family protein; isomerase: cyclophilin-like peptidyl-prolyl cis-trans isomerase family protein; oxidoreductase: FAD/NAD(P)-binding oxidoreductase.

To identify the target of these miRNAs, we used patmatch software and predicted that 136 rice genes were the potential targets of the 23 differently expressed miRNAs (Table S3). Previous work suggests that the expression of a target gene should exhibit the opposite pattern to the corresponding miRNA^31^, and we have previously performed RNA-seq when sequencing miRNA^32^. Therefore, we checked the RNA-seq results and selected 67 potential targets whose expression patterns were the opposite of eight of the miRNAs (Table S3). Among the selected 67 potential targets, we found a number of genes related to plant resistance responses (Fig. 5), including the genes belonging to the GO terms of abiotic and biotic stimuli (RHD2/ LOC_Os12g35610, PR5K/ LOC_Os01g02310), regulation of plant hormones including GA, SA, ET, and CK (PIF3/LOC_Os07g05010, NB-ARC/LOC_Os09g13820, ERF1/LOC_Os05g37640, oxidoreductase/LOC_Os02g22260), cellulose biosynthesis (CSLD2/LOC_Os07g36700, FER/LOC_Os01g56330), amino acids synthesis (CTP synthase/LOC_Os05g49520) and protein folding (isomerase/LOC_Os08g19610). More interestingly, most of these GO terms have been reported to play a pivotal role in the resistance to BPH^3, 32^. The results indicate that these differentially expressed miRNAs are important in the *BPH15* introgression line prior to BPH feeding. By the regulation of the miRNAs, the resistant plant is in a priming state^33^.

### Common defense-related miRNA in two rice genotypes at 6 h and 48 h after BPH-feeding

**Figure 6:**
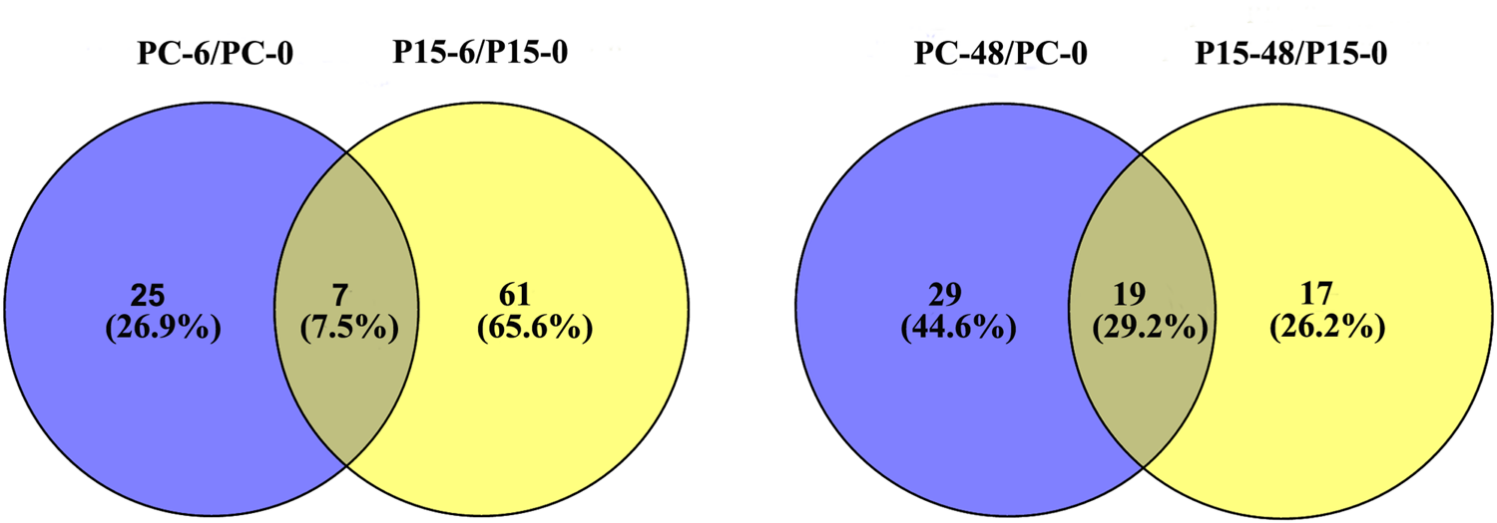
Venn diagrams of differentially expressed miRNA candidates. Venn diagram of the number of differently expressed miRNA molecules of the resistant *BPH15* introgression line (P15) and susceptible recipient line (PC) at 0 h, 6 h and 48 h after BPH infestation.

**Figure 7:**
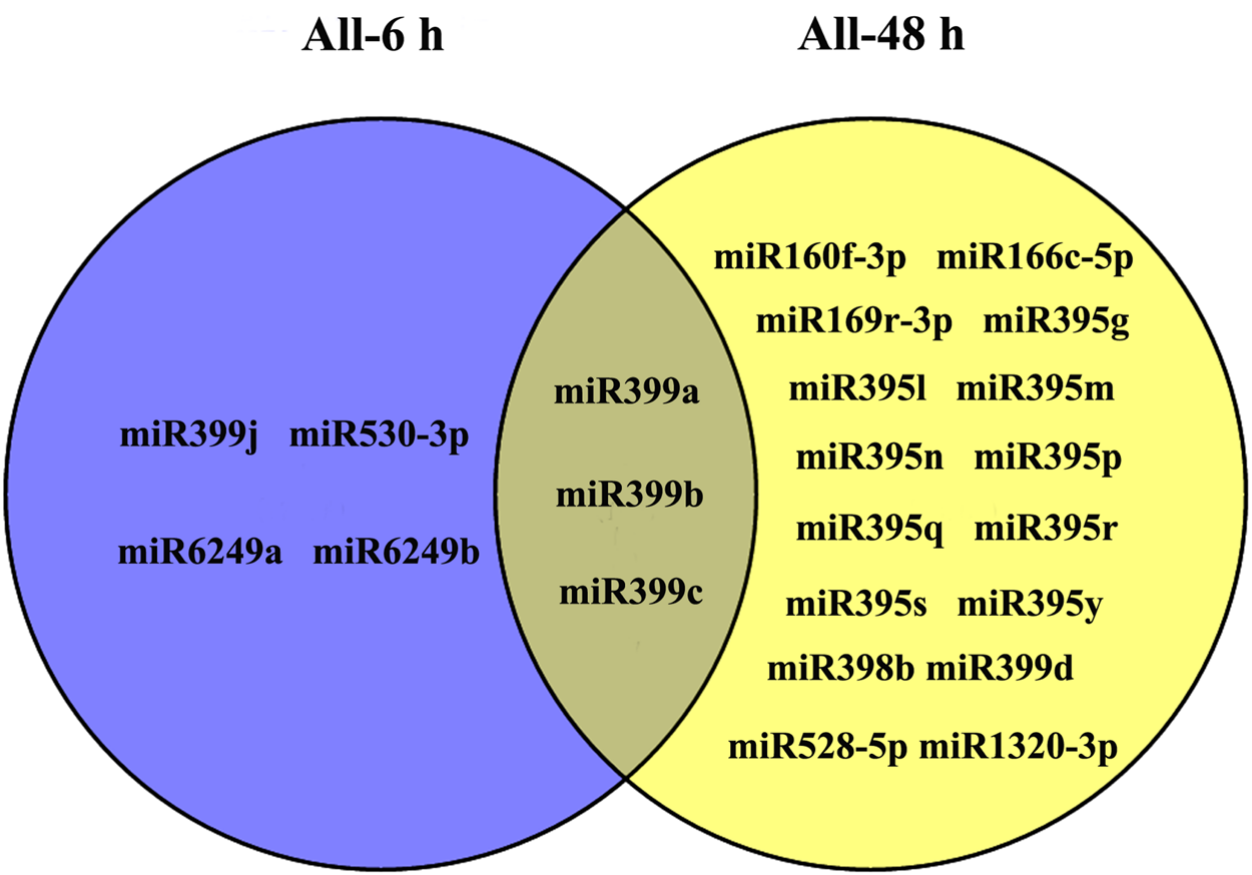
Venn diagram of common defense-related miRNAs in two rice genotypes 6 h and 48 h after BPH feeding.

A number of differentially expressed miRNAs appeared in both rice genotypes, seven of which appeared in the two comparisons at 6 h (PC-6/PC-0, P15-6/P15-0), and 19 of which at 48 h (PC-48/PC-0, P15-48/P15-0) (Fig. 6). Among these miRNAs, miR528-5p, miR398b, and miR166c-5p were up-regulated, while all the others were down-regulated (Fig. 7). Although miR398b was up-regulated in the two lines, the increase was more significant in the susceptible line. The expression level of miR398b was approximately 1.45-fold higher in the PC than in the P15 at 48 h, meaning that the two miR398b target genes encoding superoxide dismutase (CSD1 and CSD2) were probably expressed more in P15 than PC. On the other hand, miR160f-3p, miR166c-5p and miR169r-3p were down-regulated in the two rice genotypes at 48 h. Since osa-miR160, osa-miR166 and osa-miR169 were previously reported to respond to auxin^34^, GA^35^ and ABA^36^, respectively, they probably modulate the genes involved in hormone pathways^37^. Our result indicated that miR160f-3p, miR166c-5p and miR169r-3p might affect the plant responses to BPH through regulating the genes of the signaling pathways of JA-modulating hormones^37^. The remaining miRNAs, including miR530-3p, miR395g/l/m/n/p/q/r/s/y, miR528-5p, miR399a/b/c/d/j, miR1320-3p and miR6249a/b, were shown to respond to certain abiotic threats^36, 38–40^, and based on this study, these miRNAs may respond to BPH attack. We propose that although the recognition of BPH can be very specific, plants have a “common downstream signaling machinery” that is activated upon the recognition of many different attackers^41^, and these miRNAs may be implicated in the regulation of the common downstream signaling pathway, responding to different stresses.

### miRNAs expressed differentially in P15 and PC at 6 h and 48 h after BPH-feeding

As the Venn diagram shows (Fig. 6), at 6 h, there were 61 miRNAs recorded only in P15 and 25 miRNAs recorded only in PC. At 48 h, there were 17 miRNAs only in P15 and 29 miRNAs only in PC. There were almost three times as many miRNAs in the P15-6/P15-0 comparison group as in PC-6/PC-0, while just seven miRNAs appeared simultaneously in the two groups. In contrast, there were fewer P15-48/P15-0 miRNAs than PC-48/PC-0 miRNAs, while there were large numbers of common miRNAs. Comparing two time points for the same genotype, we found more P15-6/P15-0 miRNAs than P15-48/P15-0 miRNAs, but the number was fewer for PC-6/PC-0 than for PC-48/PC-0. These results indicate that at 6 h, the resistant plants had started to respond to the external stimuli, but the susceptible plants had only activated a small number of miRNAs, while in the later stage, the two genotypes responded in a more similar manner.

According to the annotation criteria for miRNAs in plants, the miRNAs from the same family probably have the same or similar mature sequences; normally, members from the same miRNA family have similar physiological functions^42^. We performed miRNA gene family classification and found several genotype-specific, differentially expressed miRNAs belonging to the same family (Table 3). We found 11 miRNA families that appeared in both genotypes, while P15 had more differentially expressed miRNAs than PC, and they were preferentially found in P15-6/P15-0. It is likely that in response to BPH attack, P15 was much wider and more rapid than PC. The miRNAs of the same family in the two different rice lines mostly exhibited a similar expression pattern, except for miR528 and miR2864. All the miRNAs were down-regulated after BPH attack, with the exceptions that miR528-3p in PC-48/PC-0 and miR2864.1 in P15-48/P15-0 were up-regulated. Nine of the 11 miRNA families have been reported to be responsive to biotic or abiotic stresses, except the miR1883 and miR2864 families^38, 39, 43^. Among 16 known members in miR167 family, we detected that six of them were down-regulated. Six miR444 members were detected, and all of them were found in the 6 h samples, indicating that miR444 members were involved in the early-stage response to BPH attack.

**Table 3:**
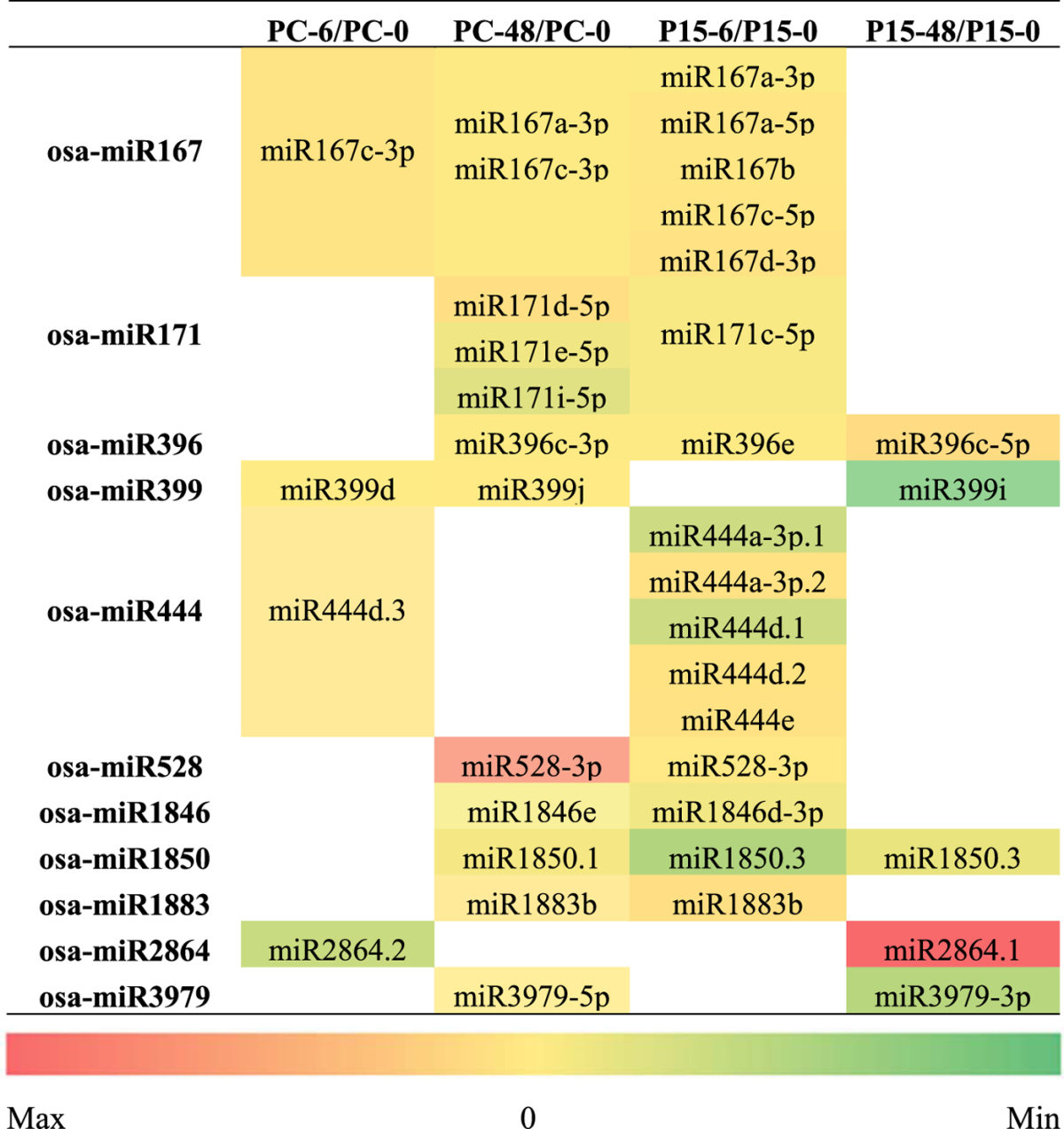
Differentially expressed miRNAs within the same families 6 h and 48 h after BPH feeding. The colors represent the fold-change values of the corresponding miRNAs.

Finally, 55 miRNAs were only found in P15 and 38 miRNAs were only found in PC (Table 4). In P15, we found miR156b-3p/c-3p/f-3p/g-3p/h-3p/l-3p, miR159a.2, miR160c-3p/e-5p/f-3p/f-5p, miR168a-3p, miR169a/b/c/e/h/i-3p/i-5p.1/i-5p.2/j/k/l/m/n /o/r-3p/r-5p, miR172d-5p, miR408-3p/-5p, miR530-5p, miR531a/b/c, miR535-5p, miR1429-3p, miR1431, miR1432-5p, miR1857-3p, miR2121a/b, miR5083, miR5143a, miR5156, miR5513 and miR6249a/b (Table 4). Most of these miRNA families reportedly played roles in regulating the plant responses to abiotic or biotic stress^36, 38, 44^. The target genes of miR156, miR159, miR169, and miR172 are categorized into different transcription factor families – SBP, MYB, CBF, bZIP – which further regulate gene expression and signal transduction and probably play roles in stress responses^35^. We also found that these miRNAs have differential expression in P15. It has been reported that osa-miR531 targets 25 members of the MAPK cascade gene family^29^. miR531a/b/c were down-regulated at 6 h in P15, so the MAPK cascade would be activated in P15. On the other hand, the 38 miRNAs differentially expressed only in PC represented eight families (miR166, miR395, miR812, miR827, miR1320, miR1862, miR3980 and miR6248) (Table 4). These have been reported to be regulated in plants under abiotic stress such as cold, drought, and H_2_O_2_^30, 36, 39, 45–47^. These findings indicate the complex network of regulatory relationships between miRNAs and BPH stress.

**Table 4:**
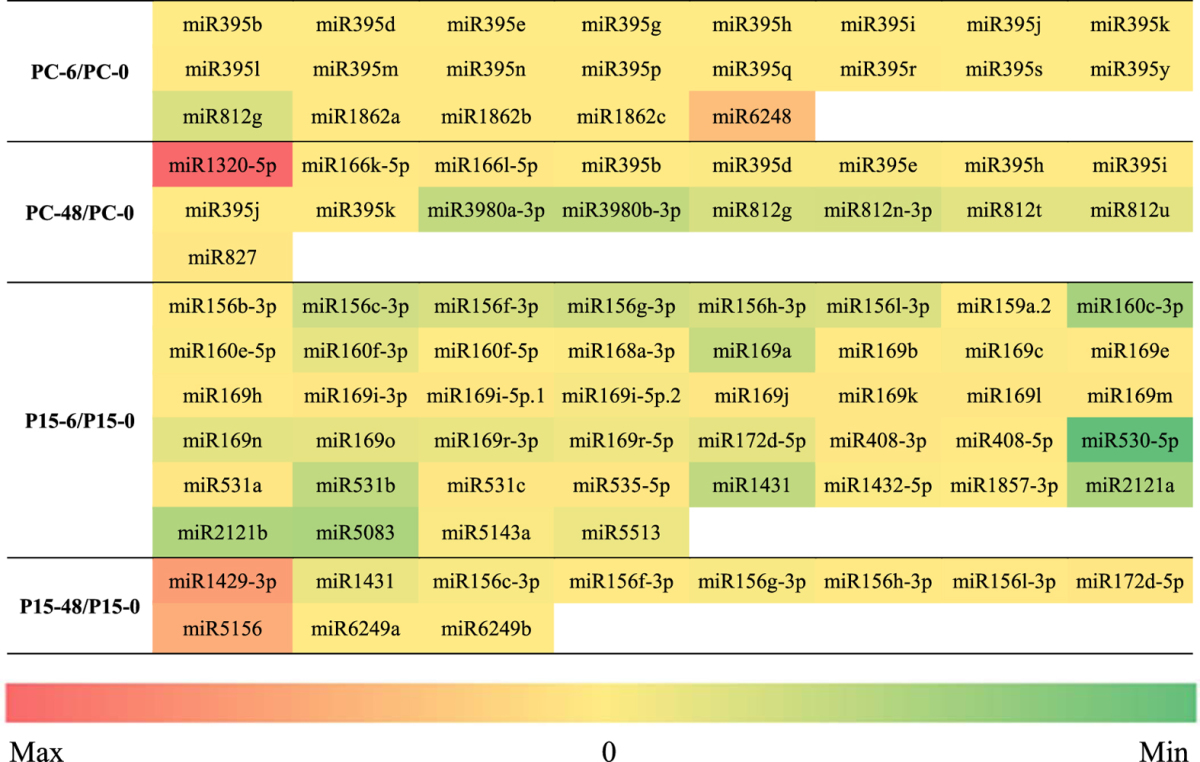
Differentially expressed miRNAs that belong to different families at 6 h and 48 h after BPH feeding. The colors represent the fold-change values of the corresponding miRNAs.

Based on the four comparison groups above, we predicted 1664 targets of 158 differentially expressed miRNAs. When the duplicated genes were removed, there were 472 genes remaining. To examine whether these genes were related to resistance, we carried out a GO analysis of them and identified the genes enriched in resistant GO terms with a P value < 0.05. We also checked their expression with the RNA-seq data to determine whether each of them was the opposite of their corresponding miRNA or not^32^. Eventually, we identified 94 targets that exhibited the opposite expression to 46 miRNAs and were also related to defense responses (Table S4).

### miRNA down-regulated the expression of a reporter construct that contains the target sequence

**Figure 8:**
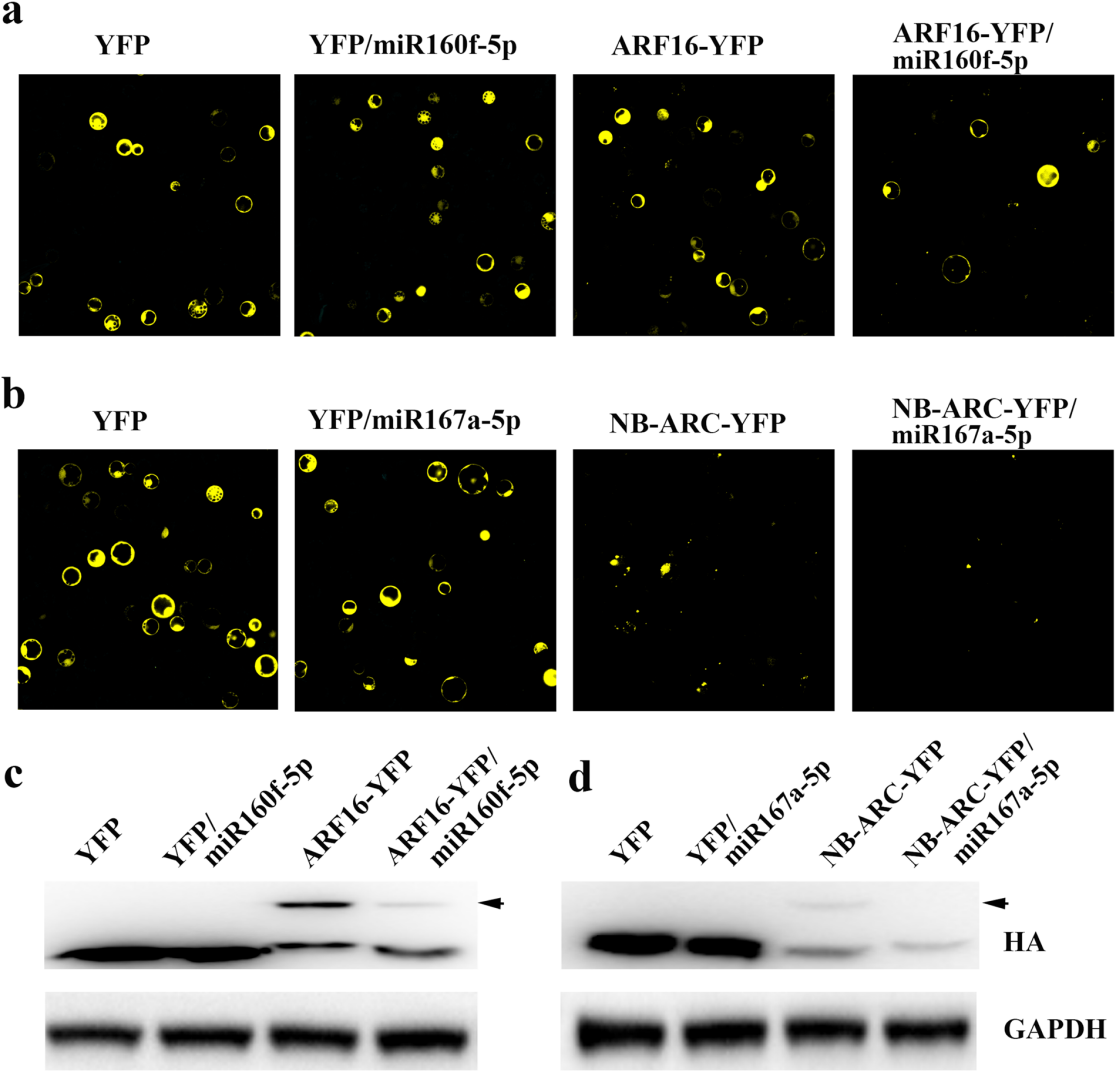
Target assay of effect of miRNAs on the predicted target gene expression in rice protoplasts. (**a**) Fluorescence micrographs of rice protoplasts transfected with YFP, YFP/miR160f-5p, ARF16-YFP and ARF16-YFP/miR160f-5p from left to right. The photographs were taken at 60 × magnification. (**b**) Fluorescence micrographs of rice protoplasts transfected with YFP, YFP/miR167a-5p, NB-ARC-YFP and NB-ARC-YFP/miR167a-5p from left to right. The photographs were taken at 60 × magnification. (**c**) Western blot analysis of ARF16 expression or black plasmid expression in rice protoplasts transfected with YFP, YFP/miR160f-5p, ARF16-YFP and ARF16-YFP/miR160f-5p from left to right using anti-HA and anti-GAPDH antibodies. ARF16 is indicated with arrow. (**d**) Western blot analysis of NB-ARC expression or black plasmid expression in rice protoplasts transfected with YFP, YFP/miR167a-5p, NB-ARC-YFP and NB-ARC-YFP/miR167a-5p from left to right using anti-HA and anti-GAPDH antibodies. NB-ARC is indicated with arrow. The original blots of **c** and **d** are shown in Fig. S5.

In theory, a miRNA could negatively regulate its target gene expression. We selected two miRNAs and their predicted target genes for validation in rice protoplasts: miR160f-5p and miR167a-5p, with target genes of auxin response factor 16 (*ARF16*/LOC_Os02g41800) and NBS-LRR disease resistance protein (*NB-ARC*/LOC_Os07g29820), respectively. Compared with the protoplasts just transfected with the target plasmid *ARF16-YFP*, the YFP signal of the protoplasts co-transfected with *ARF16-YFP* and miR160f-5p was reduced. However, the YFP signal was not affected by co-transfection with blank plasmid *YFP* and miR160f-5p (Fig. 8a). Similarly, the expression of the target gene *NB-ARC* was down-regulated by the co-expression of miR167a-5p (Fig. 8b). Western blotting confirmed the protein expression in protoplast cells in accordance with the YFP signal (Fig. 8c,d). Both protoplast fluorescence results and western blotting results show that miR160f-5p negatively regulated the expression of *ARF16* and miR167a-5p negatively regulated the expression of *NB-ARC* in rice cells.

## Discussion

Evidence is accumulating for a role of miRNAs in the plant responses to biotic stress^14–18^. In plant-insect interactions, aphid-induced small RNA expression has been studied in many plants^48^. This is the first report of the miRNA response to herbivore insect in rice. In this study, our work provides a detailed snapshot of the miRNA expression pattern in host rice after BPH attack, which helped us dissect the interaction between rice and BPH and also the mechanisms of *BPH15* resistance related to miRNAs. We compared the miRNA expression between the resistant (P15) and susceptible (PC) rice plants before and after BPH attack. A total of 464 known miRNAs and 183 potential novel miRNAs were identified, and among them 158 miRNAs were differentially regulated in rice plants infested by BPHs. The results indicate that BPH feeding re-programmed the miRNA expression in rice. A wide range of genes are predicted to be regulated by these miRNAs, but the GO analysis of the target genes showed that the terms were mainly distributed in several categories, including the response to stimulus, signal transduction, callose deposition in cell wall, and leaf senescence. Our results suggest that miRNAs were involved in different pathways to regulate the defense responses of rice to BPH.

After BPH feeding, 104 miRNAs were differentially expressed in resistant rice P15 and 80 miRNAs in susceptible rice PC. Among them, 26 miRNAs were common in the two rice lines (Fig. 6). This indicates that more miRNAs are regulated in resistant plants than in susceptible plants in response to BPH feeding. We found a number of miRNAs differentially expressed in P15 before and after BPH attack. The expression level of miR444d decreased in 6 h after BPH feeding. miR444d targets a calmodulin-binding protein^49^. Ca^2+^ is a second messenger and triggers physiological changes in the plant immunity to pathogens^50^. Also, the early rice defense response to BPH involves a Ca^2+^ influx^51–53^. The alteration of Ca^2+^ levels acts as a signal to rice plant cells to elicit defense responses, including the formation of reactive oxygen intermediates (ROIs) and callose synthesis^53^. A calmodulin-binding protein was identified as a potential target of miR444d in this study, which suggests a role for miR444d in calcium signaling in the resistance to BPH at the early stage. The mitogen-activated protein kinase (MAPK) signaling cascade consists of several distinct MAPKKK-MAPKK-MAPK modules receiving signals from various upstream receptors and sending them to downstream targets through sequential phosphorylation, leading to the activation of physiological processes^54^, and it has been proven to act as an important signal transduction component in microbe and insect elicitor perception^55^. miR531a/b/c-targeting MAPK cascade genes were down-regulated in P15 at 6 h after BPH feeding, which suggested the involvement of miR531a/b/c for activating the MAPK pathway. In maize miR168, targeting the MAPK was also inhibited under drought stress^56^. In addition, we also confirmed that miR167a-5p inhibited the expression of an NB-ARC protein. The *NB-ARC* gene family is an important class of *R* gene that responds to the effectors of pathogens and insects^57^. As miR167a-5p expression decreased in P15 at 6 h, it is likely that this miRNA participates in effector-triggered immunity (ETI) signaling to regulate the defense response to BPH attack.

Plant auxin regulates many important aspects of development as well as responses to environmental stresses^58^. ARF16 has been reported to repress the auxin signal^36^. In our study, miR160f-5p declined in P15 at 6 h, which could balance the relationship between the plant growth and stress response. We also validated the effect of miR160f-5p on ARF16 expression in rice protoplasts; the overexpression of miR160f-5p led to the reduction of ARF16 protein in rice cells.

Members of the miR399 family were down-regulated after BPH attack. A previous studies showed that miR399 increased in response to low-phosphate stress and targeted a gene encoding a putative ubiquitin conjugating enzyme (UCE)^59, 60^. The differences in the expression of the same miRNAs under abiotic and biotic stresses illustrates that miRNAs alter their expression patterns to address multiple stresses. Members of the miR156 family target the SBP-LIKE and MYBs/TCPs transcription factors. SBP-LIKE is involved in the floral transition and regulation of flowering and affects the phase transition from vegetative growth to reproductive growth^61^. TCP also directs the developmental processes of leaf morphogenesis^62^. miR156b-3p/c-3p/f-3p/g-3p/h-3p/l-3p were down-regulated in P15 when attacked by BPH, suggesting that miR156 may be involved in the adaption to BPH attack by modulating the plant morphological characteristics.

Additionally, we found that miR1846e and miR3979-3p were down-regulated by BPH attack, which was contrary to the case for pathogens^43^. This conflicting result highlights that these miRNAs are associated with resistance to pests and pathogens through different target genes that regulate different signal pathways. As regulators of stress, miRNAs exhibit unique behaviors under varying stress conditions, so more in-depth and detailed characterizations of stress-responsive miRNAs are needed in the future.

It was noted that the miRNA expression profiles significantly varied between the early stage (6 h) and late stage (48 h). During feeding on a rice plant, the BPH stylet transiently punctures the epidermis and then penetrates the plant cell walls. The insect subsequently salivates into the cells and ingests the phloem sap^51^. According to an electronic penetration graph (EPG) waveform recording, the phloem feeding generally appears 1–3 hours after the BPH settling on rice^51^. At the early stage of 6 h, the plant had not yet been damaged, so more miRNAs were regulated in P15 than in PC (Fig. 6). The results demonstrated that the resistant plant responds more rapidly to BPH than the susceptible plant from the site of miRNA expression. As the feeding continued, the plant was damaged at the late stage, and the miRNA profile should thus be different from that in the early stage. miRNAs such as miR398, which keeps the balance the mount of ROS productivity and modulates hormone signaling cascades, and miR395, which participates in modulating plant growth and development, were regulated in both rice lines at the late stage (Fig. 7)^59, 63^. The regulation pattern of miRNA is consistent with the gene expression, hormones and metabolites at the early and late stages^21, 32, 64^.

Rice and BPH form a model system for the dissection of the mechanism of interaction between a crop and insect pest^65^. Zha *et al*. found 26 miRNAs differentially expressed in BPHs feeding on resistant rice and susceptible rice^66^. The target genes of these miRNAs belonged to pathways including metabolism, the circulatory system, neurodegenerative diseases, the immune system, and energy metabolism. Here, we presented miRNA expression profiles in rice plants before and after BPH attack. A number of miRNAs were differentially regulated in responding to BPH attack. These miRNAs regulate different pathways and contribute to the basal defense and specific resistance of rice against BPH. These results suggest mutual regulatory relationships between BPH and rice, by which BPH miRNAs would be regulated to adapt to rice resistance, and rice miRNAs would be regulated to resist BPH. Our results of miRNA regulation in rice infested by BPH provide valuable data to understand the plant’s defense system against herbivores.

## Materials and Methods

### Plant and insect materials

We used the *BPH15* introgression line (P15) and susceptible recipient line 9311 (PC)^25^. Two types of seed were sown in pots (20 cm in diameter and 20 cm in height), with 30 plants per pot in a greenhouse, which was controlled to have 30 ± 2 °C/14 h light (07:00–21:00) and 28 ± 2 °C/10 h dark (21:00–07:00) cycles. The rice was allowed to produce two leaves (about two weeks after sowing) before being used in the experiments.

The BPH population (biotype 1) was reared on TN1 under the same temperature and light regime described above. We used second or third instar nymphs of BPH for infestation experiments.

### BPH infestation and sample collection

We employed the endpoint method for BPH treatments and sample collection^64^. All time points of treatments began at different times and stopped at the same time. BPHs were introduced to the rice plants at a density of eight insects per seedling. Stem samples were collected 0, 6 and 48 h after BPH infestation. The samples are referred to as P15-0, P15-6, P15-48 for the *BPH15* introgression line and PC-0, PC-6, PC-48 for the susceptible recipient line, the number representing the time after infestation. The stems of 30 rice plants of each treatment were quickly cut as a combined sample, and immersed in liquid nitrogen, then stored at −80 °C.

### Small RNA library preparation and sequencing

Total RNA was isolated using a RNAiso Plus kit (TaKaRa) according to the manufacturer’s instructions. The construction of the six sRNA libraries consisted of the following steps: (1) polyacrylamide gel electrophoresis (PAGE) purification of the RNA bands and RNA molecules in the size range 18–30 nt enriched; (2) ligation of the 5p adapters to the RNA; (3) ligation of the 3p adapters to the RNA; (4) RT-PCR amplification to generate cDNA libraries; (5) the libraries were used for single-end 100 × 2 sequencing using Illumina HiSeq. 2000. The sequencing data have been submitted to the NCBI’s GEO database, the accession number is GSE92549.

### Small RNA analysis and miRNAs prediction

Raw reads of the six libraries were filtered to remove low quality reads, poly A, incorrect adaptors and sequences shorter than 18 nt. The clean reads were compared to GenBank (release 209.0) and Rfam (release 11.0) to move other types of RNA (rRNA, snRNA, snoRNA, tRNA). Also the clean tags were aligned with rice genome. Those mapped to exons or introns were removed, and the tags mapped to repeat sequences were also removed. The rest of the unique sequences were used to search against the miRBase database (release 21, http://www.mirbase.org/) for known rice miRNA identification.

Unidentified sequences that did not match any of the above databases were further analyzed to find potential novel miRNAs. We first compared sequences to selected plant precursors (with the exclusion of specific species) in miRBase, and the mapped pre-miRNAs were then aligned against the specific species genomes to determine their genomic locations. Then the remaining unmapped sequences were compared to the genome sequence of rice with no mismatch and related to the genome position to find possible novel miRNAs on the basis of secondary structure prediction using Mireap software. The criteria for secondary structure prediction were: (1) Minimal miRNA sequence length is 18 bp; (2) Maximal miRNA sequence length is 25 bp; (3) Minimal miRNA reference sequence length is 20 bp; (4) Maximal miRNA reference sequence length is 23 bp; (5) Maximal copy number of miRNAs on reference is 20; (6) Maximal free energy allowed for a miRNA precursor is -18 kcal/mol; (7) Maximal space between miRNA and miRNA* is 300 bp; (8) Minimal space between miRNA and miRNA* is 16 bp; (9) Maximal bulge of miRNA and miRNA* is 4 bp; (10) Maximal asymmetry of miRNA/miRNA* duplex is 4 bp; (11) Flank sequence length of miRNA precursor is 20 bp.

### Differential expression analysis of miRNAs

Before comparing different groups, the frequency of miRNA counts was normalized as transcripts per million (TPM). The P-value of differential expression was calculated using Bioconductor edgeR package^67^. We used the absolute value of log_2_FC ≥ 1 and P < 0.05 as the threshold to judge the significance of each miRNA expression difference.

### Target gene prediction and functional annotation

Target genes of miRNAs were predicted using patmatch software. Gene Ontology (GO) (http://www.geneontology.org/) was used to further identify the functions of target genes.

### Analysis of miRNAs by stem-loop RT-PCR and real-time RT-PCR

Total RNA was isolated using a RNAiso Plus kit (TaKaRa) according to the manufacturer’s instructions. We used 2 ug total RNA to synthesize the first strand cDNA using a PrimeScript RT reagent Kit with gDNA Eraser (TaKaRa Lot# AK2802). Primers used in the stem-loop RT-PCR are listed in Table S5. The cDNA was amplified by real-time RT-PCR using the SYBR green supermix (Bio-Rad) and CFX96 real-time system following the manufacturer’s instructions. Primers used in real-time RT-PCR are also listed in Table S5. Three biological replicates were performed for each experiment. Normalized expression levels were calculated using the 2^−ΔΔC (t)^ method with U6 as the internal reference gene.

### Preparation and transformation of rice protoplast and western blotting assay

In order to verify predicted target genes. We constructed two kinds of plasmids, one encoded pri-miRNA (miR160f-5p, miR167a-5p), and the other was an approximately 300 bp DNA fragment of target mRNA (*ARF16*, *NB-ARC*) containing a sequence that is perfectly complementary to the mature form of miRNA followed by report genes of YFP and HA tags. The blank reporter plasmid (*YFP-HA*) was used as the control. Rice protoplasts were transiently transfected with these plasmids, and we recorded the number of fluorescing cells, and determined the amount of protein expression by western blotting. Thus, we were able to investigate the effect of miRNA on the expression of target genes. The detailed procedure was as follow.

Rice protoplasts isolated from 10-day-old wild-type plants stem were cotransfected with vectors expressing miRNA and the target gene using previously described procedures^68^. Briefly, the stems of about 100 seedlings were cut into 0.5 mm strips and immersed in Mannitol (0.6 M) for 10 min, then the tissue was transferred to 10 mL enzyme solution (1.5% Cellulase R-10, 0.75% Macerozyme R-10, 0.6 M mannitol, 10 mM MES, pH 5.7, 10 mM CaCl_2_, and 0.1% BSA) and shaken in the dark at 28 °C for 4 h to 5 h, then 10 ml W5 solution (154 mM NaCl, 125 mM CaCl_2_, 5 mM KCl, and 2 mM MES, pH 5.7) was added, before being filtered to remove tissue, and centrifugated at 1500 rpm for 3 min to collect the protoplasts. 10 ug plasmid DNA was used to transfect every 100 µL (2 × 10^6^ cells) of rice protoplasts mediated by PEG (40% PEG4000, 0.1 M CaCl_2_, 0.2 M Mannitol). Transfected protoplasts were incubated in the dark at 28 °C for 16 h for protein expression. Finally, the protoplasts were scanned and imaged using a confocal microscope (FV10-ASW; Olympus). Primers used in this experiment are listed in Table S5.

To perform western blotting, the transfected protoplasts were collected by centrifugation at 1500 rpm for 3 min, the supernatant discarded, and protein extraction solution added (0.1 M Tris-HCl, pH 7.5, 5 mM MgCl_2_, 1 mM EDTA, 0.5% Triton 100 2 mM DTT, 1/1000 PMSF), flip mixed for 1 h at 4 °C, then centrifuged at 13200 rpm for 10 min to collect the supernatant, which was then mixed with 4 × SDS sample buffer (250 mM Tris-HCl, pH 6.8, 40% glycerol, 6% SDS, 20% β-mercaptoethanol, and 0.04% bromphenol blue) and boiled for 10 min, the extract was then analyzed by 10% (w/v) SDS-PAGE, after electrophoresis, using HA or GAPDH antibody to detect the expression of the target gene.

### Statistical analysis

Statistical analysis of differential expressed miRNAs was calculated using Bioconductor edgeR package with the condition that the ratio was greater than 2 and P < 0.05. The statistical analyses of all the real-time RT-PCR data were performed using One-way ANOVA in SPSS 7 for Windows version 16.0 (SPSS Inc., USA).

### Data availability

The datasets generated and analyzed in this study are available in the NCBI’ GEO repository, and are accessible through GEO series accession number GSE92549 (https://www.ncbi.nlm.nih.gov/geo/query/acc.cgi?acc=GSE92549).

## Electronic supplementary material

Supplementary information
