# Supplementary material for: Identification and analysis of brown planthopper-responsive microRNAs in resistant and susceptible rice plants
Source: Sci Rep. 2017 Aug 18;7:8712. doi: 10.1038/s41598-017-09143-y (PMC5562839; doi:10.1038/s41598-017-09143-y)
Supplement: Supplementary file 1 — Supplementary information [file 41598_2017_9143_MOESM1_ESM.pdf]

Supplementary Information

Title: Identification and analysis of brown planthopper-responsive microRNAs in resistant and susceptible rice plants

Authors : Yan Wu[1], Wentang Lv[1,2], Liang Hu[1], Weiwei Rao[1], Ya Zeng[1], Lili Zhu[1], Yuqing He[3], Guangcun He[1,*]

Affiliation:

1.National Key Laboratory of Hybrid Rice, College of Life Sciences, Wuhan University, Wuhan 430072, China

2.Shandong Rice Research Institute, Shandong Academy of Agricultural Sciences, Jinan 250100, China

3.National Key Laboratory of Crop Genetic Improvement, Huazhong Agricultural University, Wuhan 430070, China

[*]**Corresponding author:**

National Key Laboratory of Hybrid Rice, College of Life Sciences, Wuhan University, Wuhan 430072, China. Phone: +86-27-68752384 Fax: +86-27-68752327 Email: gche@whu.edu.cn

Supplemental Materials

Supplemental Figure S1. The preference distribution for uridine at the 5' end position of different lengths (nt) of known miRNAs.

Supplemental Figure S2. The preference distribution for uridine at the 5' end position of different lengths (nt) of novel miRNAs compared to known miRNAs of other selected plants in miRBase.

Supplemental Figure S3. The preference distribution for uridine at the 5' end position of different lengths (nt) of novel miRNAs identified by secondary structure prediction.

Supplemental Figure S4. Predicted secondary structures of the seven novel miRNAs.

Supplemental Figure S5. Original blots showing cropped areas for making Fig. 8c and 8d.

Supplemental Table S1. Novel miRNAs compared to the known miRNAs of other selected plants in miRBase and their total counts ≥ 100.

Supplemental Table S2. Novel miRNAs identified by secondary structure prediction and their total counts ≥ 100.

Supplemental Table S3. The target prediction of the differently expressed miRNAs in P15-0/PC-0.

Supplemental Table S4. The GO annotation of 94 resistance-related targets exhibiting the opposite expression to 46 differently expressed miRNAs.

Supplemental Table S5. Primers used for qRT-PCR and transformation of rice protoplasts.

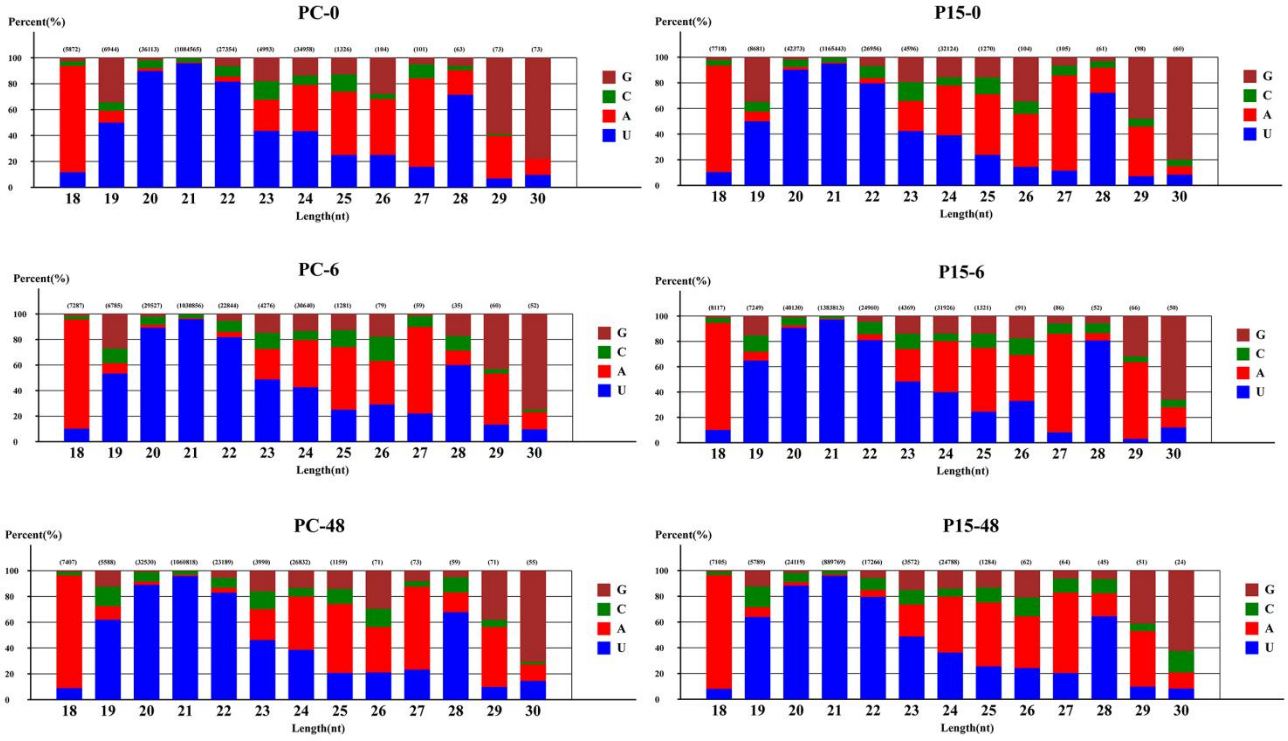

Supplemental Figure S1. The preference distribution for uridine at the 5' end position of different lengths (nt) of known miRNAs.

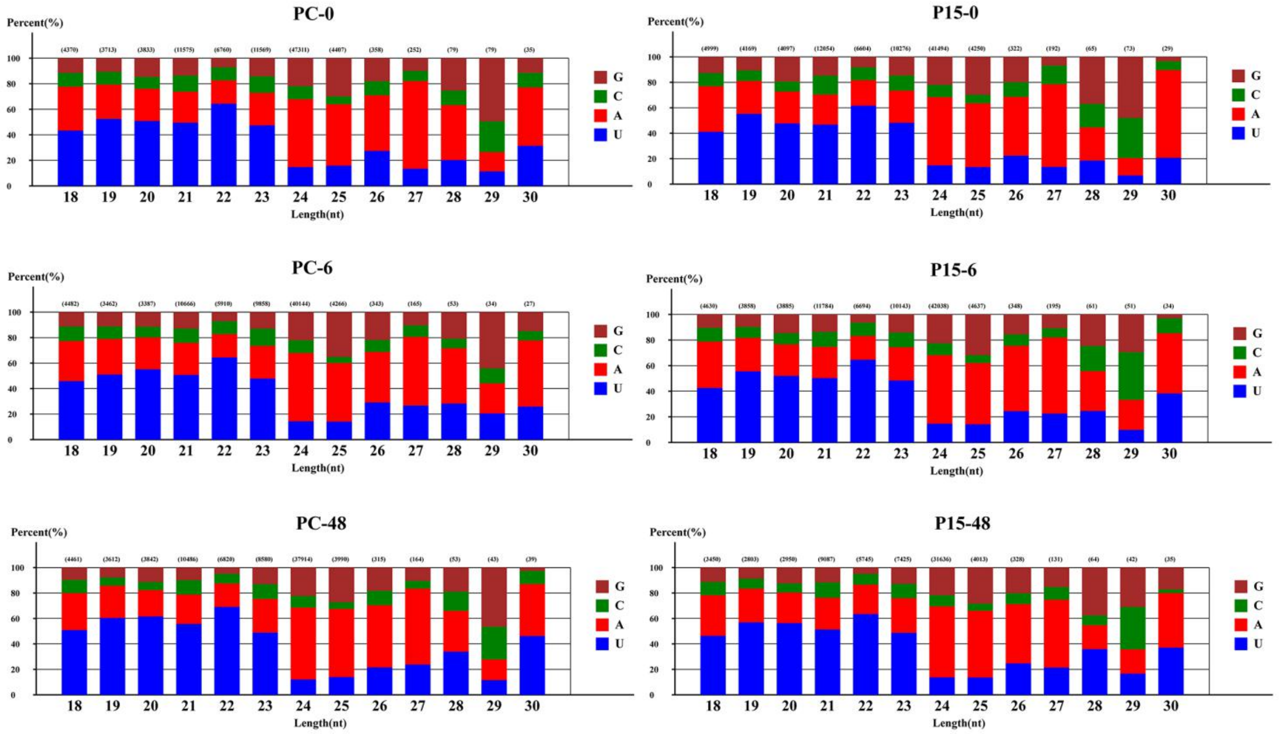

Supplemental Figure S2. The preference distribution for uridine at the 5' end position of different lengths (nt) of novel miRNAs compared to known miRNAs of other selected plants in miRBase.

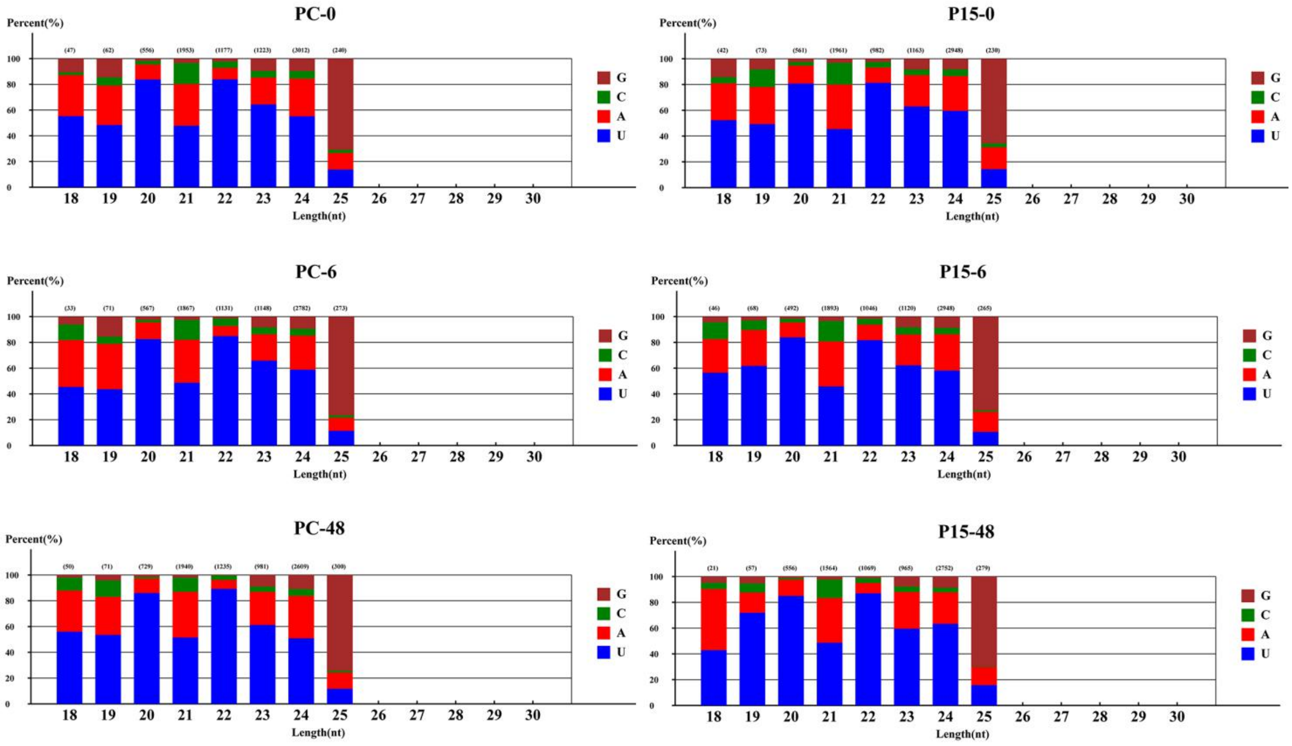

Supplemental Figure S3. The preference distribution for uridine at the 5' end position of different lengths (nt) of novel miRNAs identified by secondary structure prediction.

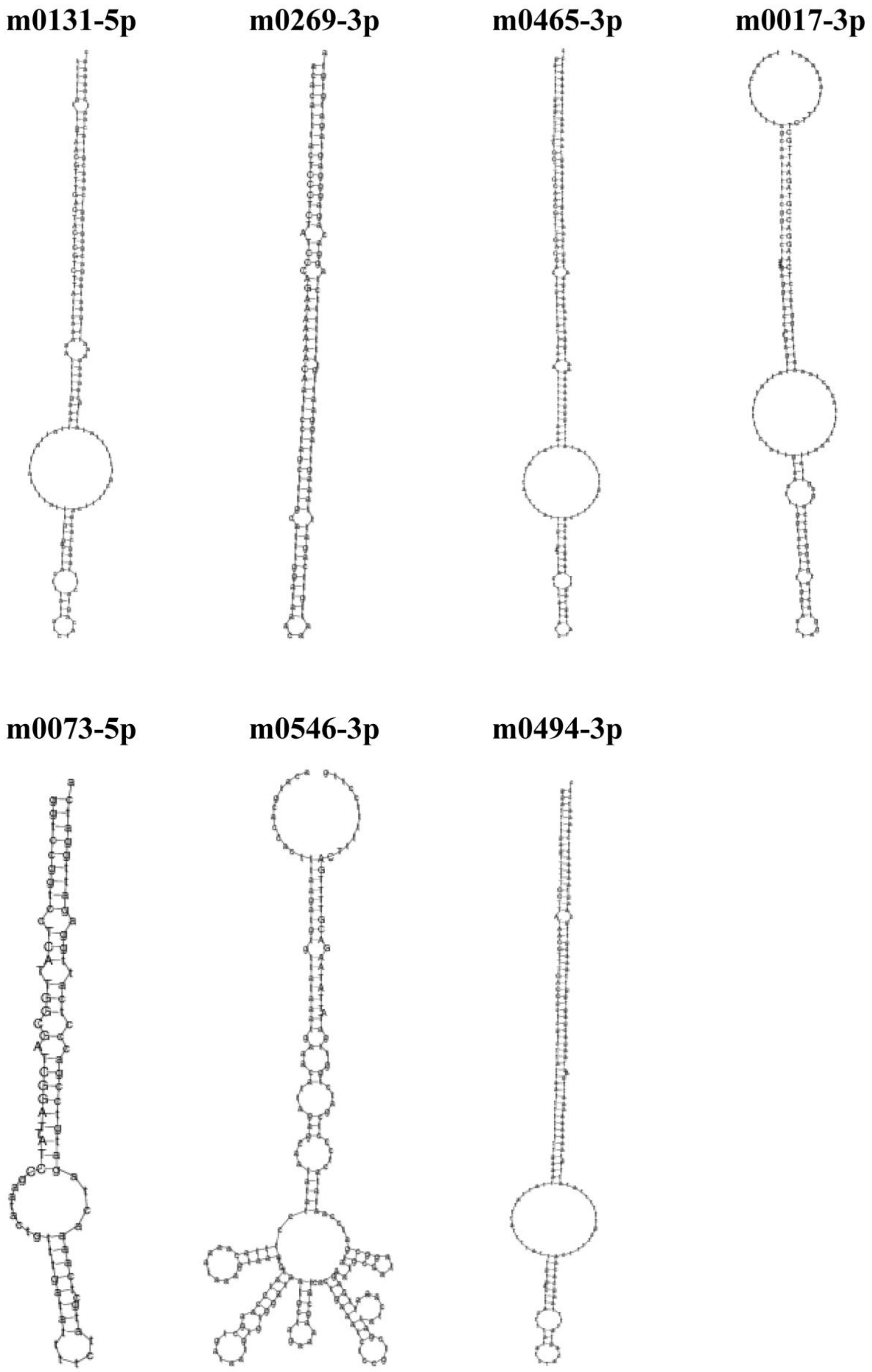

Supplemental Figure S4. Predicted secondary structures of the seven novel miRNAs.

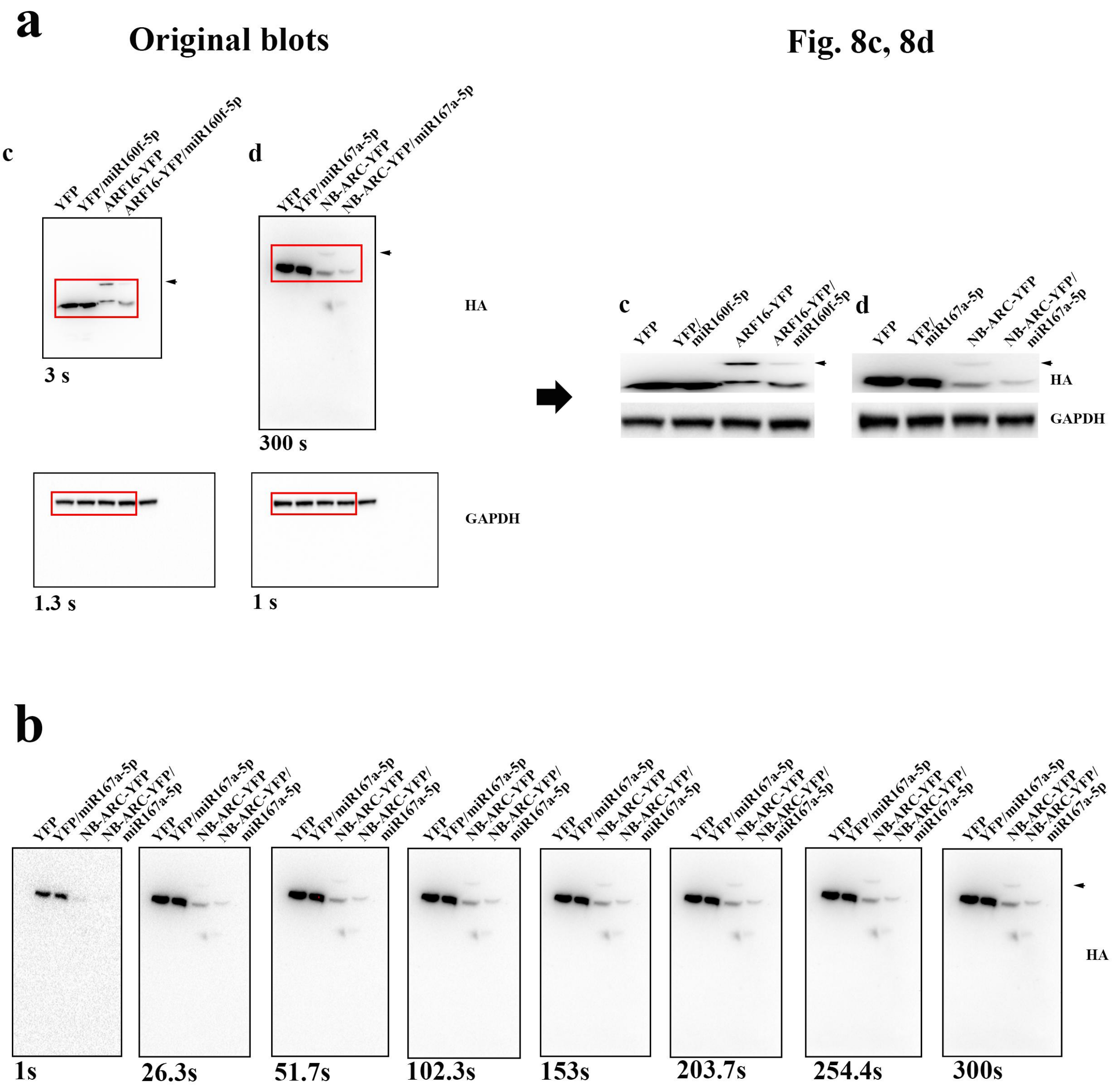

Supplemental Figure S5. Original blots showing cropped areas for making Fig. 8c and 8d. a. Red boxes in the original blots indicate cropped areas for the construction of Fig. 8c and 8d. The numbers below the original blots indicate the exposure time (s). **b.** The western detected by anti-HA antibody in Fig. 8d exposed at different time points (s).

Supplemental Table S1. Novel miRNAs compared to the known miRNAs of other selected plants in miRBase and their total counts ⩾ 100.

ID	Length	Seq	Total	PC-0_ count	PC-6_ count	PC-48_ count	P15-0_ count	P15-6_ count	P15-48_ count	PC-0_TPM	PC-6_TPM	PC-48_ TPM	P15-0_TPM	P15-6_TPM	P15-48_ TPM
MIR166-y	21	GCTCGGACCAGGCTTCATTCC	17105	2883	2731	2670	2899	3698	2224	2145.2297	2169.6403	2080.5816	2032.8766	2249.3028	2061.7104
MIR812-y	24	AAGACGGACGGTCAAACATTGGAC	13388	2675	2153	1979	2411	2553	1617	1990.4577	1710.4488	1542.124	1690.6746	1552.8583	1499.0044
MIR894-x	19	TTTCACGTCGGGTTCACCA	8362	1386	878	1313	2093	1663	1029	1031.3175	697.5262	1023.1474	1467.6822	1011.5172	953.9119
MIR159-y	23	TTTGGATTGAAGGGAGCTCTGTT	3397	633	470	490	602	758	444	471.013	373.391	381.8296	422.1427	461.0523	411.6005
MIR5073-x	25	GTTTGGTGAATCGGAAACTTTTTTC	2961	511	725	289	391	642	403	380.2332	575.9755	225.2015	274.1824	390.4955	373.5923
MIR396-x	22	TCCACAGGCTTTCTTGAACTTC	2676	428	342	444	528	543	391	318.4732	271.7016	345.9844	370.2514	330.2789	362.468
MIR5139-x	18	AACCTGGCTCTGATACCA	2491	429	276	348	650	527	261	319.2173	219.2679	271.1769	455.8019	320.5469	241.9543
MIR1511-y	18	AACCTGGCTCTGATACCA	2297	387	255	326	599	493	237	287.9653	202.5845	254.0336	420.039	299.8665	219.7057
MIR8155-y	18	AACCTGGCTCTGATACCA	2280	382	253	322	599	487	237	284.2448	200.9956	250.9166	420.039	296.217	219.7057
MIR5168-y	23	TCGGACCAGGCTTCAATCCCTTC	1813	335	299	248	346	383	202	249.2723	237.5403	193.2525	242.6269	232.9592	187.2597
MIR529-x	21	AGAAGAGAGAGAGTACAGCCA	1769	220	279	277	313	318	362	163.7012	221.6513	215.8506	219.4862	193.423	335.5842
MIR8175-y	18	TCCCCGGCAACGGCGCCA	1585	204	236	312	375	222	236	151.7957	187.49	243.1241	262.9627	135.0312	218.7786
MIR5145-x	24	CACCTGTTTGGATCCTTGAGAGCT	1215	258	193	159	227	267	111	191.9769	153.3287	123.8998	159.1801	162.4023	102.9001
MIR1862-y	24	TACGAGATTGGTTTATTTTGAGAC	1119	210	187	170	185	210	157	156.2602	148.562	132.4715	129.7282	127.7322	145.5434
MIR812-x	20	TTCACGTTCAACGTTTGACT	1068	157	170	221	192	180	148	116.8231	135.0563	172.2129	134.6369	109.4847	137.2002
MIR827-y	21	AGATGACCATCAGCAAACAAG	1060	216	162	91	226	180	185	160.7248	128.7007	70.9112	158.4788	109.4847	171.5002
MIR166-z	21	TATCGGACCAGGCTTCATTCC	978	175	154	152	172	189	136	130.2169	122.3451	118.4451	120.6122	114.959	126.0758
MIR477-x	18	TCTCCCTCAAAGGTCTCT	727	135	189	63	138	128	74	100.453	150.1509	49.0924	96.7703	77.8558	68.6001
MIR156-x	22	CTTGACAGAAGAGAGTGAGCAC	710	138	103	110	150	121	88	102.6853	81.8282	85.7168	105.1851	73.5981	81.5785
MIR169-y	22	GGCAAGTCTGTCCTTGGCTACT	708	167	116	76	165	94	90	124.2641	92.1561	59.2225	115.7036	57.1754	83.4325
MIR168-x	23	TCGCTTGGTGCAGATCGGGACCT	680	106	84	96	157	136	101	78.8742	66.7337	74.8074	110.0937	82.7218	93.6298
MIR5825-y	24	AAACATCTTATAACCTGAAACGGA	612	102	119	119	92	85	95	75.8978	94.5394	92.73	64.5135	51.7011	88.0677
MIR814-x	24	ACACATCCTAGTACAACGAATCTG	591	106	87	109	102	107	80	78.8742	69.1171	84.9376	71.5258	65.0826	74.1622
MIR1133-x	21	TTATACTCCCTCCGTCCCAAA	586	111	93	76	114	106	86	82.5947	73.8838	59.2225	79.9406	64.4743	79.7244
MIR160-x	19	TGCCTGGCTCCCTGTATTT	547	65	91	83	110	85	113	48.3663	72.2949	64.6773	77.1357	51.7011	104.7542
MIR5054-y	18	TCCCCACGGACGGCGCCA	484	67	79	94	108	76	60	49.8545	62.7615	73.2489	75.7332	46.2269	55.6217
MIR393-x	23	TCCAAAGGGATCGCATTGATTTT	473	80	67	78	97	75	76	59.5277	53.2281	60.781	68.0197	45.6186	70.4541
MIR6225-x	21	TTAGACTCAAAAGATTCGTCT	415	85	69	79	59	70	53	63.2482	54.817	61.5603	41.3728	42.5774	49.1325
MIR818-y	24	AAATCCCTTATATTGTAGGACGGA	414	77	57	87	71	58	64	57.2954	45.2836	67.7942	49.7876	35.2784	59.3298
MIR9774-y	22	AGCAAGATATTGGGTATTTCTT	390	52	54	91	60	40	93	38.693	42.9002	70.9112	42.074	24.3299	86.2136
MIR1128-x	21	TTATACTCCCTCCGTCCCAAA	371	88	55	49	72	71	36	65.4805	43.6947	38.183	50.4888	43.1856	33.373
MIR319-y	23	CTTGGACTGAAGGGTGCTCCCTT	366	56	57	57	58	85	53	41.6694	45.2836	44.4169	40.6716	51.7011	49.1325
MIR5150-y	24	AGAAGCTGCAGCTGCTAGAAGCTC	356	64	72	65	61	48	46	47.6222	57.2003	50.6509	42.7753	29.1959	42.6433
MIR5160-y	24	GCGAGATCGACGGTATATTTCTGT	352	77	69	43	43	66	54	57.2954	54.817	33.5075	30.1531	40.1444	50.0595
MIR444-y	21	TGTTGTCTCAAGCTTGCTGTT	327	63	44	58	66	50	46	46.8781	34.9558	45.1962	46.2814	30.4124	42.6433
MIR166-x	23	GGAATGTTGTCTGGTTCAAGGAT	320	55	40	51	69	64	41	40.9253	31.778	39.7414	48.3851	38.9279	38.0082
MIR5827-x	21	TTTGTTGCACTTTGGACCACC	316	60	55	41	55	70	35	44.6458	43.6947	31.949	38.5679	42.5774	32.446
MIR5794-y	23	TGAGGAATCACTAGTAGTCGTTT	315	53	41	61	44	64	52	39.4371	32.5724	47.5339	30.8543	38.9279	48.2055
MIR156-y	23	GCTCACTGCTCTGTCTGTCATCC	302	75	47	45	61	39	35	55.8072	37.3391	35.066	42.7753	23.7217	32.446
MIR1425-x	23	TAGGATTCAATCCTTGCTGCTTT	297	60	49	47	37	43	61	44.6458	38.928	36.6245	25.9456	26.1547	56.5487
MIR159-x	19	AGCTCCCTTCGATCCAATT	296	57	56	49	56	35	43	42.4135	44.4891	38.183	39.2691	21.2887	39.8622
MIR169-x	23	TAGCCAAGGATGATTTGCCTGTT	266	47	45	55	46	39	34	34.9725	35.7502	42.8584	32.2568	23.7217	31.519
MIR7758-x	22	CCACTACCGTCAGTTGACCGTG	263	46	41	39	49	54	34	34.2284	32.5724	30.3905	34.3605	32.8454	31.519
MIR6225-y	21	CGAGACGAATCTTTTAAGCCT	255	53	36	27	53	52	34	39.4371	28.6002	21.0396	37.1654	31.6289	31.519
MIR168-y	21	CCCGCCTTGCACCAAGTAATT	248	45	41	31	61	31	39	33.4843	32.5724	24.1566	42.7753	18.8557	36.1541
MIR5816-x	24	GCTAGGAGTGTTTGTAGGAACGCC	241	39	47	37	42	37	39	29.0198	37.3391	28.832	29.4518	22.5052	36.1541
MIR165-y	20	TCGGACCAGGCTTCTTCCCC	238	40	27	49	48	52	22	29.7639	21.4501	38.183	33.6592	31.6289	20.3946
MIR171-y	21	TTGAGCCGTGCCAATATCACT	237	42	40	28	52	39	36	31.252	31.778	21.8188	36.4642	23.7217	33.373
MIR5801-x	20	TCGTTTCCGATCGTTGGATC	230	34	37	29	49	53	28	25.2993	29.3946	22.5981	34.3605	32.2372	25.9568
MIR5788-x	21	CGGATGTGACATATTCTAGTA	228	48	35	40	42	32	31	35.7166	27.8057	31.1698	29.4518	19.464	28.7379
MIR162-y	23	TCGATAAACCTCTGCATCCAGTC	212	46	44	24	35	35	28	34.2284	34.9558	18.7019	24.5432	21.2887	25.9568
MIR1441-y	22	TCGGATGTCAGAAAAGGTTTTC	209	43	20	42	40	42	22	31.9961	15.889	32.7283	28.0494	25.5464	20.3946
MIR5148-y	24	TGAGGGGTAGAAACGTCATATCGT	208	44	36	25	42	37	24	32.7402	28.6002	19.4811	29.4518	22.5052	22.2487
MIR167-x	20	TGAAGCTGCCAGCATGATTC	205	36	35	31	36	39	28	26.7875	27.8057	24.1566	25.2444	23.7217	25.9568
MIR2121-y	24	GAAAACGGAGCGGTCTATTAGAGC	205	42	41	22	36	35	29	31.252	32.5724	17.1434	25.2444	21.2887	26.8838
MIR1423-x	24	GCAACTACACGTTGGGCGCTCGAA	195	41	36	25	41	32	20	30.5079	28.6002	19.4811	28.7506	19.464	18.5406
MIR5072-x	18	TCCCCAGTGGAGTCGCCA	189	26	29	30	40	24	40	19.3465	23.039	23.3773	28.0494	14.598	37.0811
MIR5799-x	24	AGACGAATGGTCAAACGTTGGACA	186	32	40	22	40	28	24	23.8111	31.778	17.1434	28.0494	17.031	22.2487
MIR5808-x	20	TCGTTTCCGATCGTTGGATC	184	25	33	28	36	37	25	18.6024	26.2168	21.8188	25.2444	22.5052	23.1757
MIR820-x	23	TCGGCCTCGTGGATGGACCAGGT	177	26	31	29	29	32	30	19.3465	24.6279	22.5981	20.3358	19.464	27.8108
MIR3981-y	18	TTAGGATACGTCTCATCT	177	27	29	54	23	23	21	20.0906	23.039	42.0792	16.1284	13.9897	19.4676
MIR1863-y	26	TAGAAACTTGGCTGATGCATTACTCA	175	34	28	24	25	41	23	25.2993	22.2446	18.7019	17.5308	24.9382	21.3216
MIR396-y	21	ATAGTTCAAGAAAGTCCTTTT	166	24	25	18	47	29	23	17.8583	19.8612	14.0264	32.958	17.6392	21.3216
MIR1436-y	23	TACATTATGAAACGGAGGGAGTA	165	37	43	24	18	23	20	27.5316	34.1613	18.7019	12.6222	13.9897	18.5406
MIR2120-y	24	AATAGATCTTTAGTCCCGGTTGGT	163	28	28	29	35	26	17	20.8347	22.2446	22.5981	24.5432	15.8145	15.7595
MIR167-y	21	AGGTCATGCTGAAGTTTCATC	163	27	29	21	34	30	22	20.0906	23.039	16.3641	23.8419	18.2475	20.3946

MIR2880-x	24	GACGATATCCCGTTCGGACAAGAT	150	21	31	24	26	25	23	15.626	24.6279	18.7019	18.2321	15.2062	21.3216
MIR393-y	19	TCAGTGCAATCCCTTTGTT	145	21	24	32	27	25	16	15.626	19.0668	24.9358	18.9333	15.2062	14.8324
MIR390-x	21	AGCTCAGGATAGATAGCGCCT	142	33	14	20	28	21	26	24.5552	11.1223	15.5849	19.6345	12.7732	24.1027
MIR1883-x	25	CCTGTGACGGGCCGAGAATGGAACT	138	20	26	20	21	27	24	14.8819	20.6557	15.5849	14.7259	16.4227	22.2487
MIR2877-y	24	TTGCATCCTCTGCACATTGGGCCA	129	19	16	20	19	32	23	14.1378	12.7112	15.5849	13.3234	19.464	21.3216
MIR1120-y	23	TTGTTATATTCTGAGACGGAGGG	129	20	25	14	28	22	20	14.8819	19.8612	10.9094	19.6345	13.3815	18.5406
MIR6478-x	20	CCGACCTTAACTCAGTTGGT	129	19	18	24	30	20	18	14.1378	14.3001	18.7019	21.037	12.165	16.6865
MIR394-x	22	TTGGCATTCTGTCCACCTCTCT	128	24	19	17	15	27	26	17.8583	15.0945	13.2471	10.5185	16.4227	24.1027
MIR9777-y	22	GTAGCAAATATATCTGAGCACA	126	31	14	17	37	15	12	23.067	11.1223	13.2471	25.9456	9.1237	11.1243
MIR1876-y	24	ATAAATGGGTTTGTGGGCTGGCCC	123	17	21	12	25	25	23	12.6496	16.6834	9.3509	17.5308	15.2062	21.3216
MIR2871-y	23	TATTTTAGTTTCTATGGTCACTC	121	20	21	19	18	14	29	14.8819	16.6834	14.8056	12.6222	8.5155	26.8838
MIR399-y	21	TGCCAGAGGAGAACTGCACTG	120	28	15	9	31	23	14	20.8347	11.9167	7.0132	21.7382	13.9897	12.9784
MIR2871-x	21	TGACCGTAGAAACTAGCATTT	118	15	26	17	16	22	22	11.1614	20.6557	13.2471	11.2197	13.3815	20.3946
MIR5568-x	18	TTATAAGACGTTTTGACT	114	14	21	23	20	22	14	10.4173	16.6834	17.9226	14.0247	13.3815	12.9784
MIR2873-y	18	TGAAGTTGAGTTTGGACT	113	26	17	16	19	22	13	19.3465	13.5056	12.4679	13.3234	13.3815	12.0514
MIR1846-x	23	TCCCACCGAGCAGCCGGATCTTT	112	22	16	10	23	26	15	16.3701	12.7112	7.7924	16.1284	15.8145	13.9054
MIR5205-x	24	CTTATAATTTGGGACGGAGGGAGT	110	21	19	20	17	19	14	15.626	15.0945	15.5849	11.921	11.5567	12.9784
MIR1423-y	24	GAGCGCCCGAGCGGTAGTTGTCTG	105	22	19	9	22	15	18	16.3701	15.0945	7.0132	15.4271	9.1237	16.6865
MIR1873-x	21	TCAACATGGTATCAGAACTGT	103	18	16	25	16	20	8	13.3937	12.7112	19.4811	11.2197	12.165	7.4162

Supplemental Table S2. Novel miRNAs identified by secondary structure prediction and their total counts ≥ 100.

ID	Length	Seq	Total	PC-0_ count	PC-6_ count	PC-48_ count	P15-0_ count	P15-6_ count	P15-48_ count	PC-0_TPM	PC-6_TPM	PC-48_ TPM	P15-0_TPM	P15-6_TPM	P15-48_ TPM
novel-m0269-3p	24	TTTTTTTTCTAGGACAGAGGGAGT	9679	1562	1640	1429	1610	1668	1770	1162.2785	1302.8964	1113.5398	1128.9863	1014.5584	1640.8397
novel-m0465-3p	21	TCAAACGTTGCAAGTAAAAAC	2851	531	434	516	499	488	383	395.1152	344.7909	402.0899	349.9156	296.8252	355.0517
novel-m0283-3p	22	TCAAACGTTGCAAGTAAAAACT	2674	503	414	476	473	455	353	374.2805	328.9019	370.9202	331.6836	276.753	327.2409
novel-m0215-3p	23	TCAAGGACCGTAGAATTGCTCTT	2671	417	452	427	448	475	452	310.2882	359.091	332.7372	314.1527	288.918	419.0167
novel-m0494-3p	22	TTAAACGTTGCAAGTAAAAACT	2435	450	361	430	429	441	324	334.8434	286.7961	335.0749	300.8293	268.2376	300.3571
novel-m0017-3p	23	TCAAGGACCGTAGAATTGCTCTT	2390	361	407	404	397	416	405	268.6188	323.3408	314.8146	278.3898	253.0314	375.4464
novel-m0569-3p	23	TCAAGGACCGTAGAATTGCTCTT	2231	323	375	392	361	395	385	240.3431	297.9184	305.4637	253.1454	240.2581	356.9058
novel-m0546-3p	20	TATTATAAGACGTTTTGACT	2157	377	361	421	343	314	341	280.5243	286.7961	328.0617	240.5232	190.99	316.1166
novel-m0454-5p	20	ATATTATAAGACGTTTTGAC	2114	369	356	411	339	305	334	274.5715	282.8239	320.2693	237.7182	185.5158	309.6274
novel-m0073-5p	21	TCATTGGCGATCGGATTATCC	1587	330	257	232	276	295	197	245.5518	204.1734	180.7846	193.5405	179.4333	182.6245
novel-m0228-5p	21	TCATTGGCGATCGGATTATCC	1587	330	257	232	276	295	197	245.5518	204.1734	180.7846	193.5405	179.4333	182.6245
novel-m0404-5p	21	TCATTGGCGATCGGATTATCC	1587	330	257	232	276	295	197	245.5518	204.1734	180.7846	193.5405	179.4333	182.6245
novel-m0131-5p	22	TAACGTTTGACTACTCGTCTTA	1580	256	280	420	192	221	211	190.4887	222.4457	327.2825	134.6369	134.4229	195.6029
novel-m0214-5p	22	TAACGTTTGACTACTCGTCTTA	1565	257	276	422	188	211	211	191.2328	219.2679	328.841	131.8319	128.3404	195.6029
novel-m0240-5p	22	TAACGTTTGACTACTCGTCTTA	1534	252	272	410	184	208	208	187.5123	216.0901	319.4901	129.027	126.5157	192.8218
novel-m0298-5p	22	TAACGTTTGACTACTCGTCTTA	1321	216	224	368	168	174	171	160.7248	177.9566	286.7618	117.8073	105.8352	158.5218
novel-m0487-5p	21	ACACAATAGATGTTCGGTCCT	1252	259	177	176	258	255	127	192.721	140.6175	137.147	180.9183	155.1034	117.7326
novel-m0269-5p	23	TCCCTCTATCCCAGAAAAAAACA	1119	206	158	129	259	189	178	153.2838	125.5229	100.5225	181.6195	114.959	165.011
novel-m0436-5p	21	ACACAATAGATGTTCGGTCCT	987	207	137	144	203	201	95	154.0279	108.8395	112.2111	142.3505	122.2579	88.0677
novel-m0356-5p	21	AACCAACCTAATACCGGATGT	977	129	164	212	159	142	171	95.9884	130.2896	165.1997	111.4962	86.3713	158.5218
novel-m0270-3p	23	AAACGTAGATTACTGATAAAACC	661	107	72	97	122	132	131	79.6183	57.2003	75.5867	85.5505	80.2888	121.4407
novel-m0009-3p	22	TTTGGCACCGTATTACTTTTCT	550	100	106	86	94	84	80	74.4096	84.2116	67.015	65.916	51.0929	74.1622
novel-m0508-3p	23	TTAAAACAGTTTGACTTTGACTA	546	112	93	96	84	87	74	83.3388	73.8838	74.8074	58.9036	52.9176	68.6001
novel-m0362-5p	21	TTTGGGCTAGGTATTTTCACC	492	77	72	80	102	97	64	57.2954	57.2003	62.3395	71.5258	59.0001	59.3298
novel-m0595-3p	21	CCTGCGGCATGTCGAGGGCCT	465	76	91	69	81	58	90	56.5513	72.2949	53.7678	56.7999	35.2784	83.4325
novel-m0375-3p	23	AGACGGACGGTCAAACGTTGAAC	403	72	70	68	68	70	55	53.5749	55.6114	52.9886	47.6839	42.5774	50.9865
novel-m0076-3p	22	AACAGTTTGACTTTGACCAAAG	384	75	60	75	57	69	48	55.8072	47.6669	58.4433	39.9703	41.9691	44.4973
novel-m0373-5p	23	TTTTCTAAACATGTTTGACCGTT	384	92	55	72	57	61	47	68.4569	43.6947	56.1056	39.9703	37.1032	43.5703
novel-m0554-5p	23	TTTTCTAAACATGTTTGACCGTT	381	92	55	72	56	59	47	68.4569	43.6947	56.1056	39.2691	35.8867	43.5703
novel-m0453-5p	23	CTTTTCTAAACATGTTTGACCGT	381	92	55	72	56	59	47	68.4569	43.6947	56.1056	39.2691	35.8867	43.5703
novel-m0156-5p	23	TTTTCTAAACATGTTTGACCGTT	381	92	55	72	56	59	47	68.4569	43.6947	56.1056	39.2691	35.8867	43.5703
novel-m0030-5p	20	CAATTCTACGGTCCTTGAGG	343	59	50	72	74	54	34	43.9017	39.7225	56.1056	51.8913	32.8454	31.519
novel-m0261-3p	23	AACGGTGTCAAGTATTTAGGAAC	342	43	55	37	67	93	47	31.9961	43.6947	28.832	46.9827	56.5671	43.5703
novel-m0338-5p	23	TTTTCTAAACATGTTTGACCGTT	335	80	51	60	51	53	40	59.5277	40.5169	46.7546	35.7629	32.2372	37.0811
novel-m0466-3p	23	AAGTAGTTTGACTTTGACCAAAG	332	62	54	61	48	45	62	46.134	42.9002	47.5339	33.6592	27.3712	57.4757
novel-m0553-3p	21	ACGAATCTCAGCCGTTGGATT	329	47	56	58	47	79	42	34.9725	44.4891	45.1962	32.958	48.0516	38.9352
novel-m0532-5p	21	ATTCGTCTACTTCATCACGAG	326	48	59	84	59	35	41	35.7166	46.8725	65.4565	41.3728	21.2887	38.0082
novel-m0531-3p	21	ACGAATCTCAGCCGTTGGATT	326	47	57	58	44	80	40	34.9725	45.2836	45.1962	30.8543	48.6599	37.0811
novel-m0405-5p	21	CCGGCGTCGGCTTCAGCCTGC	322	59	64	33	64	64	38	43.9017	50.8447	25.7151	44.879	38.9279	35.2271
novel-m0274-3p	23	GAAGTGGAGTTGTGGAGCTGCCT	287	68	42	48	45	48	36	50.5986	33.3669	37.4037	31.5555	29.1959	33.373
novel-m0439-3p	23	TCAAGGACCGTAAAATTGCTCTT	273	44	58	45	37	37	52	32.7402	46.078	35.066	25.9456	22.5052	48.2055
novel-m0041-3p	23	GAAGTGGAGTTGTGGAGCTGCCT	267	65	35	47	43	42	35	48.3663	27.8057	36.6245	30.1531	25.5464	32.446
novel-m0212-3p	21	TATATGAATGTAGATAATGCT	249	32	27	50	32	53	55	23.8111	21.4501	38.9622	22.4395	32.2372	50.9865
novel-m0342-3p	23	TTTCCATGTCCAACTTTGACTGT	244	48	44	34	45	42	31	35.7166	34.9558	26.4943	31.5555	25.5464	28.7379
novel-m0542-3p	23	TCAAGGACCGTAAAATTGCTCTT	241	44	46	35	34	34	48	32.7402	36.5447	27.2735	23.8419	20.6804	44.4973
novel-m0315-5p	21	TAGTTTTACTTTGGATCACCC	239	36	62	31	39	42	29	26.7875	49.2558	24.1566	27.3481	25.5464	26.8838
novel-m0490-5p	20	AACGAAGTTGAGTTTGAACT	237	22	34	53	37	38	53	16.3701	27.0113	41.2999	25.9456	23.1134	49.1325
novel-m0055-3p	21	AGCGCCACGTCGTCGGTCAGC	211	35	34	27	44	38	33	26.0434	27.0113	21.0396	30.8543	23.1134	30.5919
novel-m0211-3p	23	ATAGATTTGTAGCCAGCTATAGC	210	40	39	38	31	30	32	29.7639	30.9835	29.6113	21.7382	18.2475	29.6649
novel-m0520-3p	22	TTAATGTATGACGACGTTGACT	200	33	37	35	31	25	39	24.5552	29.3946	27.2735	21.7382	15.2062	36.1541
novel-m0264-3p	22	TTAATTCATTGATTGTTTTCAG	198	32	30	39	26	29	42	23.8111	23.8335	30.3905	18.2321	17.6392	38.9352
novel-m0254-3p	22	AAAATGCTAATCTGAGGCCAGC	195	45	25	32	41	19	33	33.4843	19.8612	24.9358	28.7506	11.5567	30.5919
novel-m0327-3p	22	TTGGCTGCATCCCGTTCTCCTC	191	40	48	23	21	24	35	29.7639	38.1336	17.9226	14.7259	14.598	32.446
novel-m0321-5p	23	TGAGGGCTAGAGCCTGCCTCTGG	191	40	45	5	66	27	8	29.7639	35.7502	3.8962	46.2814	16.4227	7.4162
novel-m0101-5p	23	TTTGACACCGTTGACTTTTTAGA	191	41	32	13	29	49	27	30.5079	25.4224	10.1302	20.3358	29.8042	25.0298
novel-m0059-5p	24	GTTTCCGTGTCTAACATTTGACTG	191	40	41	24	36	28	22	29.7639	32.5724	18.7019	25.2444	17.031	20.3946
novel-m0509-3p	21	ACTATGGCTGCACTTAAAATG	190	33	35	28	23	40	31	24.5552	27.8057	21.8188	16.1284	24.3299	28.7379
novel-m0367-3p	23	TCAAACGTTGGACACGGAAACCT	188	44	30	24	35	26	29	32.7402	23.8335	18.7019	24.5432	15.8145	26.8838
novel-m0283-5p	22	TTTTTACTTGTAACGTTTGACC	180	31	37	35	25	29	23	23.067	29.3946	27.2735	17.5308	17.6392	21.3216
novel-m0182-5p	22	TTTTTACTTACAACGTTTGACC	177	35	29	40	24	22	27	26.0434	23.039	31.1698	16.8296	13.3815	25.0298
novel-m0448-3p	23	TTGGATTGAAGCCAAAACAGCCT	168	39	27	27	27	26	22	29.0198	21.4501	21.0396	18.9333	15.8145	20.3946
novel-m0406-3p	21	TTAAGGACCGTAAAATTGCTC	167	21	31	34	31	19	31	15.626	24.6279	26.4943	21.7382	11.5567	28.7379
novel-m0157-5p	22	TTCTAGTACAATGAATTTGGAC	165	30	32	21	30	25	27	22.3229	25.4224	16.3641	21.037	15.2062	25.0298
novel-m0171-5p	21	TTTGAGGAGGTACCATGAGGT	165	39	30	17	35	29	15	29.0198	23.8335	13.2471	24.5432	17.6392	13.9054
novel-m0249-3p	21	TTCAGATTCGTAGTATTATGA	160	26	26	42	16	18	32	19.3465	20.6557	32.7283	11.2197	10.9485	29.6649
novel-m0084-5p	22	TTTCCGTATCCAACGTTTGACT	160	20	28	37	21	20	34	14.8819	22.2446	28.832	14.7259	12.165	31.519
novel-m0550-5p	22	TTTCCGTATCCAACGTTTGACT	160	20	28	37	21	20	34	14.8819	22.2446	28.832	14.7259	12.165	31.519

novel-m0450-3p	23	TAGTTATTTGCCACATCTAAAAG	150	24	33	27	12	14	40	17.8583	26.2168	21.0396	8.4148	8.5155	37.0811
novel-m0447-5p	21	GTCACGGCGGCGGCGAGCCTC	148	27	20	12	36	34	19	20.0906	15.889	9.3509	25.2444	20.6804	17.6135
novel-m0371-3p	22	TCAAACGTTGGACACGGATATC	147	31	34	27	18	19	18	23.067	27.0113	21.0396	12.6222	11.5567	16.6865
novel-m0322-3p	24	CAGATTCGTAGTCCTAGGATGTGT	146	20	29	42	18	19	18	14.8819	23.039	32.7283	12.6222	11.5567	16.6865
novel-m0420-3p	21	TCGTCATCGCCTTGTCGGAGG	146	19	30	17	21	33	26	14.1378	23.8335	13.2471	14.7259	20.0722	24.1027
novel-m0360-3p	23	AAACCGGGACTAAAGATCGAGCT	145	27	28	19	33	20	18	20.0906	22.2446	14.8056	23.1407	12.165	16.6865
novel-m0392-3p	21	GTCAAACGTTGGACACGGATA	144	29	34	26	18	19	18	21.5788	27.0113	20.2603	12.6222	11.5567	16.6865
novel-m0322-5p	23	ACATCCTAGGACATGAATCTGGA	143	22	16	29	21	29	26	16.3701	12.7112	22.5981	14.7259	17.6392	24.1027
novel-m0052-5p	22	TTAATATATGACGTCGTTGACT	143	34	23	34	22	9	21	25.2993	18.2723	26.4943	15.4271	5.4742	19.4676
novel-m0328-5p	22	TAGGTCAAATTAGTAAGTAACC	140	24	22	18	22	21	33	17.8583	17.4779	14.0264	15.4271	12.7732	30.5919
novel-m0302-3p	21	TTCAGATTCGTAGTATTATGA	136	24	22	36	16	12	26	17.8583	17.4779	28.0528	11.2197	7.299	24.1027
novel-m0496-5p	21	TTTGAGGAGGTACCATGAGGT	135	33	25	16	27	24	10	24.5552	19.8612	12.4679	18.9333	14.598	9.2703
novel-m0440-5p	20	TTTTTATTCAACGTTTGACT	131	19	13	22	33	24	20	14.1378	10.3278	17.1434	23.1407	14.598	18.5406
novel-m0174-5p	22	TTAATATATGACGTCGTTGACT	130	31	21	32	20	8	18	23.067	16.6834	24.9358	14.0247	4.866	16.6865
novel-m0533-5p	23	TAACCGGGACTAAAAATGATCTT	128	22	24	22	22	27	11	16.3701	19.0668	17.1434	15.4271	16.4227	10.1973
novel-m0266-5p	22	TTAATAGATGACGTTGTTGACT	127	28	35	26	18	13	7	20.8347	27.8057	20.2603	12.6222	7.9072	6.4892
novel-m0314-5p	22	TTTTTATTTGAACTGTTTGACC	124	20	22	26	24	17	15	14.8819	17.4779	20.2603	16.8296	10.3402	13.9054
novel-m0567-3p	21	AGACCAAGGAGAAATTAACTC	123	24	21	21	25	13	19	17.8583	16.6834	16.3641	17.5308	7.9072	17.6135
novel-m0458-5p	21	GTGCGCCAATGCTTTTCAAGG	122	25	29	9	36	17	6	18.6024	23.039	7.0132	25.2444	10.3402	5.5622
novel-m0210-5p	22	TTCTAGTACAATGAATTTGGAC	117	25	25	13	17	20	17	18.6024	19.8612	10.1302	11.921	12.165	15.7595
novel-m0051-3p	22	TTAAAACAGTTTGACTTTAACC	113	14	15	23	18	24	19	10.4173	11.9167	17.9226	12.6222	14.598	17.6135
novel-m0517-3p	22	TTAAAACAGTTTGACTTTAACC	113	14	15	24	17	24	19	10.4173	11.9167	18.7019	11.921	14.598	17.6135
novel-m0309-3p	21	AATCGGAAAAGATTTGGTACC	109	0	0	0	39	34	36	0.01	0.01	0.01	27.3481	20.6804	33.373
novel-m0144-3p	22	TTGCGAGATGTAGAAGTTGATT	107	14	26	26	14	16	11	10.4173	20.6557	20.2603	9.8173	9.732	10.1973
novel-m0164-3p	21	CTCGCCCCGTCGGTCTCCTCC	106	21	19	6	20	31	9	15.626	15.0945	4.6755	14.0247	18.8557	8.3433
novel-m0034-3p	21	TCATGCGCACATTATTAACTG	105	14	15	21	24	19	12	10.4173	11.9167	16.3641	16.8296	11.5567	11.1243
novel-m0083-3p	22	TTAGAGTAGTTTGACTTTGACT	103	24	12	19	18	16	14	17.8583	9.5334	14.8056	12.6222	9.732	12.9784
novel-m0364-5p	22	TTAATAGATGACGTTGTTGACT	102	21	26	23	16	10	6	15.626	20.6557	17.9226	11.2197	6.0825	5.5622
novel-m0252-5p	21	CGTTTGACCGTGGTCTTGTCT	101	16	22	17	17	16	13	11.9055	17.4779	13.2471	11.921	9.732	12.0514
novel-m0024-5p	21	CGTTTGACCGTGGTCTTGTCT	101	16	22	17	17	16	13	11.9055	17.4779	13.2471	11.921	9.732	12.0514
novel-m0062-3p	22	CAAAATTTTCCATGCACTTCGA	100	15	28	7	12	24	14	11.1614	22.2446	5.4547	8.4148	14.598	12.9784

Supplemental Table S3. The target prediction of the differently expressed miRNAs in P15-0/PC-0. The blue region represents the 67 potential targets whose expression pattern was the opposite of one of the seven miRNAs.

miR-name	PC-0-count	P15-0-count	PC-0-TPM	P15-0-TPM	Fold-change (log_2 P15-0/PC-0)	P-value	Sig-label	Gene ID	Description	PC_0_FPKM	P15_0_FPKM	Log_2 (fold-changed)	P-value	FDR
osa-miR531b	0	16	0.01	11.2197	6.98683254	3.07E-05	**	LOC_Os02g45700	fringe-related protein, putative, expressed	1.4592	0.000335491	-12.08661595	0.494986094	1
osa-miR530-5p	23	67	17.1142	46.9827	1.487976882	1.14E-05	**	LOC_Os04g51400	zinc finger, C3HC4 type domain containing protein, expressed	1.45055	0.000471833	-11.58603608	0.494986094	1
osa-miR531b	0	16	0.01	11.2197	6.98683254	3.07E-05	**	LOC_Os03g12360	trehalose-6-phosphate synthase, putative, expressed	0.667649	0.225714	-1.564593936	0.494986094	1
osa-miR531b	0	16	0.01	11.2197	6.98683254	3.07E-05	**	LOC_Os05g37640	expressed protein	0.943922	0.327381	-1.527697053	0.49498641	1
osa-miR3979-3p	3	15	2.2323	10.5185	2.226604058	0.007563544	**	LOC_Os02g58720	peroxidase precursor, putative, expressed	11.7011	4.59361	-1.34894388	0.093202666	1
osa-miR531b	0	16	0.01	11.2197	6.98683254	3.07E-05	**	LOC_Os07g36700	CSLF1 - cellulose synthase-like family F; beta1,3;1,4 glucan synthase, expressed	1.67282	0.689292	-1.279095039	0.621277411	1
osa-miR3979-3p	3	15	2.2323	10.5185	2.226604058	0.007563544	**	LOC_Os02g10150	bZIP transcription factor, putative, expressed	3.00232	1.38754	-1.113548392	0.370061833	1
osa-miR531b	0	16	0.01	11.2197	6.98683254	3.07E-05	**	LOC_Os12g35610	respiratory burst oxidase, putative, expressed	0.636272	0.295081	-1.108532605	0.49498641	1
osa-miR530-5p	23	67	17.1142	46.9827	1.487976882	1.14E-05	**	LOC_Os07g16600	expressed protein	0.155713	0.075033	-1.053292249	1	1
osa-miR530-5p	23	67	17.1142	46.9827	1.487976882	1.14E-05	**	LOC_Os02g14990	zinc finger, C3HC4 type domain containing protein, expressed	3.24107	1.59124	-1.026318732	0.684429373	1
osa-miR1846d-3p	6	17	4.4646	11.921	1.434409235	0.034791993	*	LOC_Os07g38830	hydrolase, alpha/beta fold family domain containing protein, expressed	1.467799082	1.2173141	-0.987496715	2.74118	1.7073269
osa-miR531b	0	16	0.01	11.2197	6.98683254	3.07E-05	**	LOC_Os01g40640	S1 RNA binding domain containing protein, expressed	10.3194	5.35442	-0.946556877	0.205840628	1
osa-miR1846d-3p	6	17	4.4646	11.921	1.434409235	0.034791993	*	LOC_Os02g22260	fruit protein PKIWI502, putative, expressed	0.615274	0.810465	-0.788427493	1.13892	1.63937
osa-miR531b	0	16	0.01	11.2197	6.98683254	3.07E-05	**	LOC_Os08g41670	uncharacterized protein family UPF0016 domain containing protein, expressed	2.04421	1.22554	-0.738125838	0.621277411	1
osa-miR531b	0	16	0.01	11.2197	6.98683254	3.07E-05	**	LOC_Os11g20160	O-methyltransferase, putative, expressed	2.37086	1.43494	-0.72442006	0.621277643	1
osa-miR531b	0	16	0.01	11.2197	6.98683254	3.07E-05	**	LOC_Os01g02310	expressed protein	0.103829	0.0653722	-0.667460298	1	1
osa-miR531b	0	16	0.01	11.2197	6.98683254	3.07E-05	**	LOC_Os08g33590	basic helix-loop-helix, putative, expressed	10.6086	7.03088	-0.593457102	0.354829178	1
osa-miR3979-3p	3	15	2.2323	10.5185	2.226604058	0.007563544	**	LOC_Os04g45000	short-chain dehydrogenase/reductase, putative, expressed	3.25812	2.38206	-0.451829988	0.684429562	1
osa-miR530-5p	23	67	17.1142	46.9827	1.487976882	1.14E-05	**	LOC_Os04g51400	zinc finger, C3HC4 type domain containing protein, expressed	1.39137	1.0279	-0.436806202	1	1
osa-miR528-3p	21	48	15.626	33.6592	1.138446186	0.002223801	**	LOC_Os02g03040	RNA recognition motif containing protein, expressed	15.2213	11.2758	-0.432861786	0.438029849	1
osa-miR531b	0	16	0.01	11.2197	6.98683254	3.07E-05	**	LOC_Os06g08470	expressed protein	28.158436	20.930053	-0.42799124	0.31853837	1
osa-miR1846d-3p	6	17	4.4646	11.921	1.434409235	0.034791993	*	LOC_Os07g38830	hydrolase, alpha/beta fold family domain containing protein, expressed	1.30315	2.20035	-0.408944261	1.96183	0.93657
osa-miR3979-3p	3	15	2.2323	10.5185	2.226604058	0.007563544	**	LOC_Os07g10620	expressed protein	6.24936	4.71192	-0.407393405	0.772265723	1
osa-miR3979-3p	3	15	2.2323	10.5185	2.226604058	0.007563544	**	LOC_Os02g58720	peroxidase precursor, putative, expressed	1.5785	1.19371	-0.40310187	0.621277411	1
osa-miR1846d-3p	6	17	4.4646	11.921	1.434409235	0.034791993	*	LOC_Os06g10920	xyloglucan fucosyltransferase, putative, expressed	1.33688	0.416729	-0.373312416	0.702099	0.790368
osa-miR1846d-3p	6	17	4.4646	11.921	1.434409235	0.034791993	*	LOC_Os04g54560	transferase family protein, putative, expressed	6.44963	6.01908	-0.366075876	4.69582	3.85729
osa-miR1846d-3p	6	17	4.4646	11.921	1.434409235	0.034791993	*	LOC_Os01g13720	expressed protein	1.74886	1.06565	-0.344580218	3.77087	1.12813
osa-miR1320-3p	1	9	0.7441	6.3111	2.973396978	0.021515144	*	LOC_Os08g19610	peptidyl-prolyl cis-trans isomerase, putative, expressed	0.722903	0.2592098	-0.344089223	1.02732	0.993739
osa-miR3979-3p	3	15	2.2323	10.5185	2.226604058	0.007563544	**	LOC_Os01g49760	expressed protein	0.660763	0.523176	-0.336836543	1	1
osa-miR531b	0	16	0.01	11.2197	6.98683254	3.07E-05	**	LOC_Os12g35610	respiratory burst oxidase, putative, expressed	1.58136	1.274	-0.31180056	0.621277411	1
osa-miR1320-3p	1	9	0.7441	6.3111	2.973396978	0.021515144	*	LOC_Os08g19610	peptidyl-prolyl cis-trans isomerase, putative, expressed	0.255762	0.368982	-0.256987985	0.484112	0.221476
osa-miR531b	0	16	0.01	11.2197	6.98683254	3.07E-05	**	LOC_Os10g01134	OsSCP46 - Putative Serine Carboxypeptidase homologue, expressed	8.92034	7.57744	-0.235388176	0.812819227	1
osa-miR528-3p	21	48	15.626	33.6592	1.138446186	0.002223801	**	LOC_Os02g07200	protein-O-fucosyltransferase 1, putative, expressed	8.30656	7.096968	-0.227048335	0.80178879	1
osa-miR1846d-3p	6	17	4.4646	11.921	1.434409235	0.034791993	*	LOC_Os02g49760	potassium transporter, putative, expressed	8.3749	2.26657	-0.213894482	6.7443	2.94555
osa-miR531b	0	16	0.01	11.2197	6.98683254	3.07E-05	**	LOC_Os01g59819	Os1bglu2 - beta-glucosidase homologue, similar to G. max isohydroxyurate hydrolase, expressed	43.096505	37.33049	-0.20721643	0.502650398	1
osa-miR528-3p	21	48	15.626	33.6592	1.138446186	0.002223801	**	LOC_Os02g03040	RNA recognition motif containing protein, expressed	1.8314	1.5959	-0.198576673	1	1
osa-miR531b	0	16	0.01	11.2197	6.98683254	3.07E-05	**	LOC_Os10g07229	dehydrogenase, putative, expressed	29.0503	25.3596	-0.196021072	0.587302972	1

osa-miR1846d-3p	6	17	4.4646	11.921	1.434409235	0.034791993	*	LOC_Os01g13720	expressed protein	1.10317	1.01739	-0.192884868	2.4068	1.65595
osa-miR3979-3p	3	15	2.2323	10.5185	2.226604058	0.007563544	**	LOC_Os10g38314	glutathione S-transferase, N-terminal domain containing protein, expressed	1.31956	1.15667	-0.190079631	1	1
osa-miR1846d-3p	6	17	4.4646	11.921	1.434409235	0.034791993	*	LOC_Os01g05810	gamma-glutamyltranspeptidase 1 precursor, putative, expressed	21.81269	16.31241	-0.188642639	21.30055	14.57073
osa-miR531b	0	16	0.01	11.2197	6.98683254	3.07E-05	**	LOC_Os09g03750	ankyrin, putative, expressed	3.627117	3.196418271	-0.182367077	0.723903288	1
osa-miR531b	0	16	0.01	11.2197	6.98683254	3.07E-05	**	LOC_Os04g21340	expressed protein	1.116161	0.9940272	-0.167187908	1	1
osa-miR1846d-3p	6	17	4.4646	11.921	1.434409235	0.034791993	*	LOC_Os01g61940	white-brown complex homolog protein, putative, expressed	9.07698	9.14711	-0.163849833	14.4821	7.97644
osa-miR531b	0	16	0.01	11.2197	6.98683254	3.07E-05	**	LOC_Os04g33290	cysteine protease, putative, expressed	2.03448	1.83637	-0.147803329	1	1
osa-miR1846d-3p	6	17	4.4646	11.921	1.434409235	0.034791993	*	LOC_Os02g49760	potassium transporter, putative, expressed	11.6092	14.9919	-0.136302784	13.9797	13.4903
osa-miR530-5p	23	67	17.1142	46.9827	1.487976882	1.14E-05	**	LOC_Os04g43030	lipase class 3 family protein, putative, expressed	4.784156	4.353923	-0.13594854	0.751537927	1
osa-miR531b	0	16	0.01	11.2197	6.98683254	3.07E-05	**	LOC_Os02g51570	peptidyl-prolyl cis-trans isomerase, FKBP-type, putative, expressed	58.68281	54.06921	-0.118130678	0.573866077	1
osa-miR1846d-3p	6	17	4.4646	11.921	1.434409235	0.034791993	*	LOC_Os01g49410	expressed protein	20.4767	19.5374	-0.101113971	14.2479	15.3003
osa-miR531b	0	16	0.01	11.2197	6.98683254	3.07E-05	**	LOC_Os01g04300	glycosyl hydrolase family 10 protein, putative, expressed	32.2872	30.1456	-0.099014889	0.799946519	1
osa-miR530-5p	23	67	17.1142	46.9827	1.487976882	1.14E-05	**	LOC_Os03g52840	serine hydroxymethyltransferase, mitochondrial precursor, putative, expressed	208.181	194.566	-0.09757878	0.397562517	1
osa-miR3979-3p	3	15	2.2323	10.5185	2.226604058	0.007563544	**	LOC_Os01g56330	TKL_IRAK_CrRLK1L-1.5 - The CrRLK1L-1 subfamily has homology to the CrRLK1L homolog, expressed	44.8441	41.9676	-0.095642224	0.747943467	1
osa-miR530-5p	23	67	17.1142	46.9827	1.487976882	1.14E-05	**	LOC_Os07g05010	helix-loop-helix DNA-binding domain containing protein, expressed	9.36105	8.77234	-0.093708632	1	1
osa-miR528-3p	21	48	15.626	33.6592	1.138446186	0.002223801	**	LOC_Os09g13820	pollen signalling protein with adenylyl cyclase activity, putative, expressed	0.97952	0.919529	-0.091179875	1	1
osa-miR528-3p	21	48	15.626	33.6592	1.138446186	0.002223801	**	LOC_Os02g03040	RNA recognition motif containing protein, expressed	29.8992	28.2883	-0.079901404	0.793400446	1
osa-miR1320-3p	1	9	0.7441	6.3111	2.973396978	0.021515144	*	LOC_Os08g19610	peptidyl-prolyl cis-trans isomerase, putative, expressed	0.583676	0.967072	-0.075026529	0.348197	0.40864
osa-miR529b	32	71	23.8111	49.7876	1.097200205	0.000352974	**	LOC_Os05g49520	CTP synthase, putative, expressed	0.0295927	0.0281345	-0.072901008	1	1
osa-miR531b	0	16	0.01	11.2197	6.98683254	3.07E-05	**	LOC_Os12g13300	expressed protein	12.208	11.6461	-0.067979956	1	1
osa-miR1846d-3p	6	17	4.4646	11.921	1.434409235	0.034791993	*	LOC_Os06g05980	transporter family protein, putative, expressed	8.8795	10.9304	-0.06753557	12.2296	8.75929
osa-miR3979-3p	3	15	2.2323	10.5185	2.226604058	0.007563544	**	LOC_Os10g42610	expressed protein	10.8628	10.3894	-0.064283681	0.830295071	1
osa-miR531b	0	16	0.01	11.2197	6.98683254	3.07E-05	**	LOC_Os09g34250	UDP-glucoronosyl and UDP-glucosyl transferase domain containing protein, expressed	2.23158	2.14668	-0.055958378	1	1
osa-miR531b	0	16	0.01	11.2197	6.98683254	3.07E-05	**	LOC_Os01g52214	ubiquinone oxidoreductase, putative, expressed	121.106	116.818	-0.052007753	0.697603169	1
osa-miR3979-3p	3	15	2.2323	10.5185	2.226604058	0.007563544	**	LOC_Os10g42610	expressed protein	1.50233	1.46114	-0.040107331	0.62127788	1
osa-miR1846d-3p	6	17	4.4646	11.921	1.434409235	0.034791993	*	LOC_Os01g54440	expressed protein	2.43813	3.11467	-0.039746641	2.44202	1.97411
osa-miR531b	0	16	0.01	11.2197	6.98683254	3.07E-05	**	LOC_Os01g13740	myb-like DNA-binding domain containing protein, expressed	39.9172	39.77664	-0.005089112	1	1
osa-miR1320-3p	1	9	0.7441	6.3111	2.973396978	0.021515144	*	LOC_Os08g19610	peptidyl-prolyl cis-trans isomerase, putative, expressed	14.488	15.0906	-0.003915863	13.023	17.8689
osa-miR1846d-3p	6	17	4.4646	11.921	1.434409235	0.034791993	*	LOC_Os03g07340	ribosomal L18p/L5e family protein, putative, expressed	7.32623	7.546078	-0.002039052	8.54666	9.283562
osa-miR3979-3p	3	15	2.2323	10.5185	2.226604058	0.007563544	**	LOC_Os11g43970	AAA-type ATPase family protein, putative, expressed	32.8971	32.8614	-0.001566466	1	1
osa-miR531b	0	16	0.01	11.2197	6.98683254	3.07E-05	**	LOC_Os07g04520	protein kinase, putative, expressed	0.000394635	0	-Inf	1	1
osa-miR531b	0	16	0.01	11.2197	6.98683254	3.07E-05	**	LOC_Os09g33690	Os9bglu32 - beta-glucosidase homologue, similar to G. max hydroxyisourate hydrolase, expressed	0.967041	0	-Inf	0.494986094	1
osa-miR3979-3p	3	15	2.2323	10.5185	2.226604058	0.007563544	**	LOC_Os03g42780	zinc finger protein-related, putative, expressed	0.0158732	0	-Inf	1	1
osa-miR3979-3p	3	15	2.2323	10.5185	2.226604058	0.007563544	**	LOC_Os05g29800	expressed protein	0.000619013	0	-Inf	1	1
osa-miR3979-3p	3	15	2.2323	10.5185	2.226604058	0.007563544	**	LOC_Os06g49380	NBS-LRR disease resistance protein, putative, expressed	0.0926166	0	-Inf	1	1
osa-miR3979-3p	3	15	2.2323	10.5185	2.226604058	0.007563544	**	LOC_Os06g49380	NBS-LRR disease resistance protein, putative, expressed	2.46E-05	0	-Inf	1	1
osa-miR530-5p	23	67	17.1142	46.9827	1.487976882	1.14E-05	**	LOC_Os03g52840	serine hydroxymethyltransferase, mitochondrial precursor, putative, expressed	0.180402	0	-Inf	1	1
osa-miR1846d-3p	6	17	4.4646	11.921	1.434409235	0.034791993	*	LOC_Os01g13720	expressed protein	0.536291	0.389927	-Inf	1.49231	0.75597
osa-miR1846d-3p	6	17	4.4646	11.921	1.434409235	0.034791993	*	LOC_Os03g62670	expressed protein	3.24E-05	0	-Inf	0.2084	0
osa-miR1846d-3p	6	17	4.4646	11.921	1.434409235	0.034791993	*	LOC_Os09g26770	ribosomal L18p/L5e family protein, putative, expressed	0	0	-Inf	0.271759	0.396614

osa-miR1846d-3p	6	17	4.4646	11.921	1.434409235	0.034791993	*	LOC_Os06g10260	cytochrome P450, putative, expressed	0	0.340431	-Inf	0.286402	0
osa-miR167a-3p	172	80	127.9846	56.0987	-1.152675764	1.26E-09	**	LOC_Os03g58260	indole-3-glycerol phosphate lyase, chloroplast precursor, putative, expressed	0.400844	0.2155557	-0.894980167	1	1
osa-miR167a-3p	172	80	127.9846	56.0987	-1.152675764	1.26E-09	**	LOC_Os03g58260	indole-3-glycerol phosphate lyase, chloroplast precursor, putative, expressed	5.94458	5.10455	-0.2197911	0.772265723	1
osa-miR3979-3p	3	15	2.2323	10.5185	2.226604058	0.007563544	**	LOC_Os04g54900	bHLH family protein, putative, expressed	6.00621	6.02257	0.003924339	1	1
osa-miR529b	32	71	23.8111	49.7876	1.097200205	0.000352974	**	LOC_Os01g03950	glycosyl hydrolase, family 31, putative, expressed	15.0337	15.0778	0.00422582	1	1
osa-miR1846d-3p	6	17	4.4646	11.921	1.434409235	0.034791993	*	LOC_Os05g38000	ATROPGEF7/ROPGEF7, putative, expressed	58.5492	76.2295	0.024077534	86.688	94.1303
osa-miR530-5p	23	67	17.1142	46.9827	1.487976882	1.14E-05	**	LOC_Os06g36990	phospholipid-transporting ATPase 4, putative, expressed	14.28371	14.55304	0.026949802	1	1
osa-miR3979-3p	3	15	2.2323	10.5185	2.226604058	0.007563544	**	LOC_Os02g58720	peroxidase precursor, putative, expressed	76.5106	78.0807	0.02930635	1	1
osa-miR528-3p	21	48	15.626	33.6592	1.138446186	0.002223801	**	LOC_Os02g08140	CAMK_KIN1/SNF1/Nim1_like. 14 - CAMK includes calcium/calmodulin depedent protein kinases, expressed	5.068278	5.178954	0.031165084	1	1
osa-miR530-5p	23	67	17.1142	46.9827	1.487976882	1.14E-05	**	LOC_Os07g05010	helix-loop-helix DNA-binding domain containing protein, expressed	4.42535	4.52595	0.032429085	1	1
osa-miR1846d-3p	6	17	4.4646	11.921	1.434409235	0.034791993	*	LOC_Os02g22260	fruit protein PKIWI502, putative, expressed	52.2703	89.6217	0.041383099	126.392	119.312
osa-miR531b	0	16	0.01	11.2197	6.98683254	3.07E-05	**	LOC_Os09g36990	expressed protein	65.699	67.6636	0.042508525	1	1
osa-miR3979-3p	3	15	2.2323	10.5185	2.226604058	0.007563544	**	LOC_Os05g13780	C-5 cytosine-specific DNA methylase, putative, expressed	14.90328	15.425762	0.049711875	1	1
osa-miR3979-3p	3	15	2.2323	10.5185	2.226604058	0.007563544	**	LOC_Os01g49760	expressed protein	6.06851	6.30118	0.054279687	1	1
osa-miR3979-3p	3	15	2.2323	10.5185	2.226604058	0.007563544	**	LOC_Os03g44780	elongation factor Tu family protein, putative, expressed	2.0618	2.15318	0.062564536	1	1
osa-miR1846d-3p	6	17	4.4646	11.921	1.434409235	0.034791993	*	LOC_Os11g40510	RNA recognition motif containing protein, putative, expressed	103.879	85.5242	0.063289445	66.7516	101.594
osa-miR3979-3p	3	15	2.2323	10.5185	2.226604058	0.007563544	**	LOC_Os01g63880	expressed protein	15.37502001	16.14152	0.070188152	1	1
osa-miR530-5p	23	67	17.1142	46.9827	1.487976882	1.14E-05	**	LOC_Os07g08430	indole-3-glycerol phosphate lyase, chloroplast precursor, putative, expressed	19.1507	20.2431	0.080033112	1	1
osa-miR531b	0	16	0.01	11.2197	6.98683254	3.07E-05	**	LOC_Os10g34420	lysM domain containing protein, putative, expressed	4.33108	4.583242	0.081641643	1	1
osa-miR3979-3p	3	15	2.2323	10.5185	2.226604058	0.007563544	**	LOC_Os04g45000	short-chain dehydrogenase/reductase, putative, expressed	4.84861	5.23018	0.109289384	1	1
osa-miR531b	0	16	0.01	11.2197	6.98683254	3.07E-05	**	LOC_Os03g12360	trehalose-6-phosphate synthase, putative, expressed	24.4038	26.3439	0.110363128	0.888004923	1
osa-miR3979-3p	3	15	2.2323	10.5185	2.226604058	0.007563544	**	LOC_Os01g32880	AP-3 complex subunit delta, putative, expressed	5.84578	6.31549	0.111499137	1	1
osa-miR1846d-3p	6	17	4.4646	11.921	1.434409235	0.034791993	*	LOC_Os06g50960	expansin precursor, putative, expressed	7.989359333	14.62024	0.111728527	12.447297	9.692248
osa-miR530-5p	23	67	17.1142	46.9827	1.487976882	1.14E-05	**	LOC_Os04g51400	zinc finger, C3HC4 type domain containing protein, expressed	10.49158	11.39744	0.119477855	1	1
osa-miR531b	0	16	0.01	11.2197	6.98683254	3.07E-05	**	LOC_Os05g49940	expressed protein	2.49441	2.71316	0.121275512	1	1
osa-miR531b	0	16	0.01	11.2197	6.98683254	3.07E-05	**	LOC_Os01g08220	gibberellin 3-beta-dioxygenase 2-2, putative, expressed	9.13775	9.96106	0.124460302	1	1
osa-miR531b	0	16	0.01	11.2197	6.98683254	3.07E-05	**	LOC_Os03g58010	acetyltransferase, GNAT family, putative, expressed	1.07437	1.17288	0.12656449	1	1
osa-miR530-5p	23	67	17.1142	46.9827	1.487976882	1.14E-05	**	LOC_Os04g56980	SWIB/MDM2 domain containing protein, expressed	16.5527	18.2219	0.138606835	1	1
osa-miR1846d-3p	6	17	4.4646	11.921	1.434409235	0.034791993	*	LOC_Os02g49800	eukaryotic aspartyl protease domain containing protein, expressed	48.0694	42.81	0.140898302	33.1129	38.1874
osa-miR528-3p	21	48	15.626	33.6592	1.138446186	0.002223801	**	LOC_Os02g07200	protein-O-fucosyltransferase 1, putative, expressed	5.6092	6.19018	0.142186337	1	1
osa-miR3979-3p	3	15	2.2323	10.5185	2.226604058	0.007563544	**	LOC_Os05g43582	tRNA methyltran, putative, expressed	5.531643	6.13736549	0.14991145	1	1
osa-miR1846d-3p	6	17	4.4646	11.921	1.434409235	0.034791993	*	LOC_Os09g26770	ribosomal L18p/L5e family protein, putative, expressed	16.8408408	16.57788098	0.152967037	13.46533027	14.0338215
osa-miR531b	0	16	0.01	11.2197	6.98683254	3.07E-05	**	LOC_Os03g52760	kinase, pfkB family, putative, expressed	6.39352	7.117485	0.154757111	1	1
osa-miR530-5p	23	67	17.1142	46.9827	1.487976882	1.14E-05	**	LOC_Os04g51400	zinc finger, C3HC4 type domain containing protein, expressed	4.49655	5.20961	0.212356863	1	1
osa-miR531b	0	16	0.01	11.2197	6.98683254	3.07E-05	**	LOC_Os09g28470	leucine-rich repeat family protein, putative, expressed	1.406634	1.652159	0.232105541	1	1
osa-miR531b	0	16	0.01	11.2197	6.98683254	3.07E-05	**	LOC_Os06g05940	DTA2, putative, expressed	1.5331	1.81575	0.244113777	1	1
osa-miR530-5p	23	67	17.1142	46.9827	1.487976882	1.14E-05	**	LOC_Os07g13950	expressed protein	11.9548	14.26548	0.254938297	0.845220176	1
osa-miR3979-3p	3	15	2.2323	10.5185	2.226604058	0.007563544	**	LOC_Os02g54200	mitochondrial transcription termination factor family protein, putative, expressed	5.970785	7.17407017	0.264871236	1	1
osa-miR530-5p	23	67	17.1142	46.9827	1.487976882	1.14E-05	**	LOC_Os11g04520	PP2A regulatory subunit TAP46, putative, expressed	1.31051	1.57744	0.267456769	1	1

osa-miR529b	32	71	23.8111	49.7876	1.097200205	0.000352974	**	LOC_Os01g69830	OsSPL2 - SBP-box gene family member, expressed	20.5927	24.8999	0.274006948	0.659476779	1
osa-miR530-5p	23	67	17.1142	46.9827	1.487976882	1.14E-05	**	LOC_Os11g04520	PP2A regulatory subunit TAP46, putative, expressed	2.39639	2.93049	0.290279196	1	1
osa-miR1846d-3p	6	17	4.4646	11.921	1.434409235	0.034791993	*	LOC_Os02g49760	potassium transporter, putative, expressed	3.97286	7.30414	0.291815689	5.34169	6.06538
osa-miR1846d-3p	6	17	4.4646	11.921	1.434409235	0.034791993	*	LOC_Os06g05980	transporter family protein, putative, expressed	2.26248	0.651932	0.302887503	1.54997	1.12193
osa-miR3979-3p	3	15	2.2323	10.5185	2.226604058	0.007563544	**	LOC_Os05g38850	MCM8 - Putative minichromosome maintenance MCM family subunit 8, expressed	0.599133	0.742259	0.309046382	1	1
osa-miR1432-5p	141	336	104.9176	235.6145	1.202455295	1.23E-17	**	LOC_Os04g35590	thioesterase family protein, putative, expressed	13.982	17.5442	0.327423424	0.597206746	1
osa-miR531b	0	16	0.01	11.2197	6.98683254	3.07E-05	**	LOC_Os09g33970	expressed protein	3.95079	5.02092	0.345810575	1	1
osa-miR528-3p	21	48	15.626	33.6592	1.138446186	0.002223801	**	LOC_Os02g03040	RNA recognition motif containing protein, expressed	13.33171	16.95206	0.346598759	0.585232725	1
osa-miR1846d-3p	6	17	4.4646	11.921	1.434409235	0.034791993	*	LOC_Os03g62670	expressed protein	0.330674	2.04676	0.356229536	3.1028	0.471074
osa-miR3979-3p	3	15	2.2323	10.5185	2.226604058	0.007563544	**	LOC_Os08g32060	spotted leaf 11, putative, expressed	0.200893	0.257971	0.360781598	1	1
osa-miR531b	0	16	0.01	11.2197	6.98683254	3.07E-05	**	LOC_Os11g04830	embryogenesis transmembrane protein, putative, expressed	0.305316	0.396264	0.376158711	1	1
osa-miR1846d-3p	6	17	4.4646	11.921	1.434409235	0.034791993	*	LOC_Os04g40130	Rf1, mitochondrial precursor, putative, expressed	1.07584	1.5228	0.383490975	1.019	1.06386
osa-miR529b	32	71	23.8111	49.7876	1.097200205	0.000352974	**	LOC_Os08g39890	OsSPL14 - SBP-box gene family member, expressed	3.9919711	5.2494246	0.395058018	1	1
osa-miR531b	0	16	0.01	11.2197	6.98683254	3.07E-05	**	LOC_Os08g37660	plastocyanin-like domain containing protein, putative, expressed	0.950486	1.25954	0.406159657	1	1
osa-miR531b	0	16	0.01	11.2197	6.98683254	3.07E-05	**	LOC_Os12g36210	inhibitor I family protein, putative, expressed	13.7952	18.4476	0.419266764	0.597206671	1
osa-miR1846d-3p	6	17	4.4646	11.921	1.434409235	0.034791993	*	LOC_Os02g47810	dof zinc finger domain containing protein, putative, expressed	2.61015	2.86248	0.423981391	2.94033	2.80897
osa-miR3979-3p	3	15	2.2323	10.5185	2.226604058	0.007563544	**	LOC_Os04g45000	short-chain dehydrogenase/reductase, putative, expressed	8.00058	11.0619	0.467422708	0.647922668	1
osa-miR530-5p	23	67	17.1142	46.9827	1.487976882	1.14E-05	**	LOC_Os11g04520	PP2A regulatory subunit TAP46, putative, expressed	1.44306	2.014763	0.481478856	1	1
osa-miR531b	0	16	0.01	11.2197	6.98683254	3.07E-05	**	LOC_Os04g55240	VQ domain containing protein, putative, expressed	1.58504	2.26578	0.515488538	1	1
osa-miR531b	0	16	0.01	11.2197	6.98683254	3.07E-05	**	LOC_Os01g40640	S1 RNA binding domain containing protein, expressed	4.1546019	6.2156343	0.581191382	0.754029172	1
osa-miR531b	0	16	0.01	11.2197	6.98683254	3.07E-05	**	LOC_Os09g33690	Os9bglu32 - beta-glucosidase homologue, similar to G. max hydroxyisourate hydrolase, expressed	5.49355	8.53642	0.635892422	0.42427977	1
osa-miR531b	0	16	0.01	11.2197	6.98683254	3.07E-05	**	LOC_Os01g39970	protein kinase domain containing protein, putative, expressed	0.587288	0.929869	0.662959324	1	1
osa-miR530-5p	23	67	17.1142	46.9827	1.487976882	1.14E-05	**	LOC_Os07g05010	helix-loop-helix DNA-binding domain containing protein, expressed	2.95864	4.69983	0.66767441	0.72667177	1
osa-miR1846d-3p	6	17	4.4646	11.921	1.434409235	0.034791993	*	LOC_Os09g26760	DNA repair metallo-beta-lactamase family protein, expressed	0.716054	1.151599	0.700295686	0.768998	1.099533
osa-miR531b	0	16	0.01	11.2197	6.98683254	3.07E-05	**	LOC_Os09g20810	strictosidine synthase, putative, expressed	0.628853	1.04607	0.734184677	1	1
osa-miR529b	32	71	23.8111	49.7876	1.097200205	0.000352974	**	LOC_Os09g31438	OsSPL17 - SBP-box gene family member, expressed	3.997693603	6.6670873	0.737888715	0.549053482	1
osa-miR531b	0	16	0.01	11.2197	6.98683254	3.07E-05	**	LOC_Os11g31090	transferase family protein, putative, expressed	36.2125	60.5563	0.74178928	0.014861042	1
osa-miR1846d-3p	6	17	4.4646	11.921	1.434409235	0.034791993	*	LOC_Os02g49760	potassium transporter, putative, expressed	5.394382	3.03676335	0.765570918	2.519778	4.83715
osa-miR531b	0	16	0.01	11.2197	6.98683254	3.07E-05	**	LOC_Os10g18370	transcriptional regulator, putative, expressed	0.816289	1.44931	0.828214292	1	1
osa-miR1846d-3p	6	17	4.4646	11.921	1.434409235	0.034791993	*	LOC_Os01g13720	expressed protein	3.3244	7.97106	0.839304077	4.33621	7.28489
osa-miR530-5p	23	67	17.1142	46.9827	1.487976882	1.14E-05	**	LOC_Os03g52840	serine hydroxymethyltransferase, mitochondrial precursor, putative, expressed	0.458843	0.841412	0.874811795	1	1
osa-miR531b	0	16	0.01	11.2197	6.98683254	3.07E-05	**	LOC_Os12g35610	respiratory burst oxidase, putative, expressed	1.61337	2.977398	0.88397475	1	1
osa-miR531b	0	16	0.01	11.2197	6.98683254	3.07E-05	**	LOC_Os06g05070	protein kinase domain containing protein, expressed	0.219397	0.572994	1.384976233	1	1
osa-miR531b	0	16	0.01	11.2197	6.98683254	3.07E-05	**	LOC_Os09g33690	Os9bglu32 - beta-glucosidase homologue, similar to G. max hydroxyisourate hydrolase, expressed	1.264798	3.860903	1.610031317	0.375124892	1
osa-miR3979-3p	3	15	2.2323	10.5185	2.226604058	0.007563544	**	LOC_Os04g45000	short-chain dehydrogenase/reductase, putative, expressed	0.309511	1.07549	1.796931522	1	1
osa-miR1846d-3p	6	17	4.4646	11.921	1.434409235	0.034791993	*	LOC_Os11g40510	RNA recognition motif containing protein, putative, expressed	1.08619	0.15893	2.189064461	0.242883	0.14515

osa-miR1432-5p	141	336	104.9176	235.6145	1.202455295	1.23E-17	**	LOC_Os03g59770	EF hand family protein, putative, expressed	0.230587	1.20804	2.389285141	1	1
osa-miR531b	0	16	0.01	11.2197	6.98683254	3.07E-05	**	LOC_Os02g45700	fringe-related protein, putative, expressed	0.25106065	1.940791	2.950536935	0.500049959	1
osa-miR1846d-3p	6	17	4.4646	11.921	1.434409235	0.034791993	*	LOC_Os05g38000	ATROPGEF7/ROPGEF7, putative, expressed	0.502965	1.02354	6.45236149	0.00551407	0.793702
osa-miR531b	0	16	0.01	11.2197	6.98683254	3.07E-05	**	LOC_Os01g13740	myb-like DNA-binding domain containing protein, expressed	0.00295567	4.68372	10.62995391	0.062562452	1
osa-miR531b	0	16	0.01	11.2197	6.98683254	3.07E-05	**	LOC_Os01g16390	NB-ARC domain containing protein, expressed	0	0.000100823	Inf	1	1
osa-miR531b	0	16	0.01	11.2197	6.98683254	3.07E-05	**	LOC_Os01g40640	S1 RNA binding domain containing protein, expressed	0	0.713793	Inf	1	1
osa-miR531b	0	16	0.01	11.2197	6.98683254	3.07E-05	**	LOC_Os10g01134	OsSCP46 - Putative Serine Carboxypeptidase homologue, expressed	0	0.575507	Inf	1	1
osa-miR3979-3p	3	15	2.2323	10.5185	2.226604058	0.007563544	**	LOC_Os06g49380	NBS-LRR disease resistance protein, putative, expressed	0	0.0293247	Inf	1	1
osa-miR530-5p	23	67	17.1142	46.9827	1.487976882	1.14E-05	**	LOC_Os11g47980	expressed protein	0	0.0876101	Inf	1	1
osa-miR1846d-3p	6	17	4.4646	11.921	1.434409235	0.034791993	*	LOC_Os03g62670	expressed protein	0	0	Inf	0	0
osa-miR1846d-3p	6	17	4.4646	11.921	1.434409235	0.034791993	*	LOC_Os06g49290	acanthoscurrin-1 precursor, putative, expressed	0.257546	0	Inf	0	0
osa-miR531b	0	16	0.01	11.2197	6.98683254	3.07E-05	**	LOC_Os01g61480	helix-loop-helix DNA-binding domain containing protein, expressed	0	0	NA	1	1
osa-miR531b	0	16	0.01	11.2197	6.98683254	3.07E-05	**	LOC_Os04g31710	expressed protein	0	0	NA	1	1
osa-miR531b	0	16	0.01	11.2197	6.98683254	3.07E-05	**	LOC_Os05g06100	E3 ubiquitin-protein ligase SINA-like 2, putative, expressed	0	0	NA	1	1
osa-miR531b	0	16	0.01	11.2197	6.98683254	3.07E-05	**	LOC_Os05g18940	STIGL3 - Stigma-specific Stig1 family protein precursor, putative, expressed	0	0	NA	1	1
osa-miR531b	0	16	0.01	11.2197	6.98683254	3.07E-05	**	LOC_Os06g01590	lactate/malate dehydrogenase, putative, expressed	0	0	NA	1	1
osa-miR531b	0	16	0.01	11.2197	6.98683254	3.07E-05	**	LOC_Os07g07420	gibberellin 20 oxidase 1-B, putative, expressed	0	0	NA	1	1
osa-miR531b	0	16	0.01	11.2197	6.98683254	3.07E-05	**	LOC_Os07g09560	ankyrin repeat family protein, putative, expressed	0	0	NA	1	1
osa-miR531b	0	16	0.01	11.2197	6.98683254	3.07E-05	**	LOC_Os09g33690	Os9bglu32 - beta-glucosidase homologue, similar to G. max hydroxyisourate hydrolase, expressed	0	0	NA	1	1
osa-miR531b	0	16	0.01	11.2197	6.98683254	3.07E-05	**	LOC_Os11g08410	GATA zinc finger domain containing protein, expressed	0	0	NA	1	1
osa-miR531b	0	16	0.01	11.2197	6.98683254	3.07E-05	**	LOC_Os11g24550	MBTB61 - Bric-a-Brac, Tramtrack, Broad Complex BTB domain with Meprin and TRAF Homology MATH domain, expressed	0	0	NA	1	1
osa-miR1861b	22	206	16.3701	144.4542	3.170325117	7.85E-37	**	LOC_Os09g27150	protein kinase domain containing protein, expressed	0	0	NA	1	1
osa-miR1861f	22	206	16.3701	144.4542	3.170325117	7.85E-37	**	LOC_Os09g27150	protein kinase domain containing protein, expressed	0	0	NA	1	1
osa-miR1861i	22	206	16.3701	144.4542	3.170325117	7.85E-37	**	LOC_Os09g27150	protein kinase domain containing protein, expressed	0	0	NA	1	1
osa-miR1861l	22	206	16.3701	144.4542	3.170325117	7.85E-37	**	LOC_Os09g27150	protein kinase domain containing protein, expressed	0	0	NA	1	1
osa-miR3979-3p	3	15	2.2323	10.5185	2.226604058	0.007563544	**	LOC_Os06g49380	NBS-LRR disease resistance protein, putative, expressed	0	0	NA	1	1
osa-miR3979-3p	3	15	2.2323	10.5185	2.226604058	0.007563544	**	LOC_Os06g49380	NBS-LRR disease resistance protein, putative, expressed	0	0	NA	1	1
osa-miR3979-3p	3	15	2.2323	10.5185	2.226604058	0.007563544	**	LOC_Os06g49380	NBS-LRR disease resistance protein, putative, expressed	0	0	NA	1	1
osa-miR530-5p	23	67	17.1142	46.9827	1.487976882	1.14E-05	**	LOC_Os01g29840	no apical meristem protein, putative, expressed	0	0	NA	1	1
osa-miR530-5p	23	67	17.1142	46.9827	1.487976882	1.14E-05	**	LOC_Os10g29450	expressed protein	0	0	NA	1	1
osa-miR530-5p	23	67	17.1142	46.9827	1.487976882	1.14E-05	**	LOC_Os12g42290	expressed protein	0	0	NA	1	1
osa-miR1432-5p	141	336	104.9176	235.6145	1.202455295	1.23E-17	**	LOC_Os03g59790	EF hand family protein, putative, expressed	0	0	NA	1	1
osa-miR1432-5p	141	336	104.9176	235.6145	1.202455295	1.23E-17	**	LOC_Os03g59870	EF hand family protein, putative, expressed	0	0	NA	1	1
osa-miR528-3p	21	48	15.626	33.6592	1.138446186	0.002223801	**	LOC_Os02g07200	protein-O-fucosyltransferase 1, putative, expressed	0	0	NA	1	1
osa-miR529b	32	71	23.8111	49.7876	1.097200205	0.000352974	**	LOC_Os01g03420	seven in absentia protein family protein, putative, expressed	0	0	NA	1	1
osa-miR529b	32	71	23.8111	49.7876	1.097200205	0.000352974	**	LOC_Os05g49460	CTP synthase, putative, expressed	0	0	NA	1	1
osa-miR529b	32	71	23.8111	49.7876	1.097200205	0.000352974	**	LOC_Os08g34820	OsFBX292 - F-box domain containing protein, expressed	0	0	NA	1	1
osa-miR167c-3p	22	7	16.3701	4.9086	-1.683740609	0.008174891	**	LOC_Os02g52590	xyloglucan fucosyltransferase, putative, expressed	0	0	NA	1	1
osa-miR1846d-3p	6	17	4.4646	11.921	1.434409235	0.034791993	*	LOC_Os01g09870	expressed protein	0	0	NA	0	0
osa-miR1846d-3p	6	17	4.4646	11.921	1.434409235	0.034791993	*	LOC_Os06g35920	expressed protein	0	0	NA	0	0
osa-miR1846d-3p	6	17	4.4646	11.921	1.434409235	0.034791993	*	LOC_Os08g04800	triacylglycerol lipase like	0.292885	0.168909	NA	0	0.419757

									protein, putative, expressed					
osa-miR1846d-3p	6	17	4.4646	11.921	1.434409235	0.034791993	*	LOC_Os08g39220	OsWAK75 - OsWAK receptor-like protein kinase, expressed	0	0	NA	0	0
osa-miR1846d-3p	6	17	4.4646	11.921	1.434409235	0.034791993	*	LOC_Os09g28400	alpha-amylase precursor, putative, expressed	0	0	NA	0	0
osa-miR531a	0	23	0.01	16.1284	7.506928563	2.41E-07	**							
osa-miR531c	0	23	0.01	16.1284	7.506928563	2.41E-07	**							
osa-miR1862a	26	533	19.3465	373.7576	4.301495202	1.59E-117	**							
osa-miR1862b	26	533	19.3465	373.7576	4.301495202	1.59E-117	**							
osa-miR1862c	26	533	19.3465	373.7576	4.301495202	1.59E-117	**							
osa-miR396e-3p	12	41	8.9292	28.7506	1.71276181	0.000130031	**							

Supplemental Table S4. The GO annotation of 94 resistance-related targets exhibiting the opposite expression to 46 differently expressed miRNAs.

Query ID	Query Symbol	Description	E-Value	GO ID	GO Term	P-Value	Enrichment	MicroRNA	Material
LOC_Os02g41800	Os02g0628600	Similar to Auxin response factor 8.	0	GO:0009725	response to hormone	1.30048E-08	14.29602144	osa-miR160e-5p	P15
LOC_Os04g59430.1	Os04g0690600	Similar to Auxin response factor 13.	6E-68	GO:0009725	response to hormone	1.30048E-08	14.29602144	osa-miR160e-5p	P15
LOC_Os06g47150	Os06g0685700	Auxin response factor 18	0	GO:0009725	response to hormone	1.30048E-08	14.29602144	osa-miR160e-5p	P15
LOC_Os02g41800	Os02g0628600	Similar to Auxin response factor 8.	0	GO:0009725	response to hormone	1.30048E-08	14.29602144	osa-miR160f-5p	P15
LOC_Os04g59430.1	Os04g0690600	Similar to Auxin response factor 13.	6E-68	GO:0009725	response to hormone	1.30048E-08	14.29602144	osa-miR160f-5p	P15
LOC_Os06g47150	Os06g0685700	Auxin response factor 18	0	GO:0009725	response to hormone	1.30048E-08	14.29602144	osa-miR160f-5p	P15
LOC_Os06g06050.1	Os06g0154200	F-box/LRR-repeat MAX2 homolog (F-box and leucine-rich repeat MAX2 homolog).	1E-174	GO:0009734	auxin-activated signaling pathway	1.45877E-07	5.207836381	osa-miR172d-5p	P15
LOC_Os05g10580.1	LOC_Os05g10580.1	Hypothetical conserved gene.	4E-49	GO:0009734	auxin-activated signaling pathway	1.45877E-07	5.207836381	osa-miR396c-5p	P15
LOC_Os01g53880	Os01g0741900	Similar to Auxin-responsive protein IAA26 (Indoleacetic acid-induced protein 26) (Phytochrome-associated protein 1).	6E-47	GO:0009734	auxin-activated signaling pathway	1.45877E-07	5.207836381	osa-miR408-3p	P15
LOC_Os11g11410	Os11g0221000	Aux/IAA_ARF_dimerisation domain containing protein.	6E-21	GO:0009734	auxin-activated signaling pathway	1.45877E-07	5.207836381	osa-miR444a-3p.2	P15
LOC_Os11g11410	Os11g0221000	Aux/IAA_ARF_dimerisation domain containing protein.	6E-21	GO:0009734	auxin-activated signaling pathway	1.45877E-07	5.207836381	osa-miR444d.2	P15
LOC_Os11g11410	Os11g0221000	Aux/IAA_ARF_dimerisation domain containing protein.	6E-21	GO:0009734	auxin-activated signaling pathway	1.45877E-07	5.207836381	osa-miR444e	P15
LOC_Os04g56790.1	Os04g0663700	Similar to OSIGBa0099L20.7 protein.	4E-130	GO:0009734	auxin-activated signaling pathway	1.45877E-07	5.207836381	osa-miR6248	PC
LOC_Os06g44970.1	Os06g0660200	Similar to Auxin efflux carrier protein.	0	GO:0009734	auxin-activated signaling pathway	1.45877E-07	5.207836381	osa-miR6248	PC
LOC_Os01g34890.1	LOC_Os01g34890.1	Similar to predicted protein.	0	GO:0052543	callose deposition in cell wall	3.23625E-06	13.1344697	osa-miR408-3p	P15
LOC_Os02g49840	Os02g0731200	Transcription factor MADS57.	2E-70	GO:0052543	callose deposition in cell wall	3.23625E-06	13.1344697	osa-miR444a-3p.1	P15
LOC_Os08g33488.1	Os08g0431900	Transcription factor MADS23.	1E-56	GO:0052543	callose deposition in cell wall	3.23625E-06	13.1344697	osa-miR444a-3p.1	P15
LOC_Os04g23910.1	Os04g0304400	Similar to MADS-box protein AGL16-II.	2E-62	GO:0052543	callose deposition in cell wall	3.23625E-06	13.1344697	osa-miR444a-3p.2	P15
LOC_Os02g49840	Os02g0731200	Transcription factor MADS57.	2E-70	GO:0052543	callose deposition in cell wall	3.23625E-06	13.1344697	osa-miR444a-3p.2	P15
LOC_Os08g33488.1	Os08g0431900	Transcription factor MADS23.	1E-56	GO:0052543	callose deposition in cell wall	3.23625E-06	13.1344697	osa-miR444a-3p.2	P15
LOC_Os02g49840	Os02g0731200	Transcription factor MADS57.	2E-70	GO:0052543	callose deposition in cell wall	3.23625E-06	13.1344697	osa-miR444d.1	P15
LOC_Os08g33488.1	Os08g0431900	Transcription factor MADS23.	1E-56	GO:0052543	callose deposition in cell wall	3.23625E-06	13.1344697	osa-miR444d.1	P15
LOC_Os04g23910.1	Os04g0304400	Similar to MADS-box protein AGL16-II.	2E-62	GO:0052543	callose deposition in cell wall	3.23625E-06	13.1344697	osa-miR444d.2	P15
LOC_Os02g49840	Os02g0731200	Transcription factor MADS57.	2E-70	GO:0052543	callose deposition in cell wall	3.23625E-06	13.1344697	osa-miR444d.2	P15
LOC_Os08g33488.1	Os08g0431900	Transcription factor MADS23.	1E-56	GO:0052543	callose deposition in cell wall	3.23625E-06	13.1344697	osa-miR444d.2	P15
LOC_Os04g23910.1	Os04g0304400	Similar to MADS-box protein AGL16-II.	2E-62	GO:0052543	callose deposition in cell wall	3.23625E-06	13.1344697	osa-miR444e	P15
LOC_Os02g49840	Os02g0731200	Transcription factor MADS57.	2E-70	GO:0052543	callose deposition in cell wall	3.23625E-06	13.1344697	osa-miR444e	P15
LOC_Os08g33488.1	Os08g0431900	Transcription factor MADS23.	1E-56	GO:0052543	callose deposition in cell wall	3.23625E-06	13.1344697	osa-miR444e	P15
LOC_Os02g49840	Os02g0731200	Transcription factor MADS57.	2E-70	GO:0052543	callose deposition in cell wall	3.23625E-06	13.1344697	osa-miR444d.3	PC
LOC_Os03g19590.1	Os03g0309200	Similar to Phytochrome B.	0	GO:0009719	response to endogenous stimulus	0.000122517	1.898768835	osa-miR172d-5p	P15
LOC_Os10g38314.1	LOC_Os10g38314	Glutathione S-transferase, N-terminal domain containing protein	2E-20	GO:0009719	response to endogenous stimulus	0.000122517	1.898768835	osa-miR3979-3p	P15
LOC_Os03g15880	Os03g0265500	Similar to Coronatine-insensitive 1.	0	GO:0009719	response to endogenous stimulus	0.000122517	1.898768835	osa-miR408-5p	P15
LOC_Os07g05010	Os07g0143200	Similar to Helix-loop-helix DNA-binding domain containing protein, expressed.	1E-29	GO:0009719	response to endogenous stimulus	0.000122517	1.898768835	osa-miR530-5p	P15
LOC_Os05g37640.1	Os05g0448700	Conserved hypothetical protein.	3E-29	GO:0009719	response to endogenous stimulus	0.000122517	1.898768835	osa-miR531b	P15
LOC_Os04g51380	Os04g0602900	Similar to cDNA clone:J023087M06, full insert sequence.	1E-66	GO:0009719	response to endogenous stimulus	0.000122517	1.898768835	osa-miR530-3p	P15 and PC
LOC_Os01g12160	Os01g0221100	GH3 auxin-responsive promoter family protein.	0	GO:0009719	response to endogenous stimulus	0.000122517	1.898768835	osa-miR6249a	P15 and PC
LOC_Os02g16540	Os02g0265200	WRKY transcription factor 39.	4E-44	GO:0009719	response to endogenous stimulus	0.000122517	1.898768835	osa-miR6249a	P15 and PC
LOC_Os03g41460.1	Os03g0610900	Serine/threonine-protein kinase SAPK10 (EC 2.7.1.37) (Osmotic stress/abscisic acid-activated protein kinase 10).	9E-176	GO:0009719	response to endogenous stimulus	0.000122517	1.898768835	osa-miR6249a	P15 and PC
LOC_Os03g55530.1	Os03g0764000	Acyl-CoA N-acyltransferase domain containing protein.	1E-142	GO:0009719	response to endogenous stimulus	0.000122517	1.898768835	osa-miR6249a	P15 and PC
LOC_Os04g49510	Os04g0584600	Similar to Calcium dependent protein kinase.	0	GO:0009719	response to endogenous	0.000122517	1.898768835	osa-miR6249a	P15 and PC

					stimulus				
LOC_Os05g32440.1	Os05g0390600	Conserved hypothetical protein.	5E-45	GO:0009719	response to endogenous stimulus	0.000122517	1.898768835	osa-miR6249a	P15 and PC
LOC_Os06g42120.1	Os06g0626600	Sulfotransferase family protein.	1E-71	GO:0009719	response to endogenous stimulus	0.000122517	1.898768835	osa-miR6249a	P15 and PC
LOC_Os11g07040.1	Os11g0171500	Similar to Calcium-dependent protein kinase SK5 (EC 2.7.1.-) (CDPK).	0	GO:0009719	response to endogenous stimulus	0.000122517	1.898768835	osa-miR6249a	P15 and PC
LOC_Os12g07230.1	Os12g0169800	Similar to Calcium-dependent protein kinase SK5 (EC 2.7.1.-) (CDPK).	0	GO:0009719	response to endogenous stimulus	0.000122517	1.898768835	osa-miR6249a	P15 and PC
LOC_Os12g39630.1	Os12g0586100	Serine/threonine-protein kinase SAPK9 (EC 2.7.1.37) (Osmotic stress/abscisic acid-activated protein kinase 9).	4E-173	GO:0009719	response to endogenous stimulus	0.000122517	1.898768835	osa-miR6249a	P15 and PC
LOC_Os01g12160	Os01g0221100	GH3 auxin-responsive promoter family protein.	0	GO:0009719	response to endogenous stimulus	0.000122517	1.898768835	osa-miR6249b	P15 and PC
LOC_Os02g16540	Os02g0265200	WRKY transcription factor 39.	4E-44	GO:0009719	response to endogenous stimulus	0.000122517	1.898768835	osa-miR6249b	P15 and PC
LOC_Os03g41460.1	Os03g0610900	Serine/threonine-protein kinase SAPK10 (EC 2.7.1.37) (Osmotic stress/abscisic acid-activated protein kinase 10).	9E-176	GO:0009719	response to endogenous stimulus	0.000122517	1.898768835	osa-miR6249b	P15 and PC
LOC_Os03g55530.1	Os03g0764000	Acyl-CoA N-acyltransferase domain containing protein.	1E-142	GO:0009719	response to endogenous stimulus	0.000122517	1.898768835	osa-miR6249b	P15 and PC
LOC_Os04g49510	Os04g0584600	Similar to Calcium dependent protein kinase.	0	GO:0009719	response to endogenous stimulus	0.000122517	1.898768835	osa-miR6249b	P15 and PC
LOC_Os05g32440.1	Os05g0390600	Conserved hypothetical protein.	5E-45	GO:0009719	response to endogenous stimulus	0.000122517	1.898768835	osa-miR6249b	P15 and PC
LOC_Os06g42120.1	Os06g0626600	Sulfotransferase family protein.	1E-71	GO:0009719	response to endogenous stimulus	0.000122517	1.898768835	osa-miR6249b	P15 and PC
LOC_Os11g07040.1	Os11g0171500	Similar to Calcium-dependent protein kinase SK5 (EC 2.7.1.-) (CDPK).	0	GO:0009719	response to endogenous stimulus	0.000122517	1.898768835	osa-miR6249b	P15 and PC
LOC_Os12g07230.1	Os12g0169800	Similar to Calcium-dependent protein kinase SK5 (EC 2.7.1.-) (CDPK).	0	GO:0009719	response to endogenous stimulus	0.000122517	1.898768835	osa-miR6249b	P15 and PC
LOC_Os12g39630.1	Os12g0586100	Serine/threonine-protein kinase SAPK9 (EC 2.7.1.37) (Osmotic stress/abscisic acid-activated protein kinase 9).	4E-173	GO:0009719	response to endogenous stimulus	0.000122517	1.898768835	osa-miR6249b	P15 and PC
LOC_Os07g05370.1	Os07g0147600	Protein kinase, core domain containing protein.	0	GO:0009719	response to endogenous stimulus	0.000122517	1.898768835	osa-miR3979-5p	PC
LOC_Os04g12660	LOC_Os04g12660		2E-76	GO:0009733	response to auxin	0.000530668	2.416228976	osa-miR530-3p	P15 and PC
LOC_Os05g02500	Os05g0115800	Similar to MAP kinase phosphatase (Fragment).	2E-47	GO:0000188	inactivation of MAPK activity	0.000875948	20.01443001	osa-miR5144-3p	P15
LOC_Os11g04180.1	Os11g0136800	Similar to predicted protein.	3E-36	GO:0000188	inactivation of MAPK activity	0.000875948	20.01443001	osa-miR1850.1	PC
LOC_Os12g03990.1	Os12g0133700	Similar to Dual specificity protein phosphatase 19 (EC 3.1.3.48) (EC 3.1.3.16) (Stress-activated protein kinase pathway-regulating phosphatase 1) (Protein phosphatase SKRP1). Splice isoform 2.	8E-36	GO:0000188	inactivation of MAPK activity	0.000875948	20.01443001	osa-miR1850.1	PC
LOC_Os09g29890	Os09g0474800	Phosphatidylinositol 3- and 4-kinase, catalytic domain containing protein.	0	GO:0046777	protein autophosphorylation	0.001989388	2.961776639	osa-miR167a-5p	P15
LOC_Os09g29890	Os09g0474800	Phosphatidylinositol 3- and 4-kinase, catalytic domain containing protein.	0	GO:0046777	protein autophosphorylation	0.001989388	2.961776639	osa-miR167b	P15
LOC_Os09g29890	Os09g0474800	Phosphatidylinositol 3- and 4-kinase, catalytic domain containing protein.	0	GO:0046777	protein autophosphorylation	0.001989388	2.961776639	osa-miR167c-5p	P15
LOC_Os07g42250.1	Os07g0614000	Six-bladed beta-propeller, TolB-like domain containing protein.	1E-48	GO:0046777	protein autophosphorylation	0.001989388	2.961776639	osa-miR169r-5p	P15
LOC_Os03g54084.1	Os03g0752100	Similar to Phytochrome C.	0	GO:0046777	protein autophosphorylation	0.001989388	2.961776639	osa-miR172d-5p	P15
LOC_Os11g04170.1	Os11g0136600	Similar to Calcium-dependent protein kinase (Fragment).	0	GO:0046777	protein autophosphorylation	0.001989388	2.961776639	osa-miR6249a	P15 and PC
LOC_Os07g42250.1	Os07g0614000	Six-bladed beta-propeller, TolB-like domain containing protein.	1E-48	GO:0046777	protein autophosphorylation	0.001989388	2.961776639	osa-miR6249a	P15 and PC
LOC_Os11g04170.1	Os11g0136600	Similar to Calcium-dependent protein kinase (Fragment).	0	GO:0046777	protein autophosphorylation	0.001989388	2.961776639	osa-miR6249b	P15 and PC
LOC_Os07g42250.1	Os07g0614000	Six-bladed beta-propeller, TolB-like domain containing protein.	1E-48	GO:0046777	protein autophosphorylation	0.001989388	2.961776639	osa-miR6249b	P15 and PC
LOC_Os05g03920.1	Os05g0130100	Hypothetical conserved gene.	5E-150	GO:0046777	protein autophosphorylation	0.001989388	2.961776639	osa-miR3980a-3p	PC
LOC_Os05g03920.1	Os05g0130100	Hypothetical conserved gene.	5E-150	GO:0046777	protein autophosphorylation	0.001989388	2.961776639	osa-miR3980b-3p	PC
LOC_Os11g04030	Os11g0135000	Similar to Major facilitator superfamily antiporter.	3E-159	GO:0080167	response to karrikin	0.0023833	3.079143079	osa-miR1857-3p	P15
LOC_Os12g03860	Os12g0132800	Major facilitator superfamily antiporter.	2E-159	GO:0080167	response to karrikin	0.0023833	3.079143079	osa-miR1857-3p	P15
LOC_Os01g03530	Os01g0126100	Cupredoxin domain containing protein.	0	GO:0080167	response to karrikin	0.0023833	3.079143079	osa-miR408-3p	P15
LOC_Os03g02300.1	Os03g0114100	Conserved hypothetical protein.	2E-18	GO:0080167	response to karrikin	0.0023833	3.079143079	osa-miR6249a	P15 and PC
LOC_Os03g02300.1	Os03g0114100	Conserved hypothetical protein.	2E-18	GO:0080167	response to karrikin	0.0023833	3.079143079	osa-miR6249b	P15 and PC
LOC_Os02g49760	Os02g0730300	Similar to Potassium transporter HAK3p (Fragment).	0	GO:0071805	potassium ion transmembrane transport	0.002722439	4.618714619	osa-miR1846d-3p	P15
LOC_Os04g32920	Os04g0401700	Potassium transporter 1 (OsHAK1). Splice isoform 2.	0	GO:0071805	potassium ion transmembrane transport	0.002722439	4.618714619	osa-miR6249a	P15 and PC
LOC_Os07g48130	Os07g0679000	Similar to Potassium transporter 2 (AtPOT2) (AtKUP2) (AtKT2).	0	GO:0071805	potassium ion transmembrane transport	0.002722439	4.618714619	osa-miR6249a	P15 and PC
LOC_Os04g32920	Os04g0401700	Potassium transporter 1 (OsHAK1). Splice isoform 2.	0	GO:0071805	potassium ion transmembrane transport	0.002722439	4.618714619	osa-miR6249b	P15 and PC
LOC_Os07g48130	Os07g0679000	Similar to Potassium transporter 2 (AtPOT2)	0	GO:0071805	potassium ion	0.002722439	4.618714619	osa-miR6249b	P15 and PC

		(AtKUP2) (AtKT2).			transmembrane transport				
LOC_Os08g42400	Os08g0535800	No apical meristem (NAM) protein domain containing protein.	1E-30	GO:0010150	leaf senescence	0.003730684	3.752705628	osa-miR169r-3p	P15 and PC
LOC_Os07g28400.1	Os07g0467200	Similar to Clone ZZD536 mRNA sequence.	3E-30	GO:0010150	leaf senescence	0.003730684	3.752705628	osa-miR6249a	P15 and PC
LOC_Os07g28400.1	Os07g0467200	Similar to Clone ZZD536 mRNA sequence.	3E-30	GO:0010150	leaf senescence	0.003730684	3.752705628	osa-miR6249b	P15 and PC
LOC_Os01g49410.1	Os01g0688600	Protein of unknown function DUF1639 family protein.	2E-16	GO:0009693	ethylene biosynthetic process	0.005541594	3.473578763	osa-miR1846d-3p	P15
LOC_Os03g58010.1	Os03g0794200	Similar to N-acetyltransferase.	1E-47	GO:0009693	ethylene biosynthetic process	0.005541594	3.473578763	osa-miR531b	P15
LOC_Os01g09700.1	Os01g0192900	1-aminocyclopropane-1-carboxylate synthase family protein.	2E-173	GO:0009693	ethylene biosynthetic process	0.005541594	3.473578763	osa-miR6249a	P15 and PC
LOC_Os07g42740.1	Os07g0619500	Protein of unknown function DUF1645 family protein.	1E-11	GO:0009693	ethylene biosynthetic process	0.005541594	3.473578763	osa-miR6249a	P15 and PC
LOC_Os01g09700.1	Os01g0192900	1-aminocyclopropane-1-carboxylate synthase family protein.	2E-173	GO:0009693	ethylene biosynthetic process	0.005541594	3.473578763	osa-miR6249b	P15 and PC
LOC_Os07g42740.1	Os07g0619500	Protein of unknown function DUF1645 family protein.	1E-11	GO:0009693	ethylene biosynthetic process	0.005541594	3.473578763	osa-miR6249b	P15 and PC
LOC_Os11g11490.1	Os11g0222000	Regulator of chromosome condensation/beta-lactamase-inhibitor protein II domain containing protein.	2E-137	GO:0009693	ethylene biosynthetic process	0.005541594	3.473578763	osa-miR1846e	PC
LOC_Os07g42740.1	Os07g0619500	Protein of unknown function DUF1645 family protein.	1E-11	GO:0009693	ethylene biosynthetic process	0.005541594	3.473578763	osa-miR3980a-3p	PC
LOC_Os07g42740.1	Os07g0619500	Protein of unknown function DUF1645 family protein.	1E-11	GO:0009693	ethylene biosynthetic process	0.005541594	3.473578763	osa-miR3980b-3p	PC
LOC_Os03g51030	Os03g0719800	Phytochrome A, Photoreceptor	0	GO:0007165	signal transduction	0.005722752	1.565374415	osa-miR172d-5p	P15
LOC_Os04g51400	Os04g0603200	Similar to C-terminal zinc-finger (Fragment).	9E-38	GO:0007165	signal transduction	0.005722752	1.565374415	osa-miR530-5p	P15
LOC_Os12g13300.1	Os12g0235700	Serine/threonine protein kinase-related domain containing protein.	0	GO:0007165	signal transduction	0.005722752	1.565374415	osa-miR531b	P15
LOC_Os01g13800.1	Os01g0239700	Similar to Leucine-rich receptor-like protein kinase.	0	GO:0007165	signal transduction	0.005722752	1.565374415	osa-miR169r-3p	P15 and PC
LOC_Os02g08140.1	Os02g0178000	Similar to SNF1 related protein kinase-like protein.	3E-130	GO:0007165	signal transduction	0.005722752	1.565374415	osa-miR528-3p	P15 and PC
LOC_Os01g60670.1	Os01g0821900	Protein kinase, core domain containing protein.	0	GO:0007165	signal transduction	0.005722752	1.565374415	osa-miR6249a	P15 and PC
LOC_Os05g41200.1	Os05g0491000	EF-Hand type domain containing protein.	2E-32	GO:0007165	signal transduction	0.005722752	1.565374415	osa-miR6249a	P15 and PC
LOC_Os07g48090.1	Os07g0678300	Similar to CBL-interacting protein kinase 29.	3E-98	GO:0007165	signal transduction	0.005722752	1.565374415	osa-miR6249a	P15 and PC
LOC_Os01g60670.1	Os01g0821900	Protein kinase, core domain containing protein.	0	GO:0007165	signal transduction	0.005722752	1.565374415	osa-miR6249b	P15 and PC
LOC_Os05g41200.1	Os05g0491000	EF-Hand type domain containing protein.	2E-32	GO:0007165	signal transduction	0.005722752	1.565374415	osa-miR6249b	P15 and PC
LOC_Os07g48090.1	Os07g0678300	Similar to CBL-interacting protein kinase 29.	3E-98	GO:0007165	signal transduction	0.005722752	1.565374415	osa-miR6249b	P15 and PC
LOC_Os04g51700.1	Os04g0606450	Similar to DNA ligase.	4E-48	GO:0009628	response to abiotic stimulus	0.009309733	1.463756461	osa-miR167d-3p	P15
LOC_Os02g56920.1	Os02g0814200	Similar to CER1-like gene protein (Fragment).	0	GO:0009628	response to abiotic stimulus	0.009309733	1.463756461	osa-miR444a-3p.2	P15
LOC_Os02g56920.1	Os02g0814200	Similar to CER1-like gene protein (Fragment).	0	GO:0009628	response to abiotic stimulus	0.009309733	1.463756461	osa-miR444d.2	P15
LOC_Os02g56920.1	Os02g0814200	Similar to CER1-like gene protein (Fragment).	0	GO:0009628	response to abiotic stimulus	0.009309733	1.463756461	osa-miR444e	P15
LOC_Os11g04520	Os11g0141000	Similar to PP2A regulatory subunit-like protein.	7E-147	GO:0009628	response to abiotic stimulus	0.009309733	1.463756461	osa-miR530-5p	P15
LOC_Os07g36700.1	LOC_Os07g36700.1	Similar to Mixed-linked glucan synthase 2.	0	GO:0009628	response to abiotic stimulus	0.009309733	1.463756461	osa-miR531b	P15
LOC_Os12g35610	Os12g0541300	Similar to Respiratory burst oxidase homolog protein B.	0	GO:0009628	response to abiotic stimulus	0.009309733	1.463756461	osa-miR531b	P15
LOC_Os01g62600.1	LOC_Os01g62600.1		0	GO:0009628	response to abiotic stimulus	0.009309733	1.463756461	osa-miR528-5p	P15 and PC
LOC_Os06g01500	Os06g0104400	Similar to IQ calmodulin-binding motif family protein.	5E-46	GO:0009628	response to abiotic stimulus	0.009309733	1.463756461	osa-miR528-5p	P15 and PC
LOC_Os05g43490	Os05g0510400	Cyclin-like F-box domain containing protein.	3E-113	GO:0009628	response to abiotic stimulus	0.009309733	1.463756461	osa-miR6249a	P15 and PC
LOC_Os06g15170.1	Os06g0262800	Similar to Very-long-chain fatty acid condensing enzyme CUT1.	1E-166	GO:0009628	response to abiotic stimulus	0.009309733	1.463756461	osa-miR6249a	P15 and PC
LOC_Os06g35574.1	LOC_Os06g35574.1	Conserved hypothetical protein.		GO:0009628	response to abiotic stimulus	0.009309733	1.463756461	osa-miR6249a	P15 and PC
LOC_Os12g42280.1	Os12g0617400	Viviparous-14.	0	GO:0009628	response to abiotic stimulus	0.009309733	1.463756461	osa-miR6249a	P15 and PC
LOC_Os05g43490	Os05g0510400	Cyclin-like F-box domain containing protein.	3E-113	GO:0009628	response to abiotic stimulus	0.009309733	1.463756461	osa-miR6249b	P15 and PC
LOC_Os06g15170.1	Os06g0262800	Similar to Very-long-chain fatty acid condensing enzyme CUT1.	1E-166	GO:0009628	response to abiotic stimulus	0.009309733	1.463756461	osa-miR6249b	P15 and PC
LOC_Os06g35574.1	LOC_Os06g35574.1	Conserved hypothetical protein.		GO:0009628	response to abiotic stimulus	0.009309733	1.463756461	osa-miR6249b	P15 and PC
LOC_Os12g42280.1	Os12g0617400	Viviparous-14.	0	GO:0009628	response to abiotic stimulus	0.009309733	1.463756461	osa-miR6249b	P15 and PC
LOC_Os02g34580.1	Os02g0550800	Ammonium transporter family protein.	4E-177	GO:0009867	jasmonic acid mediated signaling pathway	0.011436281	2.010378015	osa-miR172d-5p	P15
LOC_Os09g25070	Os09g0417800	WRKY transcription factor 62.	2E-35	GO:0009867	jasmonic acid mediated signaling pathway	0.011436281	2.010378015	osa-miR6249a	P15 and PC
LOC_Os09g25070	Os09g0417800	WRKY transcription factor 62.	2E-35	GO:0009867	jasmonic acid mediated signaling pathway	0.011436281	2.010378015	osa-miR6249b	P15 and PC
LOC_Os04g43440	LOC_Os04g43440	Similar to NBS-LRR type disease resistance protein O2 (Fragment).	1E-99	GO:0009867	jasmonic acid mediated signaling pathway	0.011436281	2.010378015	osa-miR1846e	PC
LOC_Os01g67650	LOC_Os01g67650		1E-65	GO:0009867	jasmonic acid mediated	0.011436281	2.010378015	osa-miR3979-5p	PC

					signaling pathway					
LOC_Os04g54900.1	LOC_Os04g54900.1	Similar to H0423H10.4 protein.	1E-20	GO:0009740	gibberellic acid mediated signaling pathway	0.027333669	2.729240457	osa-miR3979-3p	P15
LOC_Os07g10620.1	Os07g0206900	Protein of unknown function DUF642 domain containing protein.	4E-100	GO:0009740	gibberellic acid mediated signaling pathway	0.027333669	2.729240457	osa-miR3979-3p	P15
LOC_Os09g02410.1	Os09g0112100	Hypothetical protein.	2E-20	GO:0009863	salicylic acid mediated signaling pathway	0.029318731	2.161558442	osa-miR399a	P15 and PC
LOC_Os09g02410.1	Os09g0112100	Hypothetical protein.	2E-20	GO:0009863	salicylic acid mediated signaling pathway	0.029318731	2.161558442	osa-miR399b	P15 and PC
LOC_Os09g02410.1	Os09g0112100	Hypothetical protein.	2E-20	GO:0009863	salicylic acid mediated signaling pathway	0.029318731	2.161558442	osa-miR399c	P15 and PC
LOC_Os12g36210.1	Os12g0548450	Similar to Proteinase inhibitor.		GO:0009607	response to biotic stimulus	0.0325429	1.514185782	osa-miR531b	P15
LOC_Os12g06210.1	Os12g0158900	Late embryogenesis abundant protein, LEA-14 domain containing protein.	4E-14	GO:0009607	response to biotic stimulus	0.0325429	1.514185782	osa-miR530-3p	P15 and PC
LOC_Os04g36040.1	Os04g0441800	TGF-beta receptor, type I/II extracellular region family protein.	0	GO:0009607	response to biotic stimulus	0.0325429	1.514185782	osa-miR6249a	P15 and PC
LOC_Os04g36040.1	Os04g0441800	TGF-beta receptor, type I/II extracellular region family protein.	0	GO:0009607	response to biotic stimulus	0.0325429	1.514185782	osa-miR6249b	P15 and PC
LOC_Os03g11010.1	Os03g0208500	Integral membrane protein.	0	GO:0009607	response to biotic stimulus	0.0325429	1.514185782	osa-miR1846e	PC
LOC_Os04g45290	Os04g0535600	Similar to Beta-fructofuranosidase 1 precursor (EC 3.2.1.26) (Sucrose 1) (Invertase 1).	1E-169	GO:0009738	abscisic acid-activated signaling pathway	0.038850955	1.833382902	osa-miR408-3p	P15
LOC_Os05g07880	Os05g0171000	Phospholipase D2 (EC 3.1.4.4).	0	GO:0009738	abscisic acid-activated signaling pathway	0.038850955	1.833382902	osa-miR6249a	P15 and PC
LOC_Os05g07880	Os05g0171000	Phospholipase D2 (EC 3.1.4.4).	0	GO:0009738	abscisic acid-activated signaling pathway	0.038850955	1.833382902	osa-miR6249b	P15 and PC
LOC_Os02g12810	Os02g0220700	Hypothetical conserved gene.	0	GO:0016310	phosphorylation	0.046977778	1.316618512	osa-miR167d-3p	P15
LOC_Os04g21110.1	LOC_Os04g21110.1	Hypothetical conserved gene.	0	GO:0016310	phosphorylation	0.046977778	1.316618512	osa-miR172d-5p	P15
LOC_Os06g05070.1	Os06g0142650	Similar to Avr9/Cf-9 rapidly elicited protein 11 (Fragment).	9E-106	GO:0016310	phosphorylation	0.046977778	1.316618512	osa-miR531b	P15
LOC_Os03g53230.1	Os03g0743900	Similar to ATP sulfurylase.	0	GO:0016310	phosphorylation	0.046977778	1.316618512	osa-miR395l	P15 and PC
LOC_Os03g53230.1	Os03g0743900	Similar to ATP sulfurylase.	0	GO:0016310	phosphorylation	0.046977778	1.316618512	osa-miR395m	P15 and PC
LOC_Os03g53230.1	Os03g0743900	Similar to ATP sulfurylase.	0	GO:0016310	phosphorylation	0.046977778	1.316618512	osa-miR395n	P15 and PC
LOC_Os03g53230.1	Os03g0743900	Similar to ATP sulfurylase.	0	GO:0016310	phosphorylation	0.046977778	1.316618512	osa-miR395p	P15 and PC
LOC_Os03g53230.1	Os03g0743900	Similar to ATP sulfurylase.	0	GO:0016310	phosphorylation	0.046977778	1.316618512	osa-miR395q	P15 and PC
LOC_Os03g53230.1	Os03g0743900	Similar to ATP sulfurylase.	0	GO:0016310	phosphorylation	0.046977778	1.316618512	osa-miR395r	P15 and PC
LOC_Os02g17910.1	Os02g0280400	Similar to casein kinase I isoform delta-like.	5E-164	GO:0016310	phosphorylation	0.046977778	1.316618512	osa-miR399a	P15 and PC
LOC_Os02g17910.1	Os02g0280400	Similar to casein kinase I isoform delta-like.	5E-164	GO:0016310	phosphorylation	0.046977778	1.316618512	osa-miR399b	P15 and PC
LOC_Os02g17910.1	Os02g0280400	Similar to casein kinase I isoform delta-like.	5E-164	GO:0016310	phosphorylation	0.046977778	1.316618512	osa-miR399c	P15 and PC
LOC_Os02g17910.1	Os02g0280400	Similar to casein kinase I isoform delta-like.	5E-164	GO:0016310	phosphorylation	0.046977778	1.316618512	osa-miR399d	P15 and PC
LOC_Os04g41100	Os04g0488000	Similar to PITSLRE serine/threonine-protein kinase CDC2L1 (EC 2.7.1.37) (Galactosyltransferase associated protein kinase p58/GTA) (Cell division cycle 2-like protein kinase 1).	0	GO:0016310	phosphorylation	0.046977778	1.316618512	osa-miR530-3p	P15 and PC
LOC_Os01g59360.1	Os01g0808400	Similar to Calcium-dependent protein kinase (Fragment).	0	GO:0016310	phosphorylation	0.046977778	1.316618512	osa-miR6249a	P15 and PC
LOC_Os03g60810.1	Os03g0823000	Resistance protein candidate (Fragment).	0	GO:0016310	phosphorylation	0.046977778	1.316618512	osa-miR6249a	P15 and PC
LOC_Os01g59360.1	Os01g0808400	Similar to Calcium-dependent protein kinase (Fragment).	0	GO:0016310	phosphorylation	0.046977778	1.316618512	osa-miR6249b	P15 and PC
LOC_Os03g60810.1	Os03g0823000	Resistance protein candidate (Fragment).	0	GO:0016310	phosphorylation	0.046977778	1.316618512	osa-miR6249b	P15 and PC
LOC_Os05g34390.1	LOC_Os05g34390.1		2E-70	GO:0016310	phosphorylation	0.046977778	1.316618512	osa-miR166k-5p	PC

Supplemental Table S5. Primers used for qRT-PCR and transformation of rice protoplasts.

Primers used for qRT-PCR.

Primer Name	Primer Sequences
SL1-miR166a-3p	GTCGTATCCAGTGCAGGGTCCGAGGTATTCGCACTGGATACGACGGGGAAT
Q1-miR166a-3p	ccTCGGACCAGGCTTCATT
SL2-miR169h	GTCGTATCCAGTGCAGGGTCCGAGGTATTCGCACTGGATACGACCAGGCAA
Q2-miR169h	cggcTAGCCAAGGATGACTT
SL3-miR168a-3p	GTCGTATCCAGTGCAGGGTCCGAGGTATTCGCACTGGATACGACATTCACT
Q3-miR168a-3p	ATGATCCCGCCTTGCACCAA
SL4-miR396c-5p	GTCGTATCCAGTGCAGGGTCCGAGGTATTCGCACTGGATACGACAAGTTCA
Q4-miR396c-5p	ccgcTTCCACAGCTTTCTTG
SL5-miR399d	GTCGTATCCAGTGCAGGGTCCGAGGTATTCGCACTGGATACGACCAGGGCA
Q5-miR399d	gcgTGCCAAAGGAGAGTTG
SL6-miR444e	GTCGTATCCAGTGCAGGGTCCGAGGTATTCGCACTGGATACGACAAGCTTG
Q6-miR444e	TTCCTTGCAGTTGCTGCCTC
SL7-miR827	GTCGTATCCAGTGCAGGGTCCGAGGTATTCGCACTGGATACGACTGTTTGC
Q7-miR827	gccggTTAGATGACCATCAGC
SL8-miR1425-5p	GTCGTATCCAGTGCAGGGTCCGAGGTATTCGCACTGGATACGACAGCAGCA
Q8-miR1425-5p	gcgccTAGGATTCAATCCTT
SL9-MIR8155-y	GTCGTATCCAGTGCAGGGTCCGAGGTATTCGCACTGGATACGACTGGTATC
Q9-MIR8155-y	gcgcAACCTGGCTCTGA
SL10-MIR5168-y	GTCGTATCCAGTGCAGGGTCCGAGGTATTCGCACTGGATACGACGAAGGGA
Q10-MIR5168-y	atgTCGGACCAGGCTTCAATC
SL11-MIR8175-y	GTCGTATCCAGTGCAGGGTCCGAGGTATTCGCACTGGATACGACTGGCGCC
Q11-MIR8175-y	atgcTCCCCGGCAACGG
SL12-novel-m0131-5p	GTCGTATCCAGTGCAGGGTCCGAGGTATTCGCACTGGATACGACTAAGACG
Q12-novel-m0131-5p	ggggTAACGTTTGACTACTCG
SL13-novel-m0269-3p	GTCGTATCCAGTGCAGGGTCCGAGGTATTCGCACTGGATACGACACTCCCT
Q13-novel-m0269-3p	ggggTTTTTTTTCTAGGACAGAG
SL14-novel-m0465-3p	GTCGTATCCAGTGCAGGGTCCGAGGTATTCGCACTGGATACGACGTTTTTA
Q14-novel-m0465-3p	ggggTCAAACGTTGCAAGTA
SL15-novel-m0017-3p	GTCGTATCCAGTGCAGGGTCCGAGGTATTCGCACTGGATACGACAAGAGCA
Q15-novel-m0017-3p	ggggTCAAGGACCGTAGAATTG
SL16-novel-m0073-5p	GTCGTATCCAGTGCAGGGTCCGAGGTATTCGCACTGGATACGACGGATAAT
Q16-novel-m0073-5p	atgcTCATTGGCGATCGGAT
SL17-novel-m0546-3p	GTCGTATCCAGTGCAGGGTCCGAGGTATTCGCACTGGATACGACAGTCAAA
Q17-novel-m0546-3p	ggccgcgTATTATAAGACGTTT
SL18-novel-m0494-3p	GTCGTATCCAGTGCAGGGTCCGAGGTATTCGCACTGGATACGACAGTTTTT
Q18-novel-m0494-3p	ggggTTAAACGTTGCAAGTAA
U6-F	TACAGATAAGATTAGCATGGCCCC
U6-R	GGACCATTTCTCGATTTGTACGTG
SL-universal R	GTGCAGGGTCCGAGGTATTC

Primers used for transformation of rice protoplasts.

Primer Name	Primer Sequences
miR160f-5pF	TACCACTGCTACTCTGCCTG
miR160f-5pR	AGTGCAAAATAGAGGATCGCA
miR167a-5pF	GCCAAGCCTGAAACACTTTG
miR167a-5pR	ACATACACTGAGAAATCCAGCA
ARF16-FSpe1	GACTAGTATGATCACGTTCGCGGACCT
ARF16-RSpe1	GACTAGTGCTCCCTAAGTTGTCGCTCC
Os07g29820FXmaJ1	CCCTAGGATGGAGACAATTCTAGCTGG
Os07g29820RXmaJ1	CCCTAGGATGTGAAGAAGCATGCGTGG
